# Logical regulation of endogenous gene expression using programmable, multi-input processing CRISPR guide RNAs

**DOI:** 10.1093/nar/gkae549

**Published:** 2024-06-29

**Authors:** Hansol Kang, Dongwon Park, Jongmin Kim

**Affiliations:** Department of Life Sciences, Pohang University of Science and Technology, Pohang 37673, Korea; Department of Life Sciences, Pohang University of Science and Technology, Pohang 37673, Korea; Department of Life Sciences, Pohang University of Science and Technology, Pohang 37673, Korea

## Abstract

The CRISPR-Cas system provides a versatile RNA-guided approach for a broad range of applications. Thanks to advances in RNA synthetic biology, the engineering of guide RNAs (gRNAs) has enabled the conditional control of the CRISPR-Cas system. However, achieving precise regulation of the CRISPR-Cas system for efficient modulation of internal metabolic processes remains challenging. In this work, we developed a robust dCas9 regulator with engineered conditional gRNAs to enable tight control of endogenous genes. Our conditional gRNAs in *Escherichia coli* can control gene expression upon specific interaction with trigger RNAs with a dynamic range as high as 130-fold, evaluating up to a three-input logic A OR (B AND C). The conditional gRNA-mediated targeting of endogenous metabolic genes, *lacZ*, *malT* and *poxB*, caused differential regulation of growth in *Escherichia coli* via metabolic flux control. Further, conditional gRNAs could regulate essential cytoskeleton genes, *ftsZ* and *mreB*, to control cell filamentation and division. Finally, three types of two-input logic gates could be applied for the conditional control of *ftsZ* regulation, resulting in morphological changes. The successful operation and application of conditional gRNAs based on programmable RNA interactions suggests that our system could be compatible with other Cas-effectors and implemented in other host organisms.

## Graphical Abstract

**Graphical Abstract:**
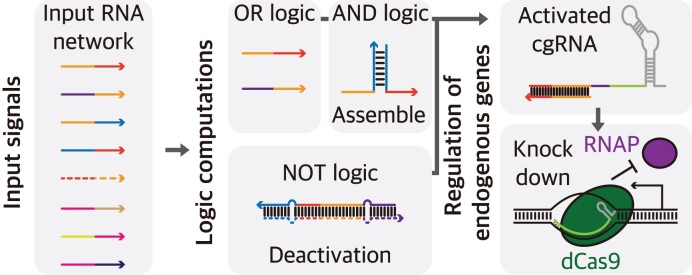

## Introduction

The aim of biocomputing is to rationally program cellular activities by connecting parts that execute predefined, programmable features analogous to those of electronic circuits (1). Just as Boolean logic gates are widely employed in electronic circuits to create digital devices, natural and synthetic gene regulatory networks show features of logic operations and switch-like behaviors (2–4). These networks enable cells to process and integrate diverse signals from environment and internal processes, facilitating the execution of programmed response for increased fitness. For example, *Escherichia coli* employs the logic of Lactose AND (NOT Glucose) to control metabolic enzymes that results in preferential utilization of glucose even in the presence of lactose (5). A library of high-performance regulatory components with modular and composable characteristics is required to construct sophisticated synthetic gene circuits (6–8). Especially promising is the new developments in RNA-based regulatory elements due to their ability to undergo predictable base pairing and ease of design, making them well-suited for scaling up and integrating synthetic gene circuits (9).

RNA-based regulatory elements have been developed to implement Boolean logic gates and control cellular metabolism. For instance, bacterial small RNAs have been utilized for multiplexed modulation of metabolism (10,11). Other RNA regulatory elements, such as riboswitches, ribozymes, or RNA aptamers, have been engineered for logical sensing of biomolecules for gene regulation (12,13). The wealth of natural and synthetic RNA regulatory elements was further enriched with the introduction of *de novo* designed RNA elements featuring entirely synthetic mode of gene regulation (14,15), providing a powerful platform for multi-input Boolean logic computation in cells (16–18). Despite recent progress, challenges remain in developing a versatile system to control endogenous metabolism and scale up sophisticated cellular regulatory networks.

The application of CRISPR technologies has rapidly expanded since its first demonstration as a genome editing tool (19–22). Repurposing CRISPR/Cas system through modification of guide RNAs (gRNAs) and fusion of effector proteins produced sophisticated genetic circuits such as multiplexed transcriptional regulation and reprogramming of the epigenetic state of a cell, altering cell fates (23–25). Since Cas effector proteins are guided by RNAs, controlling Cas protein activity by conformational change of gRNA provides several advantages for developing sophisticated genetic circuits (26–28). First, gRNAs can be engineered to be activated by detecting a diverse set of biological signals (29–34). Second, the compact size of gRNAs makes them suitable for the construction of multi-input multi-output logic gates (7,35,36). Still, simultaneously achieving the desired characteristics in terms of dynamic range, tunability and multiplexing capability under the same design principle remains a challenge, often requiring additional solutions to address limited performance (37,38).

Here, we studied rational design of multi-input Boolean logic computing using conditional gRNAs (cgRNAs) and its application for orthogonal regulation of endogenous gene expression in a predefined manner. By optimizing the secondary structure of the RNA scaffold and the molecular ratio of the components, we achieved a fold reduction of up to 130-fold in *E. coli*. Our results demonstrate that two-input AND, OR, and NOT gates can be evaluated with a dynamic range of up to 120-fold and could be combined to form a three-input logic expression. CgRNAs could be applied to regulate the endogenous *ftsZ*, *mreB*, *lacZ*, *malT* and *poxB* genes for multiple metabolic pathways. The two-input logic gates were then employed to control the expression of *ftsZ* to induce morphological changes. In conclusion, our cgRNA exhibits a large dynamic range and the capability to process complex logic in cells.

## Materials and methods

### Materials, strains and growth conditions

DNA primers were purchased from Bionics (Seoul, Korea). Reagents used for *E. coli* cultivation were purchased from Gibco (Waltham, MA, USA). *E. coli* strains DH5α (*endA1 recA1 gyrA96 thi-1 glnV44 relA1 hsdR17* (r_K_^−^ m_K_^+^) λ^−^) and BL21-AI™ (F^−^*omp*T *hsd*S_B_ (r_B_^−^ m_B_^−^) *gal dcm ara*B::T7RNAP-*tet*A) were obtained from Enzynomics (Daejeon, Korea) and Invitrogen (Carlsbad, CA, USA). All strains were grown in Luria-Bertani (LB) medium at 37°C with appropriate antibiotics: Kanamycin (30 μg/ml), Ampicillin (50 μg/ml), Spectinomycin (25 μg/ml) and Chloramphenicol (17 μg/ml).

### Plasmid construction

The backbones of the plasmids used in this study were derived from the commercial vectors pET15b, pCDFDuet or pACYCDuet (EMD Millipore, Burlington, MA, USA). All trigger RNAs and non-cognate decoys of cgRNAs were constructed in pET15b and/or pCDFDuet. CgRNAs and dCas9 were constructed in pACYCDuet. Target GFP was constructed in pSC101 (Addgene plasmid #103057) (39). All constructs were cloned by Gibson Assembly (40) and round-the-horn site-directed mutagenesis (41). Plasmid architecture and specific partial sequences are listed in Supplementary Tables S1–S12. Plasmids were constructed in *E. coli* DH5α and purified using the EZ-Pure™ Plasmid Prep Kit. Ver. 2 (Enzynomics, Daejeon, Korea). Plasmid sequences were confirmed by DNA sequencing. Plasmids were transformed in *E. coli* by chemical transformation.

### Flow cytometry measurement

GFP fluorescence was measured by flow cytometry (CytoFLEX S, Beckman Coulter, Brea, CA, USA) after fixation at the Microbiome Core Research Support Center of the Korea Basic Science Institute (KBSI). The cell pellet was resuspended with para-formaldehyde solution and fixed for 15 min at room temperature. After fixation, samples were washed twice with 1× phosphate buffered saline (PBS). Fixed cells were diluted by a factor of ∼5-fold in 1× PBS. Cells were detected using a forward scatter (FSC) trigger and a minimum of 30 000 events were recorded for each measurement. The cell population was gated according to the FSC and GFP fluorescence (measured in FITC channel) distributions. To evaluate circuit performance, GFPmut3b-ASV fluorescence was measured on a FITC channel, excited at 488 nm, and detected with a 525 nm/40 nm bandpass filter. GFP fluorescence histograms yielded unimodal population distributions, and the geometric mean was used for the average fluorescence over the approximately log-normal fluorescence distribution of three biological replicates. In the case of *ftsZ* regulation, 0–10% of GFP-negative and smaller cells were found, even though they were designed to express GFP constitutively. These cells were considered disrupted in metabolic states, and were subsequently gated out. Fold reductions for GFP fluorescence were calculated from flow cytometry data by taking the geometric mean fluorescence values from the inactive cgRNA (switch with a non-cognate trigger) and the active cgRNA (switch with a cognate trigger) measurements. Autofluorescence was not subtracted from either fluorescence value before calculating the fold reduction. P-values were presented as statistical analysis by the *t*-test.


\begin{eqnarray*}{\mathrm{Fold\;reduction}} = \frac{{{\mathrm{GFP\;fluorescence\;of\;cgRNA\;with\;decoy}}}}{{{\mathrm{GFP\;fluorescence\;of\;cgRNA\;with\;trigger}}}}\end{eqnarray*}

### Conditional guide RNA induction conditions

For all experiments, transformed BL21-AI^TM^ cells were grown overnight (∼16 h) in 96-well plates with shaker (800 r.p.m.). Overnight cultures were diluted 100-fold into fresh LB media and returned to the shaker (800 r.p.m.). Unless otherwise noted, cell was grown for 1 h and 20 min. CgRNAs and trigger RNAs were induced with appropriate inducers for each experiment as described. Except for the growth profiling experiment, they were then returned to the shaker (800 r.p.m.) until collection for further analysis after 3 h and 30 min.

### Control of GFP expression

For logic-gated GFP regulation using dCas9 or dCas9-effector (fused with transcription activating protein) with a cgRNA, cells were induced with different conditions for each experiment: 2 mg/ml l-arabinose for trigger expression, 0.5 ng/ml anhydrotetracycline (aTc) for cgRNA and dCas9 expression, and 0.1 mM isopropyl β-d-1-thiogalactopyranoside (IPTG) for GFP expression. In the case of NOT logic gate, the aTc concentration was adjusted to 0.1 ng/ml. The difference in optimal aTc concentration may in part be explained by the different mechanism of the logic gate (ON-to-OFF rather than OFF-to-ON).

### Control of cell morphology

Cells were induced with following conditions for each experiment: 2 mg/ml l-arabinose, 0.5 ng/ml aTc and 0.1 mM IPTG for expression of GFP as reporters in fluorescence imaging and for gating cells in flow cytometry analysis. In the case of 2-input A AND NOT B logic gate-mediated *ftsZ* regulation, the aTc concentration was adjusted to 0.4 ng/ml. In the case of two-input AND logic gate mediated *ftsZ* regulation, the induction time was extended to 7 h, due in part to slower kinetics of AND-logic gate trigger assembly as well as phenotypic changes that reflect gene expression changes with a delay. Subsequently, 1 μl of cell cultures were diluted with PBS and mounted with clotted agarose gel to observe cell shape by fluorescence imaging using Zeiss Axio Scope with EC PLAN NEOFLUAR. Digital images were captured with AxioCam HRM camera and processed with AxioVision 4.8 software using fluorescence microscope as described above. Images were analyzed in at least three independent areas per sample.

### Control of lactose metabolism

For induction, appropriate concentrations of aTc (0.5 ng/ml), IPTG (0.125 mM) for *lacZ* induction, l-arabinose (2 mg/ml) and X-gal (200 μg/ml) were added and incubated for 4 h and 30 min. After induction, samples were centrifuged to remove LB broth and treated with lysis buffer for homogenization.

### Growth profiling experiments of conditional metabolic regulation (lactose, maltose and acetate)

For the conditional metabolic regulation, single colonies were inoculated into 1000 μl M9 medium with 4 mg/ml glucose and grown overnight (∼16 h at 800 r.p.m.).

For lactose or maltose metabolic regulation, overnight cultures were diluted 100-fold into fresh M9 medium containing 2 mg/ml lactose or maltose and appropriate antibiotics. For each colony, three 200 μl parallel cultures were prepared and transferred to 96-well microtiter plates for growth profiling. For induction, 2 mg/ml l-arabinose and 0.5 ng/ml aTc were used and then prepared for further analysis.

For the regulation of acetate metabolism, overnight cultures were diluted 100-fold into fresh M9 medium containing 4 mg/ml glucose. The pH of all M9 media was adjusted to 7.4. For each colony, three 200 μl parallel cultures were prepared and transferred to 96-well microtiter plates for growth profiling. For induction, 2 mg/ml l-arabinose, 1 ng/ml aTc and 0.125 mM IPTG were used and then prepared for further analysis.

After induction, cells were cultured in a microplate reader (BioTek Gen5, Santa Clara, CA, USA) at 37°C with double orbital shaking. OD at 600 nm was recorded for each well at 5 min intervals for up to 16 h. Growth curves were plotted from three independent colonies. The sequences used in this experiment are listed in Supplementary Tables S8–S9.

### Reversibility of cgRNA-based regulation for *gfp, lacZ* and *ftsZ*

For the reversible control of gene expression, the cell culture conditions were adjusted because stationary phase *E. coli* showed reduced responsiveness to inducer inputs. The OD_600_ of the cell cultures was continuously tracked with additional fresh media containing inducers to ensure that the cell cultures were maintained in early to mid log phase (OD_600_ < 0.5). A 200 μl sample of the cell culture was taken for analysis and processed for further analysis.

For *ftsZ* regulation, 100-fold diluted cells were grown for 30 min and induced for 3 h with L-arabinose to induce trigger RNA expression. The inducer (l-arabinose) was then washed out and the cells were cultured for an additional 3 h, with 200 μl of cells sampled and replenished every hour with 200 μl of fresh LB media containing 0.1 mM IPTG for GFP expression to maintain the cultures in a mid-log phase. Sampled cells were prepared for fluorescence imaging and flow cytometry analysis.

### Quantitative PCR for determination of RNA expression levels in *E. coli*

To extract bacterial RNA, cultured cells were diluted 100-fold into M9 media with antibiotics. Induction was performed for 3 h and 30 min under DNase and RNase-free conditions. For each experimental condition, 1500 μl of cells were collected into a 1.6 ml tube from three wells (500 μl each). Cells were pelleted by centrifugation at 13 000 r.p.m. for 3 min. The pellet was resuspended in 1000 μl of RiboEx reagent (GeneAll, Seoul, Korea) and stored at -80°C overnight. After thawing on ice and vortexing, samples were homogenized at room temperature for 5 min.

Chloroform (200 μl) was added to the samples, mixed briefly, and incubated at room temperature for 3 min. After centrifugation at 12 000 r.p.m. for 15 min at 4°C, 400 μl of the upper aqueous layer was collected and mixed with 500 μl of isopropanol. The mixture was smoothly inverted 10 times and incubated at room temperature for 10 min and then centrifuged at 13 000 r.p.m. at 4°C for 15 min. The isopropanol was carefully removed, and RNA pellets were washed in 1000 μl of chilled 70% ethanol. After removing ethanol, pellets were air dried for 10 min in 60°C oven and resuspended in 20 μl of RNase-free double-distilled water. For cDNA synthesis, 1 μg of total RNA was reverse transcribed using the cDNA Synthesis Kit (Promega, Madison, WI, USA). The RT reaction was performed at 37°C for 15 min, followed by inactivation of reverse transcriptase at 85°C for 15 s using a thermal cycler.

qPCR experiments were conducted using ORA™ qPCR Green ROX L Mix (2×) (highQu, Kraichtal, Germany) with specific qPCR primers for target RNA or 16S rRNA. A Stratagene Mx3000P thermal cycler was used with specified conditions. Results were analyzed using the MxPRO software (Agilent Technologies, Santa Clara, CA, USA). Primer sequences are listed in Supplementary Table S10.

## Results

### Characterization and optimization of cgRNA for CRISPR interference

**Figure 1.**
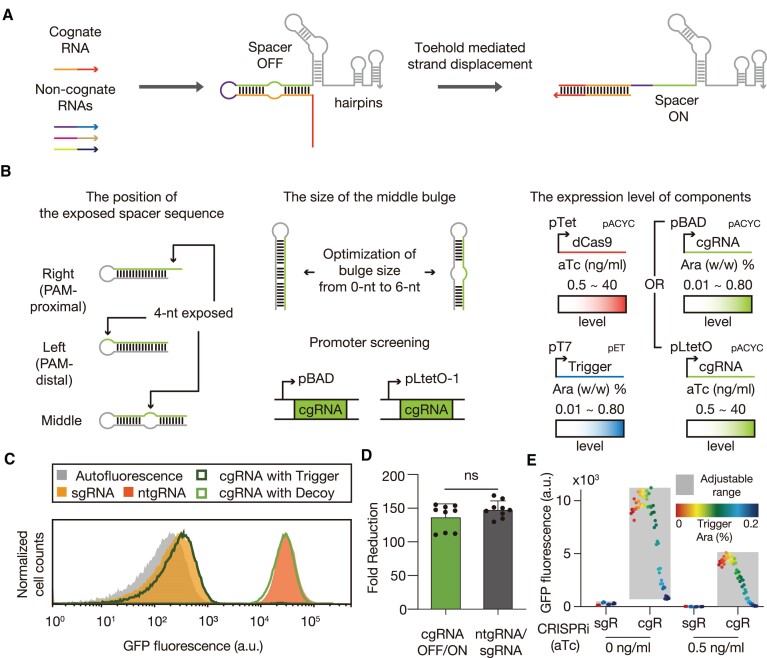
Schematic of RNA-responsive cgRNA and characterization *in vivo*. (**A**) An RNA-responsive cgRNA blocks its spacer sequence through the *cis-*regulatory sequence of the extended 5′ end. Upon the introduction of cognate trigger RNA, the interaction between the cgRNA and trigger RNA leads to the strand displacement process. The resulting exposed spacer sequence is now able to initiate an interaction between the cgRNA-trigger complex and the target DNA. (**B**) Characterization of various design factors for construction of an optimized cgRNA system: the position of exposed spacer sequence, the size of the middle bulge, and the stochiometry of cgRNA and input RNAs. For Supplementary Figures S3, S4, S6, S7 and S9, cgRNAs were controlled by pBAD. In all other experiments, cgRNAs were controlled by pLtetO-1. (**C**) Flow cytometry analysis of GFP fluorescence of *E. coli* expressing cgRNA with the trigger RNA, decoy RNA or controls. Autofluorescence was obtained for *E. coli* not bearing plasmid. A single gRNA (sgRNA) and a non-target gRNA (ntgRNA) without spacer sequence were used to analyze the experimental maximum and minimum of fluorescence levels for the gRNA design. (**D**) Fold reduction of the output of the single input cgRNA compared with that of the control group (an sgRNA and an ntgRNA). (**E**) Adjustable ranges of GFP expressions of *E. coli* cells with either cgRNA or sgRNA in the presence of trigger RNA. l-arabinose (ara) upregulates transcription of trigger RNA, while anhydrotetracycline (aTc) upregulates expression of both dCas9 and gRNA (sgR: sgRNA, cgR: cgRNA). The number of biological replicates is three, and the error bars for fold reductions are the s.d. of nine possible fold reductions for inactive and active cgRNA states. Based on Welch's *t-*test, ns: not significant.

A CRISPR gRNA acquires target specificity by the spacer, which has a complementary sequence to the target DNA, allowing for Watson-Crick base pairing. Upon recognition of the protospacer adjacent motif (PAM) by Cas9, the spacer fully unwinds the partially melted double stranded DNA of the target region, starting from the PAM-proximal seed sequence (42). The 5′ region of a single gRNA (sgRNA) was extended to incorporate a spacer complementary sequence and a toehold region, such that the hidden spacer domain can be targeted by other RNA inputs. These RNA-responsive cgRNA designs were shown to be functional in a diverse set of conditions in different host cells (43,44). In the absence of an input RNA, a cgRNA stays inactive, while in the presence of an input RNA, a toehold mediated strand displacement reveals the previously sequestered spacer domain, rendering the cgRNA active (Figure 1A). We initially designed three cgRNAs with varying spacer sequestering structures (Figure 1B) to compare the dynamic range and leakage of each design (Supplementary Figures S1–S3). A single design candidate showed best performance with a low leakage in its inactive state and a strong repression in its active state (Supplementary Figure S4). This design was further characterized by varying the bulge size from 0 to 6 nucleotides. The designs with small bulges exhibited tight leakage control, while those with large bulges knocked down the target gene as well as an sgRNA upon activation (Supplementary Figures S5–S7). The toehold-binding and strand-invasion domain lengths for trigger RNAs were screened and optimized for cgRNA activation (Supplementary Figure S8). The cgRNA sequence design candidates with low ensemble defect evaluated by NUPACK and without undesired secondary structure formation evaluated by RNAstructure were selected for experimental validation (Supplementary Information) (45–47).

The 4-nt bulge variant was chosen since this design showed both tight leakage control and strong repression upon activation. For experimental validation, we designed a set of expression cassettes with different inducible promoter combinations to adjust expression levels of each component in the system. The cgRNA was cloned under the control of pBAD promoter (induced by L-arabinose) or pLtetO-1 promoter (induced by aTc) in pACYCDuet backbone together with a dCas9 cassette. The expression of dCas9 with an SsrA tag variant (ec-AAV) at its C-terminus (48) was controlled by Tet promoter (induced by aTc). Finally, the expression of trigger RNAs was driven by a strong T7 promoter (induced by l-arabinose in BL21-AI strain) cloned in pET-15b or pCDFDuet backbone. Inducer concentrations were adjusted to balance expression levels of cgRNA and input RNAs for each experiment (Figure 1B right panel, and Supplementary Figure S9a–c, and Supplementary Table S13 for detailed induction conditions). Through optimization, the activated cgRNA could repress fluorescence reporter output almost as strongly as an sgRNA, while almost no repression was observed in its inactive form analogous to a non-target gRNA (ntgRNA) without a spacer domain (Figure 1B). The fold reduction was improved up to 137.26-fold after 3 h 30 min induction (Figure 1C). The dynamic range of optimized cgRNA was not significantly different from that of a pair of positive (sgRNA) and negative controls (ntgRNA) (Figure 1D, Supplementary Figure S9c). Time-course measurement of GFP fluorescence revealed that the GFP fold-reduction increased up to 280-fold at 4 h 30 min after induction and then subsequently decreased as the cell culture reaches a stationary phase (Supplementary Figure S10a–c). To evaluate the reversibility of cgRNA-mediated gene regulation, we continuously tracked GFP expression during the sequential activation, deactivation, and reactivation of cgRNA through repeated addition and removal of l-arabinose that controls the trigger RNA expression (Supplementary Figure S10d–f). The cgRNA activity could be switched reversibly simply by controlling the trigger RNA expression. When the cgRNA design was compared to its sgRNA design counterparts, cgRNA activity could be adjusted via input RNA levels (Figure 1E and Supplementary Figure S11a–c). This remarkable performance has prompted us to investigate the feasibility of building essential input-processing genetic circuits and combining them to more sophisticated biocomputing networks, by implementing Boolean logic computation.

### Multi-input logic operations using cgRNA

To expand the potential of cgRNA-based CRISPR/Cas regulation, we next investigated modularity and programmability of the design by building Boolean logic gates. Various CRISPR/Cas regulatory elements have achieved multi-input processing devices (7,49–51). However, generating functionally complete set of Boolean logic operators and combining them to regulate endogenous metabolic circuits remains elusive. Recent RNA-based regulators coupled with toehold-mediated strand displacement reactions and RNA self-assembly have provided modular multi-input processing gene circuits (52). Therefore, we applied similar strategies to build logic circuits in living cells (17).

**Figure 2.**
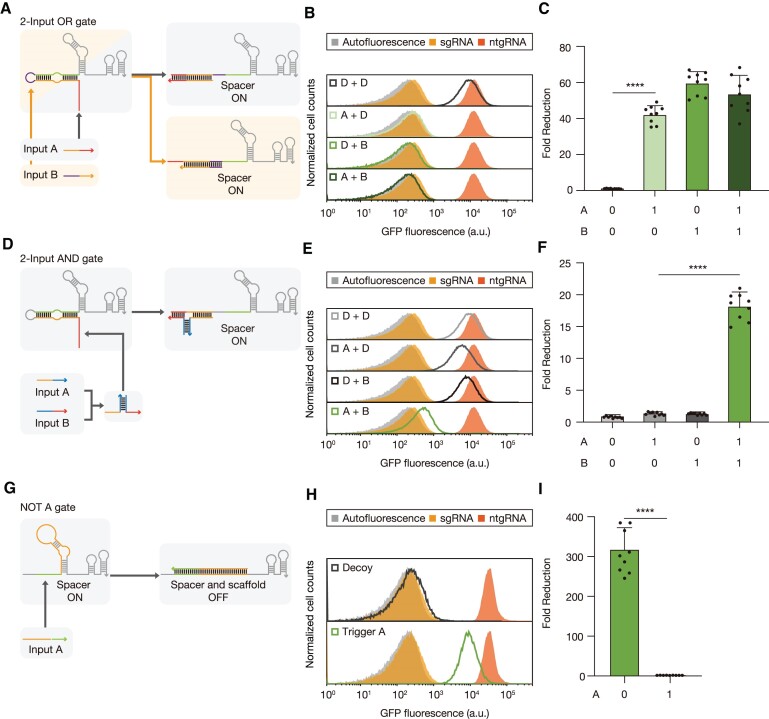
Logic gate implementation using cgRNA designs. (**A**) Two-input OR gate composed of a cgRNA and two triggers. Each input RNA initiates binding at the toehold domain or the loop domain, unwinding the spacer-sequestering structure to activate cgRNA. (**B**) Flow cytometry analysis of GFP fluorescence of cells expressing cgRNAs under the combination of trigger and/or decoy RNAs. (**C**) Fold reduction of fluorescence output for the two-input OR gate. (**D**) A two-input AND gate constructed from two input RNAs that bind to yield a complete trigger RNA complex. (**E**) Flow cytometry measurements of the two-input AND circuit under four combinations of input RNAs. (**F**) Fold reduction of the output for the two-input AND gate. (**G**) A NOT gate consists of a loop-extended cgRNA and a scaffold-complementary input RNA A. RNA input A binding to scaffold domain of cgRNA can disrupt the interaction of cgRNA and dCas9. (**H**) Flow cytometry analysis of the NOT gate with trigger and decoy RNA. (**I**) Fold reduction of the output for the NOT gate. GFP fold reduction was determined from the geometric mean fluorescence of cells measured via flow cytometry. Based on Welch's *t-*tests, ****P< 0.0001. The number of biological replicates is three, and the error bars for fold reductions are the s.d. of nine possible fold reductions for inactive and active cgRNA states.

Three different trigger design methods were employed to produce AND, OR and NOT gate circuits, which constitute a functionally complete set of Boolean logic operators (Supplementary Table S6). We designed two-input trigger RNAs for OR logic operation to initially bind either to the toehold region or the loop region of the cgRNA with 4-nt bulge (Figure 2A and Supplementary Figure S12). Upon either trigger binding, the spacer of the cgRNA is exposed by strand displacement and recognizes the target DNA, inhibiting the transcription of GFP.

CgRNA was expressed with different combinations of input RNAs A, B (toehold-binding and loop-binding, respectively), and non-cognate decoy RNAs to determine the logic capability for GFP regulation. The cognate inputs A and B robustly blocked GFP expression with repressed signal output in the presence of either one or both inputs, whereas the decoys yielded high fluorescence output, resulting in up to 60-fold reduction (Figure 2B, C and Supplementary Figure S13).

For an AND logic gate design, we split the trigger RNA into two, which have additional assembly domains to hybridize and form a complete trigger complex (17) (Figure 2D and Supplementary Figure S14). We adopted a hairpin structure at the 5′ end of both trigger RNAs, preventing undesired activation of cgRNA with only one trigger (Supplementary Figures S15 and S16). Trigger A stem interaction domain was shortened from 20-nt to 17-nt in order to function together with trigger B with reduced leakage (Supplementary Figures S17 and S18) (53). Measurements of GFP demonstrated about 18-fold reduction only when both trigger RNAs were expressed (Figure 2E, F).

The NOT logic gate behavior was achieved by RNA input A through hybridization of sgRNA scaffold, interrupting gRNA-Cas protein interaction (Figure 2G). We extended the loop region of the sgRNA scaffold to 20-nt such that the sgRNA scaffold could be efficiently targeted by the trigger RNA. Then, the trigger binding length was optimized to enable disruption of gRNA interaction with Cas protein (Supplementary Figures S19 and S20). GFP histograms showed a clear decrease in fluorescence in the absence of trigger A (Figure 2H, I).

**Figure 3.**
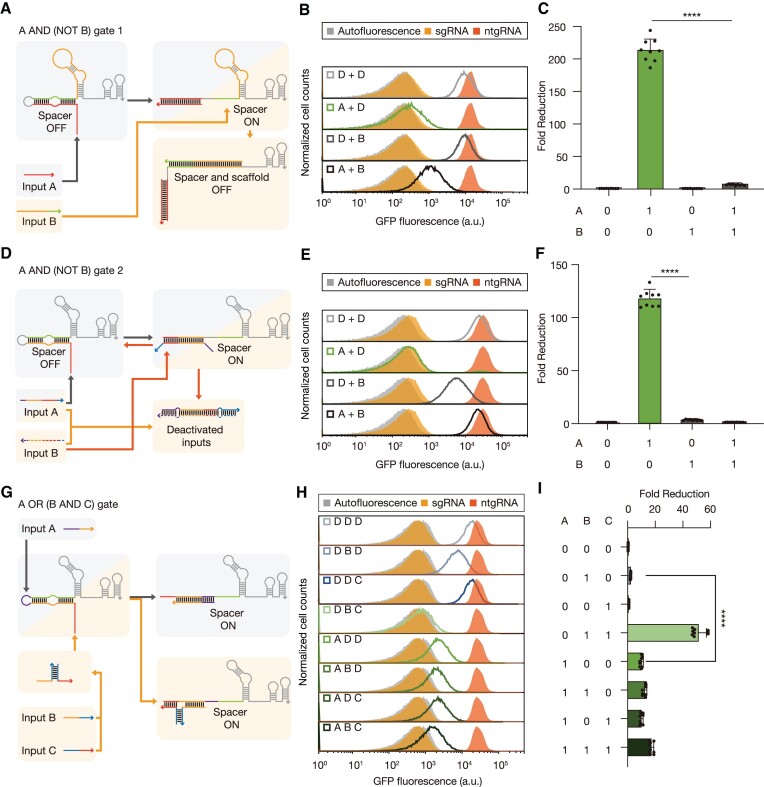
Implementation of complex biological logic circuits using OR, AND and NOT logic gates. (**A**, **D**) Two types of operating mechanism of the A AND (NOT B) circuit. For circuit 1, a deactivating RNA (input B) hybridizes to the scaffold domain of cgRNA (A). For circuit 2, input B directly binds to the trigger RNA A (D). (**B**, **E**) Flow cytometry analysis of the A AND (NOT B) circuit 1 (B) and 2 (E) under four combinations of input RNAs. (**C**, **F**) GFP fold reduction for the A AND (NOT B) circuit 1 (C) and 2 (F). (**G**) A three-input A OR (B AND C) logic gate composed of a cgRNA and three trigger RNAs. Input B and input C together form a complete trigger RNA analogous to the previous 2-input AND gate. (**H**) Flow cytometry analysis of the three-input A OR (B AND C) circuit under eight combinations of input RNAs. High GFP output for the three inactive cgRNA states and at least 10.2-fold reduction in GFP for the five active cgRNA states are shown. (**I**) The truth table and fold reduction for the A OR (B AND C) logic gate. Based on Welch's *t*-tests, ****P < 0.0001. The number of biological replicates is three, and the error bars for fold reductions are s.d. of nine possible fold reductions for inactive and active cgRNA states.

Next, we investigated whether the AND, OR and NOT logic gates could be combined to evaluate more sophisticated logic circuits. Two types of A AND (NOT B) gates were constructed: input A activates cgRNA for both designs, whereas input B binds to the extended 20-nt loop domain of cgRNA scaffold for design 1 or directly hybridizes to input A to inactivate cgRNA for design 2 (Figure 3A, D and Supplementary Figures S21 and S22). For the A AND (NOT B) gate-1 design, the fold-reduction was reduced from 215.1-fold for input A only to 9.4-fold for both inputs (Figure 3B, C). For the A AND (NOT B) gate-2 design, input B concentration was adjusted to be in excess of input A by using T7 promoter variants with different transcription strengths (54) (Supplementary Table S5 and Supplementary Figure S23). The promoter for antisense input B expression was chosen to be approximately five times the strength of the promoter for input A for effective inhibition. GFP expression was reduced by 118.5-fold in the presence of input A only, while a modest 1.1-fold reduction was observed when both inputs were expressed (Figure 3E, F).

Then, a three-input logic A OR (B AND C) gate was evaluated by a cgRNA (Figure 3G, Supplementary Figures S24, S25 and Supplementary Table S7). This circuit functioned robustly *in vivo*, displaying expected output of logic operation for all eight input combinations (Figure 3H, I). GFP fold reductions ranged from 10.2-fold to 51.6-fold when compared to the combination of three non-cognate inputs (0, 0, 0).

### E. *coli* cell growth control by regulation of endogenous genes

**Figure 4.**
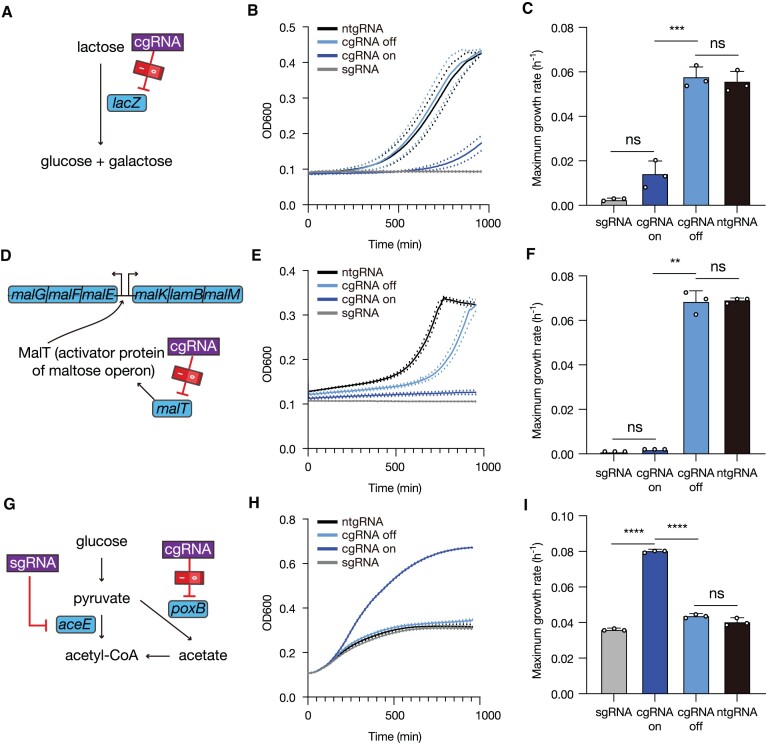
Regulation of endogenous gene expression (*lacZ*, *malT* and *poxB*) by cgRNAs. (**A**) Schematic of cgRNA-based *lacZ* regulation. (**B**) Growth curves of *E. coli* with lactose as the sole carbon source in M9 minimal media in the presence of active or inactive *lacZ* cgRNA or controls (ntgRNA and sgRNA). (**C**) Quantitative comparison of the maximum growth rate (h^−1^) under *lacZ* regulation. (**D**) Schematic of cgRNA-based *malT* regulation. (**E**) Growth curves of *E. coli* with maltose as the sole carbon source in M9 minimal media in the presence of active or inactive *malT* cgRNA or controls (ntgRNA and sgRNA). (**F**) Quantitative comparison of the maximum growth rate (h^−1^) under *malT* regulation. (**G**) Schematic of sgRNA and cgRNA combination for acetate metabolism. (**H**) Growth curves of *E. coli* with *aceE* sgRNA and active or inactive *poxB* cgRNA or controls (ntgRNA and sgRNA). (**I**) Quantitative comparison of the growth change with the knockdown of *aceE* and *poxB* based on the maximum growth rate (h^−1^). Based on Welch's *t-*tests, ns: not significant, **P < 0.01, ***P< 0.001 and ****P< 0.0001. The number of biological replicates is three.

To achieve signal-responsive metabolic and phenotypic control, endogenous genes should ideally be regulated in a programmable manner. We aimed to control the lactose operon, one of the most studied bacterial metabolic pathways, by knocking down the *lacZ* gene and inhibiting cell growth under conditions where lactose is the sole carbon source (Figure 4A). Analysis of the growth rate suggested that knockdown of the *lacZ* gene significantly repressed lactose metabolism (Figure 4B, C). The inactive cgRNA showed little inhibition of lactose metabolism, analogous to the ntgRNA, while the active cgRNA effectively repressed lactose metabolism (Supplementary Tables S8 and S9). We then targeted an alternative carbon source, maltose, to further explore the metabolic regulation potential of cgRNAs (Figure 4D). Conditional repression of *malT* suppressed cell growth in minimal media with maltose as the sole carbon source (Figure 4E, F). The maximal growth rate of cells with an off-state cgRNA was significantly increased with a slight delay in reaching maximal cell density compared to ntgRNA, whereas an active cgRNA allowed minimal growth of cells.

Encouraged by the robust regulation of metabolic pathways, we targeted metabolic flux regulation by cgRNAs for potential use in metabolic engineering. Engineering microorganisms for microbial cell factories requires maintaining a delicate balance between metabolic fluxes for different pathways, as they are interconnected and limited by the finite resources within the cell (55). The optimized cgRNA design with little leakages in its inactive state and a strong repression upon activation could be modulated by input RNA levels for balancing metabolic pathways. Flux control between acetate and other fermentation acid-producing pathways were selected as the target. A strong repression of *aceE* by sgRNA can lead to an acetate overflow and subsequent growth defects (Figure 4G) (56,57). On the other hand, a strong repression of both *aceE* and *poxB* by sgRNAs can also lead to growth defects due to deficient energy production.

We reasoned that a cgRNA-mediated knock-down of *poxB* to an intermediate level may balance the metabolic flux, recovering cell growth. The activated cgRNA targeting *poxB* showed an improved cell growth in minimal media with 0.4% (w/w) glucose (Figure 4H, I). In qPCR analysis, the expression level of the *poxB* gene with an active cgRNA was 6.46-fold higher than that of the sgRNA and 6.56-fold lower than that of the ntgRNA (Supplementary Figure S26).

### Dynamic morphology control using orthogonally regulated cgRNAs

**Figure 5.**
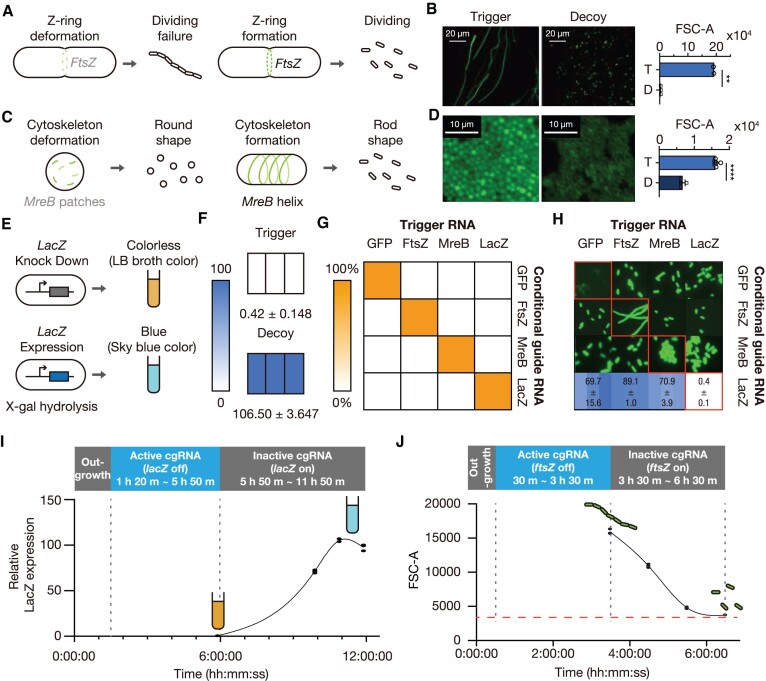
Regulation of endogenous gene expression (*ftsZ*, *mreB* and *lacZ*) by cgRNAs. (**A**) Expected phenotype in cgRNA-mediated *ftsZ* regulation. *ftsZ* repression by activated cgRNA disrupts Z-ring formation and causes filamentation. (**B**) Fluorescence images and flow cytometry analysis of cells expressing *ftsZ*-targeting cgRNA with trigger (left) or decoy (right) RNAs. Cell sizes are indicated with FSC-A values. (**C**) Expected phenotype in cgRNA-mediated *mreB* regulation. *mreB* repression disrupts cytoskeletal formation, increasing volume and diameter of a cell. (**D**) Fluorescence image and flow cytometry analysis of cells expressing *mreB*-targeting cgRNA with trigger (left) or decoy (right) RNAs. (**E**) Expected phenotypes in cgRNA-mediated *lacZ* regulation. The blue color indicates the enzymatic reaction of LacZ-mediated X-gal hydrolysis. (**F**) CgRNA was transcribed with trigger (above) or decoy (below) RNAs. The numbers represent the relative absorbance at OD 616 nm by scaling the X-gal treated WT cells (positive control) to a value of 100 and X-gal untreated WT cell (negative control) as a value of 0. (**G**) *In silico* evaluation of crosstalks among 16 combinations of 4 cgRNAs and their triggers used for gene regulation. The crosstalks were evaluated using NUPACK. (**H**) Cellular phenotypes from colonies of *E. coli* expressing 16 pairwise combinations of cgRNA and trigger RNAs. Phenotypic changes are visible along the diagonal where cells contain cognate cgRNA and trigger pairs. (**I**) Tracing cgRNA-mediated *lacZ* regulation through detection of β-galactosidase activity in *E. coli* after the removal of inducer for trigger RNA, L-arabinose. (**J**) Tracing cell morphology through flow cytometry analysis after the removal of inducer for trigger RNA. FSC-A value of wild-type *E. coli* is represented as a red dashed line. Based on Welch's *t-*tests, **P< 0.01 and ****P< 0.0001. The error bars are the s.d. of three biological replicates.

To exploit bacteria as sophisticated factories for effective production of inclusion bodies or hydrophobic natural products, a larger intracellular space and membrane area are needed (58–60). However, controlling genes related to morphology in *E. coli* poses a significant challenge as they are essential genes, imposing strong selective pressure. The sgRNA-based regulation approach requires multiple leak-control elements, without which substantial leakage makes the dynamic control of essential genes difficult (Figure 1E) (61). When sgRNA was used to regulate an essential gene such as *ftsZ*, the system was highly unstable and often resulted in loss-of-function (Supplementary Figure S27 and Supplementary Table S11) (62,63). The tight leakage control of cgRNA designs could be used for efficient regulation of essential genes. We designed two cgRNAs to target either an endogenous cell division gene *ftsZ* or a cytoskeletal gene *mreB* (Figure 5A, C). We tracked populations of elongated or enlarged cells by flow cytometry after induction and examined cell filamentation. The forward scatter (FSC-A) and GFP fluorescence of cells were dramatically increased, consistent with the images of *ftsZ* or *mreB* repressed cells (Figure 5B, D). The cell length distribution shifted toward greater lengths upon *ftsZ* knockdown. The proportion of elongated or enlarged cells after gene regulation was dramatically increased from 3.54% to 99.97% (*ftsZ*-regulated) and from 3.18% to 61.63% (*mreB*-regulated) (Supplementary Figure S28a, b, d, e). FSC-A levels of elongated or enlarged cells in the trigger-expressing group were consistent between trials (Supplementary Figure S28c, f). The functionality of *lacZ*-targeting cgRNA was further verified with X-gal assay that allows for a straightforward colorimetric assay of β-galactosidase activity (Figure 5E, F). To test the orthogonality of cgRNAs with respect to each other, we performed *in silico* evaluations and visualized pairwise interactions through fluorescence imaging or the X-gal assay in the cell lysate (Figure 5G, H). The set of four cgRNAs showed great performance with little crosstalk, indicating that cgRNAs could be applied to control multiple endogenous genes and metabolic pathways simultaneously.

Next, we tested the reversible control of endogenous *lacZ* by measuring β-galactosidase activity over time (Figure 5I). The cgRNA targeting *lacZ* was initially activated by trigger RNA expression and subsequently deactivated by halting the production of trigger RNA. The LacZ expression level was recovered to that of the positive control with constitutively expressed LacZ at 5 h after the removal of inducer to stop the trigger RNA expression. In addition, we targeted endogenous *ftsZ* to assess the reversibility of cell filamentation (Figure 5J). We observed a reduction in the proportion of filamented cells from 55.13% to 1.56% at 3 h after halting trigger RNA expression by inducer washout (Supplementary Figure S29). These results suggest that regulating metabolic pathways and cell morphology by cgRNA could be toggled on and off.

### Two-input logic operations for cell morphology control

High performance of cgRNAs form the basis of Boolean logic gate operations and endogenous gene regulation demonstrated so far. These two features can be combined to test the capability of regulating essential genes through logical evaluation of multiple inputs. When combined with sensing systems that detect environmental factors, such as temperature, osmotic pressure, electrons, and light (64–67), this novel signal processing unit for endogenous genes has promising implications for whole cell sensors as well as for in-situ cellular engineering (68).

**Figure 6.**
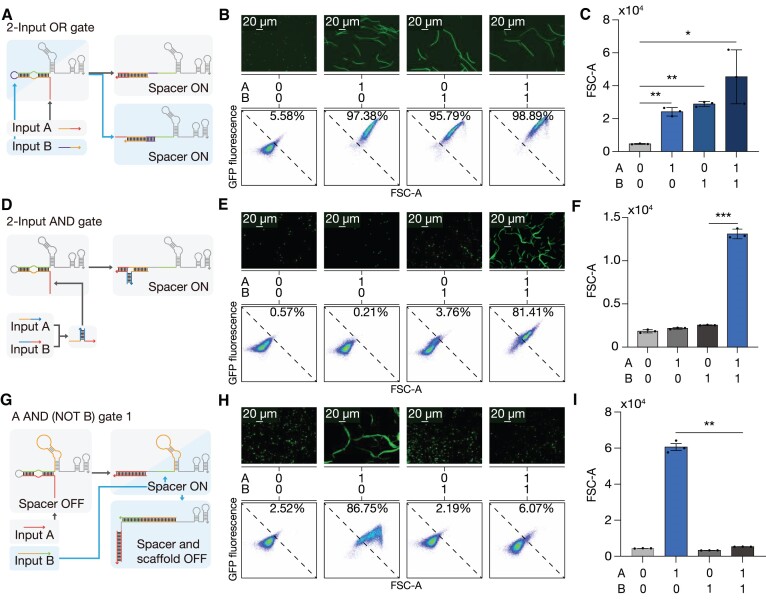
Two-input Boolean logic gates for regulation of *ftsZ*. Schematics of the (**A**) two-input OR, (**D**) two-input AND, and (**G**) A AND (NOT B) logic circuits. For (**B**, **E**, **H**), fluorescence images (above), truth tables and flow cytometry plots (below) of (B) two-input OR logic, (E) two-input AND logic and (H) A AND (NOT B) logic gate-mediated gene regulations of *ftsZ* via cgRNA. For (**C**, **F**, **I**), truth tables and histogram of FSC-A for 30 000 cells for (C) OR logic gate, (F) AND logic gate and (I) A AND (NOT B) logic gate. Based on Welch's *t-*tests, *P <0.05, **P <0.01, ***P <0.001. The error bars are the s.d. of three biological replicates.

We applied the Boolean logic gate design rationale to the *ftsZ*-targeting cgRNA and analyzed the phenotypic output by fluorescence imaging and flow cytometry. We first chose to implement an OR gate for cgRNA-mediated morphology modulation. Two triggers were designed to initiate hybridization from either the loop domain or the toehold domain, respectively. When either trigger A or B is present, the *ftsZ* cgRNA is activated, repressing *ftsZ* transcription, resulting in an elongated cell morphology (Figure 6A). Fluorescence microscopy and flow cytometry analysis showed a large proportion of elongated cells when either input was present (Figure 6B). The analysis of cell sizes by FSC-A indicated the *ftsZ* regulation of an OR-gate cgRNA by the combination of input RNAs as designed (Figure 6C).

Next, we chose to implement an AND gate for cgRNA-mediated morphology modulation. Two triggers were designed to hybridize and form a complete trigger RNA (Figure 6D). We observed a significant filamentation of cells only when both input RNAs were expressed (Figure 6E, F). The degree of filamentation was somewhat lower than those of the 2-input OR gate, possibly due to a lower efficiency in the assembly of two split triggers.

Then, we chose to implement an A AND (NOT B) gate for a cgRNA-mediated morphology modulation (Figure 6G). Analogous to the previous A AND (NOT B) gate 1 design (where cgRNA scaffold has the extended tetraloop), input RNA A was designed to activate the cgRNA while input RNA B was designed to disrupt the gRNA scaffold. Fluorescence microscopy and flow cytometry analysis indicated that a large portion of cells were elongated when only input A was expressed, while a low percentage of cells were elongated when both inputs were expressed (Figure 6H, I). These data suggest that the scaffold-complementary RNA-mediated NOT logic gate effectively disrupts CRISPRi-mediated cell filamentation. Together, the multi-input logic gate cgRNA reported here could be applied for the regulation of endogenous metabolism.

## Discussion

This work presents the RNA-responsive CRISPR/Cas regulatory element that enables the composition of multiple input RNA-processing logic gates using the CRISPR/Cas9 system. We optimized the cgRNAs by multiple design iterations guided by RNA design softwares and advanced computational tools. Next, we demonstrated two-input AND, OR, NOT logic gates and a three-input A OR (B AND C) logic operation. With the dynamic adjustability afforded by cgRNA designs, precise regulation of target gene expressions was demonstrated for enzymes, a transcription factor, and cytoskeleton associated proteins. Further, endogenous, metabolism-associated genes such as *lacZ* and *ftsZ* could be regulated in a reversible manner. The same design principles could be applied for logic-gated regulation of essential genes through cgRNAs.

To fully utilize the functionality of cgRNA designs presented here, there are certain design and operational considerations that need to be accounted for. First, the spacer-sequestering domain, especially the PAM-proximal seed, needs to form a very stable secondary structure with low ensemble defect. Second, incorporating a 4-nt bulge within the spacer-sequestering domain led to enhanced dynamic range by allowing complete activation by trigger RNA without increasing leakage in the absence of trigger. Third, optimizing the relative concentrations of dCas9, cgRNA and multiple trigger RNAs was critical for large dynamic ranges and precise logic computations. These factors collectively dictate the dynamic range, potential for leaky activation and the knockdown efficiency of activated cgRNAs.

Compared with previously reported gRNA-based CRISPR/Cas regulatory systems, the multi-input processing cgRNA designs presented here demonstrate several notable features. First, an optimized cgRNA design for transcription regulation features a high fold change and a tight control over leakage. Second, the cgRNA design can be programmed to construct multi-input logic processing circuits, potentially together with endogenous RNA inputs. Third, the precise regulation by cgRNAs allows for the control of metabolic pathways and cellular phenotypes. Finally, cgRNAs could modulate essential genes such as *ftsZ* by processing input RNAs with functionally complete set of two-input Boolean logic gates, resulting in dramatic morphological changes.

To further develop cgRNAs into a versatile complex signal processing platform, several improvements are desired. First, the partial sequence dependence to target and trigger needs to be addressed to sense endogenous RNA inputs, for instance, by using an AND-gate like architecture. Second, stringent requirements on structure switching capacity of cgRNAs necessitate careful consideration of spacer sequence compositions such as GC content, preferably together with gRNA screening software tools (14,69). Finally, the current thermodynamics-based design pipeline would greatly benefit from kinetic simulators, especially for multi-input logic processors (53,70–72).

Here, we constructed a set of logic gates and combined them for sophisticated metabolic manipulation. When combined with environmental signal-responsive promoters and orthogonal cgRNAs, this system could be applied for spatiotemporal regulation of multi-input processing live biotherapeutics (73). Previously reported cgRNA designs that function both in bacterial and mammalian cells indicate that certain cgRNA designs could be deployed in broad chassis organisms (74–76). Several notable differences in eukaryotic cells such as diffusive signal losses due to large volume, different mechanistic features in RNA degradation machinery, and the characteristic sequence requirements would pose challenges for porting the cgRNA devices (75,77). After further characterization, however, the cgRNA designs presented here could be applicable in mammalian systems for sophisticated cell fate engineering (78–80). Importantly, our cgRNA design strategy is likely to be compatible with several known Cas9-effector proteins, such as Cas9-based activators, genome or epigenome editors (25,81–84). In a preliminary test for general applicability of cgRNA designs, the cgRNA in combination with dCas9-activators showed 65.58-fold increase in output upon activation (Supplementary Table S12 and Supplementary Figure S30). The regulation of CRISPR/Cas system by cgRNA could be used to build DNA-RNP hybrid nanostructures for rational DNA folding and delivery of gene editing elements (85,86). Finally, cgRNA-mediated sophisticated regulation of RNA-protein, RNA-RNA and RNA-DNA assembly may extend the repertoire of regulatory elements for dynamic control of liquid nuclear condensates and synthetic metabolons (87–89).

## Supplementary Material

gkae549_Supplemental_Files

## Data Availability

The experimental data sets are either included in this submission, the supplemental information, or are available from the authors upon request.
