# Supplementary material for: Logical regulation of endogenous gene expression using programmable, multi-input processing CRISPR guide RNAs
Source: Nucleic Acids Res. 2024 Jun 29;52(14):8595–608. doi: 10.1093/nar/gkae549 (PMC11317168; doi:10.1093/nar/gkae549)
Supplement: gkae549_Supplemental_Files [file gkae549_supplemental_files.zip › NAR_Supplement_revised_final.pdf]

Supporting Information for

Logical Regulation of Endogenous Gene Expression using Programmable, Multi-input Processing CRISPR guide RNAs

Hansol Kang [1, †], Dongwon Park [1, †], Jongmin Kim[1, *]

[1] Department of Life Sciences, Pohang University of Science and Technology, Pohang 37673, Korea
* To whom correspondence should be addressed. Tel: +82-54-279-2322; Fax: 82-54-279-0666
Email: jongmin.kim@postech.ac.kr

† The authors wish it to be known that the first two authors should be regarded as Joint First Authors, and both have the right to place their name first.

Table of contents

1. Supplementary Materials and Methods -- 3
2. Supplementary Tables-- 9
    Table S1
    Table S2
    Table S3
    Table S4
    Table S5
    Table S6
    Table S7
    Table S8
    Table S9
    Table S10
    Table S11
    Table S12
    Table S13
3. Supplementary Figures-- 24
    Figure S1
    Figure S2
    Figure S3
    Figure S4
    Figure S5
    Figure S6
    Figure S7
    Figure S8
    Figure S9
    Figure S10
    Figure S11
    Figure S12
    Figure S13
    Figure S14
    Figure S15
    Figure S16
    Figure S17
    Figure S18
    Figure S19
    Figure S20
    Figure S21
    Figure S22
    Figure S23

Figure S24
Figure S25
Figure S26
Figure S27
Figure S28
Figure S29
Figure S30
4. References --- 59

Description of plasmids and conditional gRNA Device Evaluation

Conditional guide RNA

In all circuits, the conditional gRNA was expressed from the p15A plasmid using the pLtetO-1 promoter by anhydrotetracycline (aTc), terminated by the rrnB terminator and the T7Te terminator. dCas9 was located on the same plasmid and was transcribed from the pTet promoter by aTc, terminated by the rrnB T1 terminator.

OR, AND and NOT logic circuits

OR, AND, and NOT gate gRNA transcripts were expressed from a medium copy plasmid of p15A origin with chloramphenicol resistance. For two-input OR and AND gate systems, input RNAs, both cognate and non-cognate, were expressed from two high copy plasmids: a ColE1 origin plasmid with ampicillin resistance and a CDF origin plasmid with spectinomycin resistance.

For the implementation of the OR gate, expression cassettes were placed on plasmids with relative copy numbers (low for more favorable toehold binding trigger, low for less favorable loop binding trigger) to compensate for the activation efficiency of the operating method between each input.

For all AND circuits, all input conditions were tested using cells triple transformed with plasmids. At least one input RNA, either cognate or non-cognate, was expressed from each high copy plasmid for all truth table conditions tested for the circuits. All input RNAs were transcribed from the T7 promoter and have the first three GGG and T7 terminator sequences.

In the disjunctive normal form (DNF) circuit, two input RNAs were from one plasmid and the other input RNA was expressed from a separate plasmid. The initiator and repressor RNAs were expressed using the T7 promoter by l-arabinose. The three plasmids for the circuit were transformed into *E. coli* BL21-AI, which carries a chromosomal insertion of a cassette containing the T7 RNA polymerase (T7 RNAP) gene in the araB locus, allowing the expression of T7 RNAP to be regulated by the araBAD promoter. Expression of the inducer and repressor RNAs was induced with 0.2% (w/w) L-arabinose.

A AND (NOT B) Circuits

Type1: A AND (NOT B) circuits were tested using a three-plasmid system similar to that used for the OR and AND systems. The conditional gRNA was expressed from the chloramphenicol-resistant plasmid with a p15A origin of replication. The input trigger RNA was expressed from a spectinomycin-resistant plasmid

with a CDF origin. The inactivating RNA was expressed from an ampicillin-resistant plasmid with a ColE1 origin. Importantly, the plasmid copy number and promoter strength of the deactivator RNA plasmid was higher than that of the trigger to ensure that the deactivator RNAs (input B) could bind to all copies of the trigger RNA (input A) to enable successful circuit operation4. To create a difference in promoter strength, a weak variant of the T7 promoter was used to express the trigger RNA. The trigger and deactivator RNAs were expressed under the T7 promoter using L-arabinose. The three plasmids for the circuit were transformed into *E. coli* BL21(AI ™), which carries a chromosomal insertion of a cassette containing the T7 RNA polymerase (T7 RNAP) gene in the araB locus, allowing the expression of T7 RNAP to be regulated by the araBAD promoter. Expression of the inducer and repressor RNAs was induced with 0.2% (w/w) L-arabinose.

Design of Ribocomputing Device RNA sequences

Sample NUPACK code for design cgRNA (malT-targeting cgRNA and trigger design using online NUPACK)

trials = 10

material = rna1999

structure MalT = ... ((((((((....((((((((..........))))))))....)))))))) ....

domain toehold = N12

domain spacer = CGCCAGCTCAATGAAAAGCT

domain complementary = N20

domain loop = N10

domain handle =

GTTTTAGAGCTAGAAATAGCAAGTTAAAATAAGGCTAGTCCGTTATCAACTTGAAAAAGTGGCACCGAGTCGGTG CTTTTTT

prevent = AAAA, CCCC, GGGG, UUUU, MMMMMM, KKKKKK, WWWWWW, SSSSSS, RRRRRR, YYYYYY

MalT.seq = toehold complementary loop spacer handle

structure Tr = ...

domain terminator = TAGCATAACCCCTTGGGGCCTCTAAACGGGTCTTGAGGGGTTTTTT

Tr.seq = complementary* toehold* terminator

structure MalTTr = ((((((((((((((((((((((((((((((((.... +)))))))))))))))))))))))))))))))) ....

MalTTr.seq = toehold complementary loop spacer handle complementary* toehold* terminator

Sample python code for design cgRNA (gfp-targeting cgRNA and trigger design using python NUPACK library)

```
from nupack import *

config.parallelism = True

my_model = Model(material = 'rna', celsius = 37 )

dToehold = Domain('N12', name = 'dToehold')

dBulge = Domain('N4', name = 'dBulge')

dLoop = Domain('N10', name = 'dLoop')

dStemC = Domain('CATCTAAT', name = 'dStemC')

dBulge_RC = Domain('TCAA', name = 'dBulge_RC')

dStemD = Domain('CAAGAATT', name = 'dStemD')

dHandle = Domain('GTTTTAGAGCTAGAAATAGCAAGTTAAAATAAGGCTAGTCCG', name = 'dHandle')

dTr1 = Domain('GGGN32', name = 'dTr1')

sCgRNA = TargetStrand([dToehold, ~dStemD, dBulge, ~dStemC, dLoop, dStemC, dBulge_RC, dStemD,
dHandle], name = 'sCgRNA')

sTr1 = TargetStrand([dTr1], name = 'sTr1')

cCgRNA = TargetComplex([sCgRNA], '............((((((((.....(((((((..........))))))).....)))))))).(((((..((((....))))....)))))...((.....)).',
name = 'cCgRNA')

cTr1 = TargetComplex([sTr1], '...................................', name = 'cTr1')

cCgRNAxTr1 = TargetComplex([sTr1, sCgRNA],
'...((((((((((((((((((((((((((((((((+))))))))))))))))))))))))))))))))............................(((((..((((....))))....)))))...((.....)).', name = 'cCgR-
NAxTr1' )

reactants = TargetTube(on_targets=1, off_targets=SetSpec(max_size = 0), name = 'reactants')

products = TargetTube(on_targets={cTr1: 4e-09, cCgRNAxTr1: 1e-09}, off_targets=SetSpec(max_size =
0), name = 'products')
```

```
my_tubes = [reactants, products]
hard = [Diversity(word=4, types=2, scope=[dToehold, dBulge, dLoop]),
        Diversity(word=6, types=3, scope=[dToehold, dBulge, dLoop])]
my_design = tube_design(tubes=my_tubes, model=my_model, hard_constraints=hard)
my_jobs = my_design.launch(trials=1)
my_jobs.wait()
my_final_results = my_jobs.final_results()
print(my_final_results[0])
```

Conditional guide RNA Design

All cgRNAs were designed using online NUPACK or off-line NUPACK python package with AAAA, CCCC, GGGG, UUUU, KKKKKK, MMMMMM, RRRRRR, SSSSSS, WWWWWW, and YYYYYY as prevented sequences to avoid long strings or repeated nucleotides. All systems were designed using RNA free energy parameter set (1-5). The spacer sequence of guide RNA used a sequence verified in the previous literature (6). The cgRNAs consisted of a 12-nt toehold sequence, a 16-nt or 20-nt spacer hiding stem, and a 10-nt loop. Trigger RNA consists of a complementary sequence of 16nt or 20nt spacer hiding stem and 12-nt toehold sequence. For crosstalk minimization and stable target structure, the scaffold was included in the cgRNAs, and the trigger was designed with the first 3 Gs and T7 terminator included.

OR Gate Input Sequence Design

All OR input RNA sequences using the NUPACK Python package with AAAA, CCCC, GGGG, UUUU, KKKKKK, MMMMMM, RRRRRR, SSSSSS, WWWWWW, and YYYYYY as prevented sequences to avoid long strings or repeated nucleotides. All systems were designed using the RNA free energy parameter set (1). To support engineering efforts, complex ensemble defect was calculated to rely on equilibrium base-pairing properties of a dilute solution of interacting nucleic acid strands(a test tube) (1). For OR gate implementation, a common stem hybridization sequence and each strand displacement docking sequence were given, 12-nt toehold or 10-nt loop sequence of cgRNA. For crosstalk minimization and stable objective structure, inputs were designed including first 3 Gs and T7 terminator.

AND Gate Input Sequence Design

All AND input RNA sequences were designed NUPACK Python package with AAAA, CCCC, GGGG, UUUU, KKKKKK, MMMMMM, RRRRRR, SSSSSS, WWWWWW, and YYYYYY as prevented sequences to avoid long strings or repeated nucleotides. All systems were designed using the RNA free energy parameter set(1). To assist engineering efforts, complex ensemble defect was calculated to count on equilibrium base-pairing properties of a dilute solution of interacting nucleic acid strands (a test tube) (7). First, the 32-nt minimal trigger RNA sequence for a given conditional gRNA system was divided into toehold binding region and stem biding region. Then, in order to induce a topologically appropriate interaction between the isolated trigger sequence and cgRNA, structural flexibility was given by giving a 3-nt spacer sequence. Given the 21-nt trigger assembly domain, the two divided triggers were able to form one intact trigger through complementary RNA binding. In designing these AND-computing systems, we aimed to reduce the potential for leakage from expression of either of the AND gate half-trigger sequences. For crosstalk minimization and stable objective structure, inputs were designed including first 3 Gs and T7 terminator.

NOT Gate Input Sequence Design

All NOT input RNA sequences were designed NUPACK python package with AAAA, CCCC, GGGG, UUUU, KKKKKK, MMMMMM, RRRRRR, SSSSSS, WWWWWW, and YYYYYY as prevented sequences to avoid long strings or repeated nucleotides. All systems were designed using RNA free energy parameter set(1). To assist engineering efforts, complex ensemble defect was calculated to count on equilibrium base-pairing properties of a dilute solution of interacting nucleic acid strands (a test tube) (7). Trigger RNA is 15-nt 5' flanking region 1-nt spacer 32-nt trigger domain 1-nt spacer 15-nt 3' flanking region, and the deactivating trigger is a complementary sequence of 3' flanking region and trigger domain and 5' flanking region It is composed of, and as in the trigger, there is a 1-nt spacer domain between each region. The 1-nt spacer domain blocks the formation of continuous long dsRNA after the complementary bonding of the trigger-deactivating trigger, protecting the attack from endogenous RNase to test the reversible trigger RNA neutralization.

DNF Circuit Input Sequence Design

All DNF input RNA sequences were designed NUPACK python package with AAAA, CCCC, GGGG, UUUU, KKKKKK, MMMMMM, RRRRRR, SSSSSS, WWWWWW, and YYYYYY as prevented sequences to avoid long strings or repeated nucleotides. All systems were designed using RNA free energy parameter set(1). To assist engineering efforts, complex ensemble defect was calculated to count on equilibrium base-pairing

properties of a dilute solution of interacting nucleic acid strands (a test tube)(7). The DNF circuit was constructed by applying the design principle of the above-mentioned 2-input AND and OR gate.

Targeted next generation sequencing

We employed a specific primer pair (listed in **Table S10**) to amplify the expression cassette responsible for the expression of dCas9 and sgRNA. Subsequently, the amplified products underwent NGS analysis using a commercial service. The mutation ratio was assessed based on the average read count obtained from the analysis.

Supplementary Tables

Table S1. Plasmids used in this study. Abbreviations are as follows: T7term = T7 terminator, AmpR = ampicillin resistance gene, SpecR = spectinomycin resistance gene, KanR = kanamycin resistance gene, CmR = chloramphenicol resistance gene. All triggers, conditional gRNAs and target genes were cloned into pET15b AND/OR pCDFDuet, pACYCDuet, pSC101 plasmid.

Name	Sequence
Trigger	pT7 – Cognate trigger – T7term – AmpR - pBR322 origin - LacI
Conditional gRNA + dCas9	pLtetO-1 – Conditional gRNA – p15A ori – CmR – TetR – pTet – dcas9-ssrA (AAV)
Fluorescence reporter	pLlacO-1 – GFP-ssrA (ASV) – T7term – SC101 origin – KanR – LacI

Table S2. Examples of DNA plasmid sequences. Origin of replication and LacI has been reversed to other elements.

Name (architecture)	Sequence
Trigger (pT7 – Cognate Trigger – T7term – AmpR – pBR322 origin – LacI)	GTTGCGCGTTTGTCTGGTGTCTACGCCAGCGGAAATTAATACGACTCACTATAGGG<br>AACCGTAATTCTGATTCAAACGTAATGTAGCATAACCCCTTGGGGCCTCTAAACGG<br>GTCTTGAGGGGTTTTTTGCTGAAAGGAGGAACTATATCCGGATATCCCGCAAGAGG<br>CCCGGCAGTACCGGCATAACCAAGCCTATGCCTACAGCATCCAGGGTGACGGTGCC<br>GAGGATGACGATGAGCGCATTGTTAGATTTCATACACGGTGCCTGACTGCGTTAGC<br>AATTTAACTGTGATAAACTACCGCATTAAAGCTTATCGATGATAAGCTGTCAAACA<br>TGAGAATTCTTGAAGACGAAAGGGCCTCGTGATACGCCTATTTTTATAGGTTAATG<br>TCATGATAATAATGGTTTCTTAGACGTCAGGTGGCACTTTTCGGGGAAATGTGCGC<br>GGAACCCCTATTTGTTTATTTTTCTAAATACATTCAAATATGTATCCGCTCATGAG<br>ACAATAACCCTGATAAATGCTTCAATAATATTGAAAAAGGAAGAGTATGAGTATTC<br>AACATTTCCGTGTCGCCCTTATTCCCTTTTTTGCGGCATTTTGCCTTCCTGTTTTT<br>GCTCACCCAGAAACGCTGGTGAAAGTAAAAGATGCTGAAGATCAGTTGGGTGCACG<br>AGTGGGTTACATCGAACTGGATCTCAACAGCGGTAAGATCCTTGAGAGTTTTCGCC<br>CCGAAGAACGTTTTCCAATGATGAGCACTTTTAAAGTTCTGCTATGTGGCGCGGTA<br>TTATCCCGTGTTGACGCCGGGCAAGAGCAACTCGGTCGCCGCATACACTATTCTCA<br>GAATGACTTGGTTGAGTACTCACCAGTCACAGAAAAGCATCTTACGGATGGCATGA<br>CAGTAAGAGAATTATGCAGTGCTGCCATAACCATGAGTGATAACACTGCGGCCAAC<br>TTACTTCTGACAACGATCGGAGGACCGAAGGAGCTAACCGCTTTTTTGCACAACAT<br>GGGGGATCATGTAACTCGCCTTGATCGTTGGGAACCGGAGCTGAATGAAGCCATAC<br>CAAACGACGAGCGTGACACCACGATGCCTGCAGCAATGGCAACAACGTTGCGCAAA<br>CTATTAACTGGCGAACTACTTACTCTAGCTTCCCGGCAACAATTAATAGACTGGAT<br>GGAGGCGGATAAAGTTGCAGGACCACTTCTGCGCTCGGCCCTTCCGGCTGGCTGGT<br>TTATTGCTGATAAATCTGGAGCCGGTGAGCGTGGGTCTCGCGGTATCATTGCAGCA<br>CTGGGGCCAGATGGTAAGCCCTCCCGTATCGTAGTTATCTACACGACGGGGAGTCA<br>GGCAACTATGGATGAACGAAATAGACAGATCGCTGAGATAGGTGCCTCACTGATTA<br>AGCATTGGTAACTGTCAGACCAAGTTTACTCATATATACTTTAGATTGATTTAAAA<br>CTTCATTTTTAATTTAAAAGGATCTAGGTGAAGATCCTTTTTGATAATCTCATGAC<br>CAAAATCCCTTAACGTGAGTTTTCGTTCCACTGAGCGTCAGACCCCGTAGAAAAGA<br>TCAAAGGATCTTCTTGAGATCCTTTTTTTCTGCGCGTAATCTGCTGCTTGCAAACA<br>AAAAAACCACCGCTACCAGCGGTGGTTTGTTTGCCGGATCAAGAGCTACCAACTCT

	TTTTCCGAAGGTAACTGGCTTCAGCAGAGCGCAGATACCAAATACTGTCCTTCTAG<br>TGTAGCCGTAGTTAGGCCACCACTTCAAGAACTCTGTAGCACCGCCTACATACCTC<br>GCTCTGCTAATCCTGTTACCAGTGGCTGCTGCCAGTGGCGATAAGTCGTGTCTTAC<br>CGGGTTGGACTCAAGACGATAGTTACCGGATAAGGCGCAGCGGTCGGGCTGAACGG<br>GGGGTTCGTGCACACAGCCCAGCTTGGAGCGAACGACCTACACCGAACTGAGATAC<br>CTACAGCGTGAGCTATGAGAAAGCGCCACGCTTCCCGAAGGGAGAAAGGCGGACAG<br>GTATCCGGTAAGCGGCAGGGTCGGAACAGGAGAGCGCACGAGGGAGCTTCCAGGGG<br>GAAACGCCTGGTATCTTTATAGTCCTGTCGGGTTTCGCCACCTCTGACTTGAGCGT<br>CGATTTTTGTGATGCTCGTCAGGGGGGCGGAGCCTATGGAAAAACGCCAGCAACGC<br>GGCCTTTTTACGGTTCCTGGCCTTTTGCTGGCCTTTTGCTCACATGTTCTTTCCTG<br>CGTTATCCCCTGATTCTGTGGATAACCGTATTACCGCCTTTGAGTGAGCTGATACC<br>GCTCGCCGCAGCCGAACGACCGAGCGCAGCGAGTCAGTGAGCGAGGAAGCGGAAGA<br>GCGCCTGATGCGGTATTTTCTCCTTACGCATCTGTGCGGTATTTCACACCGCATAT<br>ATGGTGCACTCTCAGTACAATCTGCTCTGATGCCGCATAGTTAAGCCAGTATACAC<br>TCCGCTATCGCTACGTGACTGGGTCATGGCTGCGCCCCGACACCCGCCAACACCCG<br>CTGACGCGCCCTGACGGGCTTGTCTGCTCCCGGCATCCGCTTACAGACAAGCTGTG<br>ACCGTCTCCGGGAGCTGCATGTGTCAGAGGTTTTCACCGTCATCACCGAAACGCGC<br>GAGGCAGCTGCGGTAAAGCTCATCAGCGTGGTCGTGAAGCGATTCACAGATGTCTG<br>CCTGTTCATCCGCGTCCAGCTCGTTGAGTTTCTCCAGAAGCGTTAATGTCTGGCTT<br>CTGATAAAGCGGGCCATGTTAAGGGCGGTTTTTTCCTGTTTGGTCACTGATGCCTC<br>CGTGTAAGGGGGATTTCTGTTCATGGGGGTAATGATACCGATGAAACGAGAGAGGA<br>TGCTCACGATACGGGTTACTGATGATGAACATGCCCGGTTACTGGAACGTTGTGAG<br>GGTAAACAACTGGCGGTATGGATGCGGCGGGACCAGAGAAAAATCACTCAGGGTCA<br>ATGCCAGCGCTTCGTTAATACAGATGTAGGTGTTCCACAGGGTAGCCAGCAGCATC<br>CTGCGATGCAGATCCGGAACATAATGGTGCAGGGCGCTGACTTCCGCGTTTCCAGA<br>CTTTACGAAACACGGAAACCGAAGACCATTCATGTTGTTGCTCAGGTCGCAGACGT<br>TTTGCAGCAGCAGTCGCTTCACGTTCGCTCGCGTATCGGTGATTCATTCTGCTAAC<br>CAGTAAGGCAACCCCGCCAGCCTAGCCGGGTCCTCAACGACAGGAGCACGATCATG<br>CGCACCCGTGGCCAGGACCCAACGCTGCCCGAGATGCGCCGCGTGCGGCTGCTGGA<br>GATGGCGGACGCGATGGATATGTTCTGCCAAGGGTTGGTTTGCGCATTCACAGTTC<br>TCCGCAAGAATTGATTGGCTCCAATTCTTGGAGTGGTGAATCCGTTAGCGAGGTGC<br>CGCCGGCTTCCATTCAGGTCGAGGTGGCCCGGCTCCATGCACCGCGACGCAACGCG<br>GGGAGGCAGACAAGGTATAGGGCGGCGCCTACAATCCATGCCAACCCGTTCCATGT<br>GCTCGCCGAGGCGGCATAAATCGCCGTGACGATCAGCGGTCCAGTGATCGAAGTTA<br>GGCTGGTAAGAGCCGCGAGCGATCCTTGAAGCTGTCCCTGATGGTCGTCATCTACC<br>TGCCTGGACAGCATGGCCTGCAACGCGGGCATCCCGATGCCGCCGGAAGCGAGAAG<br>AATCATAATGGGGAAGGCCATCCAGCCTCGCGTCGCGAACGCCAGCAAGACGTAGC<br>CCAGCGCGTCGGCCGCCATGCCGGCGATAATGGCCTGCTTCTCGCCGAAACGTTTG<br>GTGGCGGGACCAGTGACGAAGGCTTGAGCGAGGGCGTGCAAGATTCCGAATACCGC<br>AAGCGACAGGCCGATCATCGTCGCGCTCCAGCGAAAGCGGTCCTCGCCGAAAATGA<br>CCCAGAGCGCTGCCGGCACCTGTCCTACGAGTTGCATGATAAAGAAGACAGTCATA<br>AGTGCGGCGACGATAGTCATGCCCCGCGCCCACCGGAAGGAGCTGACTGGGTTGAA<br>GGCTCTCAAGGGCATCGGTCGAGATCCCGGTGCCTAATGAGTGAGCTAACTTACAT<br>TAATTGCGTTGCGCTCACTGCCCGCTTTCCAGTCGGGAAACCTGTCGTGCCAGCTG<br>CATTAATGAATCGGCCAACGCGCGGGGAGAGGCGGTTTGCGTATTGGGCGCCAGGG<br>TGGTTTTTCTTTTCACCAGTGAGACGGGCAACAGCTGATTGCCCTTCACCGCCTGG<br>CCCTGAGAGAGTTGCAGCAAGCGGTCCACGCTGGTTTGCCCCAGCAGGCGAAAATC<br>CTGTTTGATGGTGGTTAACGGCGGGATATAACATGAGCTGTCTTCGGTATCGTCGT<br>ATCCCACTACCGAGATATCCGCACCAACGCGCAGCCCGGACTCGGTAATGGCGCGC<br>ATTGCGCCCAGCGCCATCTGATCGTTGGCAACCAGCATCGCAGTGGGAACGATGCC<br>CTCATTCAGCATTTGCATGGTTTGTTGAAAACCGGACATGGCACTCCAGTCGCCTT<br>CCCGTTCCGCTATCGGCTGAATTTGATTGCGAGTGAGATATTTATGCCAGCCAGCC<br>AGACGCAGACGCGCCGAGACAGAACTTAATGGGCCCGCTAACAGCGCGATTTGCTG<br>GTGACCCAATGCGACCAGATGCTCCACGCCCAGTCGCGTACCGTCTTCATGGAGA

	AAATAATACTGTTGATGGGTGTCTGGTCAGAGACATCAAGAAATAACGCCGGAACA<br>TTAGTGCAGGCAGCTTCCACAGCAATGGCATCCTGGTCATCCAGCGGATAGTTAAT<br>GATCAGCCCACTGACGCGTTGCGCGAGAAGATTGTGCACCGCCGCTTTACAGGCTT<br>CGACGCCGCTTCGTTCTACCATCGACACCACCACGCTGGCACCCAGTTGATCGGCG<br>CGAGATTTAATCGCCGCGACAATTTGCGACGGCGCGTGCAGGGCCAGACTGGAGGT<br>GGCAACGCCAATCAGCAACGACTGTTTGCCCGCCAGTTGTTGTGCCACGCGGTTGG<br>GAATGTAATTCAGCTCCGCCATCGCCGCTTCCACTTTTTCCCGCGTTTTCGCAGAA<br>ACGTGGCTGGCCTGGTTCACCACGCGGGAAACGGTCTGATAAGAGACACCGGCATA<br>CTCTGCGACATCGTATAACGTTACTGGTTTCACATTCACCACCCTGAATTGACTCT<br>CTTCCGGGCGCTATCATGCCATACCGCGAAAGGTTTTGCGCCATTCGATGGTGTCC<br>GGGATCTCGACGCTCTCCCTTATGCGACTCCTGCATTAGGAAGCAGCCCAGTAGTA<br>GGTTGAGGCCGTTGAGCACCGCCGCCGCAAGGAATGGTGCATGCAAGGAGATGGCG<br>CCCAACAGTCCCCCGGCCACGGGGCCTGCCACCATACCCACGCCGAAACAAGCGCT<br>CATGAGCCCGAAGTGGCGAGCCCGATCTTCCCCATCGGTGATGTCGGCGATATAGG<br>CGCCAGCAACCGCACCTGTGGCGCCGGTGATGCCGGCCACGATGCGTCCGGCGTAG<br>AGGATCGAGATCTCGAACGTGTACGGGCTATCTGGCTTTC
Conditional gRNA (pLtetO-1 – Conditional gRNA – p15A ori – CmR – TetR – dCas9-ssrA (ASV))	CAGCCAGGATCCGAATTCGAGCTCACCAATCCCTATCAGTGATAGAGATTGACATC<br>CCTATCAGTGATAGATATACTGAGCACCTATCTAATCTGCTTGTTGAATTAGGTGC<br>CGCGGAATACATCTAATTCAACAAGAATTGTTTTAGAGCTAGAAATAGCAAGTTAA<br>AATAAGGCTAGTCCGTTATCAACTTGAAAAAGTGGCACCGAGTCGGTGCTTTTTTT<br>CGCGGCTCACCTTCGGGTGGGCCTTTCTGCGTTTATACCTAGGGATATATTCCGCT<br>TCCTCGCTCACTGACTCGCTACGCTCGGTCGTTCGACTGCGGCGAGCGGAAATGGC<br>TTACGAACGGGGCGGAGATTTCCTGGAAGATGCCAGGAAGATACTTAACAGGGAAG<br>TGAGAGGGCCGCGGCAAAGCCGTTTTTCCATAGGCTCCGCCCCCCTGACAAGCATC<br>ACGAAATCTGACGCTCAAATCAGTGGTGGCGAAACCCGACAGGACTATAAAGATAC<br>CAGGCGTTTCCCCCTGGCGGCTCCCTCGTGCGCTCTCCTGTTCCTGCCTTTCGGTT<br>TACCGGTGTCATTCCGCTGTTATGGCCGCGTTTGTCTCATTCCACGCCTGACACTC<br>AGTTCCGGGTAGGCAGTTCGCTCCAAGCTGGACTGTATGCACGAACCCCCCGTTCA<br>GTCCGACCGCTGCGCCTTATCCGGTAACTATCGTCTTGAGTCCAACCCGGAAAGAC<br>ATGCAAAAGCACCACTGGCAGCAGCCACTGGTAATTGATTTAGAGGAGTTAGTCTT<br>GAAGTCATGCGCCGGTTAAGGCTAAACTGAAAGGACAAGTTTTGGTGACTGCGCTC<br>CTCCAAGCCAGTTACCTCGGTTCAAAGAGTTGGTAGCTCAGAGAACCTTCGAAAAA<br>CCGCCCTGCAAGGCGGTTTTTTCGTTTTCAGAGCAAGAGATTACGCGCAGACCAAA<br>ACGATCTCAAGAAGATCATCTTATTAATCAGATAAAATATTTCTAGATTTCAGTGC<br>AATTTATCTCTTCAAATGTAGCACCTGAAGTCAGCCCCATACGATATAAGTTGTTA<br>CTAGTGCTTGGATTCTCACCAATAAAAAACGCCCGGCGGCAACCGAGCGTTCTGAA<br>CAAATCCAGATGGAGTTCTGAGGTCATTACTGGATCTATCAACAGGAGTCCAAGCG<br>AGCTCGATATCAAATTACGCCCCGCCCTGCCACTCATCGCAGTACTGTTGTAATTC<br>ATTAAGCATTCTGCCGACATGGAAGCCATCACAAACGGCATGATGAACCTGAATCG<br>CCAGCGGCATCAGCACCTTGTCGCCTTGCGTATAATATTTGCCCATGGTGAAAACG<br>GGGGCGAAGAAGTTGTCCATATTGGCCACGTTTAAATCAAAACTGGTGAAACTCAC<br>CCAGGGATTGGCTGAGACGAAAAACATATTCTCAATAAACCCTTTAGGGAAATAGG<br>CCAGGTTTTCACCGTAACACGCCACATCTTGCGAATATATGTGTAGAAACTGCCGG<br>AAATCGTCGTGGTATTCACTCCAGAGCGATGAAAACGTTTCAGTTTGCTCATGGAA<br>AACGGTGTAACAAGGGTGAACACTATCCCATATCACCAGCTCACCGTCTTTCATTG<br>CCATACGAAATTCCGGATGAGCATTCATCAGGCGGGCAAGAATGTGAATAAAGGCC<br>GGATAAAACTTGTGCTTATTTTTCTTTACGGTCTTTAAAAAGGCCGTAATATCCAG<br>CTGAACGGTCTGGTTATAGGTACATTGAGCAACTGACTGAAATGCCTCAAAATGTT<br>CTTTACGATGCCATTGGGATATATCAACGGTGGTATATCCAGTGATTTTTTTCTCC<br>ATTTTAGCTTCCTTAGCTCCTGAAAATCTCGATAACTCAAAAAATACGCCCGGTAG<br>TGATCTTATTTCATTATGGTGAAAGTTGGAACCTCTTACGTGCCGATCAACGTCTC<br>ATTTTCGCCAGATATCGACGTCTTAAGACCCACTTTCACATTTAAGTTGTTTTTCT<br>AATCCGCATATGATCAATTCAAGGCCGAATAAGAAGGCTGGCTCTGCACCTTGGTG

	ATCAAATAATTCGATAGCTTGTCGTAATAATGGCGGCATACTATCAGTAGTAGGTG<br>TTTCCCTTTCTTCTTTAGCGACTTGATGCTCTTGATCTTCCAATACGCAACCTAAA<br>GTAAAATGCCCCACAGCGCTGAGTGCATATAATGCATTCTCTAGTGAAAAACCTTG<br>TTGGCATAAAAAGGCTAATTGATTTTCGAGAGTTTCATACTGTTTTTCTGTAGGCC<br>GTGTACCTAAATGTACTTTTGCTCCATCGCGATGACTTAGTAAAGCACATCTAAAA<br>CTTTTAGCGTTATTACGTAAAAAATCTTGCCAGCTTTCCCCTTCTAAAGGGCAAAA<br>GTGAGTATGGTGCCTATCTAACATCTCAATGGCTAAGGCGTCGAGCAAAGCCCGCT<br>TATTTTTTACATGCCAATACAATGTAGGCTGCTCTACACCTAGCTTCTGGGCGAGT<br>TTACGGGTTGTTAAACCTTCGATTCCGACCTCATTAAGCAGCTCTAATGCGCTGTT<br>AATCACTTTACTTTTATCTAATCTAGACATCATTAATTCCTAATTTTTGTTGACAC<br>TCTATCGTTGATAGAGTTATTTTACCACTCCCTATCAGTGATAGAGAAAAGAATTC<br>AAAAGATCTAAAGAGGAGAAAGGATCTATGGATAAGAAATACTCAATAGGCTTAGC<br>TATCGGCACAAATAGCGTCGGATGGGCGGTGATCACTGATGAATATAAGGTTCCGT<br>CTAAAAAGTTCAAGGTTCTGGGAAATACAGACCGCCACAGTATCAAAAAAAATCTT<br>ATAGGGGCTCTTTTATTTGACAGTGGAGAGACAGCGGAAGCGACTCGTCTCAAACG<br>GACAGCTCGTAGAAGGTATACACGTCGGAAGAATCGTATTTGTTATCTACAGGAGA<br>TTTTTTCAAATGAGATGGCGAAAGTAGATGATAGTTTCTTTCATCGACTTGAAGAG<br>TCTTTTTTGGTGGAAGAAGACAAGAAGCATGAACGTCATCCTATTTTTGGAAATAT<br>AGTAGATGAAGTTGCTTATCATGAGAAATATCCAACTATCTATCATCTGCGAAAAA<br>AATTGGTAGATTCTACTGATAAAGCGGATTTGCGCTTAATCTATTTGGCCTTAGCG<br>CATATGATTAAGTTTCGTGGTCATTTTTTGATTGAGGGAGATTTAAATCCTGATAA<br>TAGTGATGTGGACAAACTATTTATCCAGTTGGTACAAACCTACAATCAATTATTTG<br>AAGAAAACCCTATTAACGCAAGTGGAGTAGATGCTAAAGCGATTCTTTCTGCACGA<br>TTGAGTAAATCAAGACGATTAGAAAATCTCATTGCTCAGCTCCCCGGTGAGAAGAA<br>AAATGGCTTATTTGGGAATCTCATTGCTTTGTCATTGGGTTTGACCCCTAATTTTA<br>AATCAAATTTTGATTTGGCAGAAGATGCTAAATTACAGCTTTCAAAAGATACTTAC<br>GATGATGATTTAGATAATTTATTGGCGCAAATTGGAGATCAATATGCTGATTTGTT<br>TTTGGCAGCTAAGAATTTATCAGATGCTATTTTACTTTCAGATATCCTAAGAGTAA<br>ATACTGAAATAACTAAGGCTCCCCTATCAGCTTCAATGATTAAACGCTACGATGAA<br>CATCATCAAGACTTGACTCTTTTAAAAGCTTTAGTTCGACAACAACTTCCAGAAAA<br>GTATAAAGAAATCTTTTTTGATCAATCAAAAAACGGATATGCAGGTTATATTGATG<br>GGGGAGCTAGCCAAGAAGAATTTTATAAATTTATCAAACCAATTTTAGAAAAAATG<br>GATGGTACTGAGGAATTATTGGTGAAACTAAATCGTGAAGATTTGCTGCGCAAGCA<br>ACGGACCTTTGACAACGGCTCTATTCCCCATCAAATTCACTTGGGTGAGCTGCATG<br>CTATTTTGAGAAGACAAGAAGACTTTTATCCATTTTTAAAAGACAATCGTGAGAAG<br>ATTGAAAAAATCTTGACTTTTCGAATTCCTTATTATGTTGGTCCATTGGCGCGTGG<br>CAATAGTCGTTTTGCATGGATGACTCGGAAGTCTGAAGAAACAATTACCCCATGGA<br>ATTTTGAAGAAGTTGTCGATAAAGGTGCTTCAGCTCAATCATTTATTGAACGCATG<br>ACAAACTTTGATAAAAATCTTCCAAATGAAAAAGTACTACCAAAACATAGTTTGCT<br>TTATGAGTATTTTACGGTTTATAACGAATTGACAAAGGTCAAATATGTTACTGAAG<br>GAATGCGAAAACCAGCATTTCTTTCAGGTGAACAGAAGAAAGCCATTGTTGATTTA<br>CTCTTCAAAACAAATCGAAAAGTAACCGTTAAGCAATTAAAAGAAGATTATTTCAA<br>AAAAATAGAATGTTTTGATAGTGTTGAAATTTCAGGAGTTGAAGATAGATTTAATG<br>CTTCATTAGGTACCTACCATGATTTGCTAAAAATTATTAAAGATAAAGATTTTTTG<br>GATAATGAAGAAAATGAAGATATCTTAGAGGATATTGTTTTAACATTGACCTTATT<br>TGAAGATAGGGAGATGATTGAGGAAAGACTTAAAACATATGCTCACCTCTTTGATG<br>ATAAGGTGATGAAACAGCTTAAACGTCGCCGTTATACTGGTTGGGGACGTTTGTCT<br>CGAAAATTGATTAATGGTATTAGGGATAAGCAATCTGGCAAAACAATATTAGATTT<br>TTTGAAATCAGATGGTTTTGCCAATCGCAATTTTATGCAGCTGATCCATGATGATA<br>GTTTGACATTTAAAGAAGACATTCAAAAAGCACAAGTGTCTGGACAAGGCGATAGT<br>TTACATGAACATATTGCAAATTTAGCTGGTAGCCCTGCTATTAAAAAAGGTATTTT<br>ACAGACTGTAAAAGTTGTTGATGAATTGGTCAAAGTAATGGGGCGGCATAAGCCAG<br>AAAATATCGTTATTGAAATGGCACGTGAAAATCAGACAACTCAAAAGGGCCAGAAA<br>AATTCGCGAGAGCGTATGAAACGAATCGAAGAAGGTATCAAAGAATTAGGAAGTCA

	GATTCTTAAAGAGCATCCTGTTGAAAATACTCAATTGCAAAATGAAAAGCTCTATC<br>TCTATTATCTCCAAAATGGAAGAGACATGTATGTGGACCAAGAATTAGATATTAAT<br>CGTTTAAGTGATTATGATGTCGATGCCATTGTTCCACAAAGTTTCCTTAAAGACGA<br>TTCAATAGACAATAAGGTCTTAACGCGTTCTGATAAAAATCGTGGTAAATCGGATA<br>ACGTTCCAAGTGAAGAAGTAGTCAAAAAGATGAAAAACTATTGGAGACAACTTCTA<br>AACGCCAAGTTAATCACTCAACGTAAGTTTGATAATTTAACGAAAGCTGAACGTGG<br>AGGTTTGAGTGAACTTGATAAAGCTGGTTTTATCAAACGCCAATTGGTTGAAACTC<br>GCCAAATCACTAAGCATGTGGCACAAATTTTGGATAGTCGCATGAATACTAAATAC<br>GATGAAAATGATAAACTTATTCGAGAGGTTAAAGTGATTACCTTAAAATCTAAATT<br>AGTTTCTGACTTCCGAAAAGATTTCCAATTCTATAAAGTACGTGAGATTAACAATT<br>ACCATCATGCCCATGATGCGTATCTAAATGCCGTCGTTGGAACTGCTTTGATTAAG<br>AAATATCCAAAACTTGAATCGGAGTTTGTCTATGGTGATTATAAAGTTTATGATGT<br>TCGTAAAATGATTGCTAAGTCTGAGCAAGAAATAGGCAAAGCAACCGCAAAATATT<br>TCTTTTACTCTAATATCATGAACTTCTTCAAAACAGAAATTACACTTGCAAATGGA<br>GAGATTCGCAAACGCCCTCTAATCGAAACTAATGGGGAAACTGGAGAAATTGTCTG<br>GGATAAAGGGCGAGATTTTGCCACAGTGCGCAAAGTATTGTCCATGCCCCAAGTCA<br>ATATTGTCAAGAAAACAGAAGTACAGACAGGCGGATTCTCCAAGGAGTCAATTTTA<br>CCAAAAAGAAATTCGGACAAGCTTATTGCTCGTAAAAAAGACTGGGATCCAAAAAA<br>ATATGGTGGTTTTGATAGTCCAACGGTAGCTTATTCAGTCCTAGTGGTTGCTAAGG<br>TGGAAAAAGGGAAATCGAAGAAGTTAAAATCCGTTAAAGAGTTACTAGGGATCACA<br>ATTATGGAAAGAAGTTCCTTTGAAAAAAATCCGATTGACTTTTTAGAAGCTAAAGG<br>ATATAAGGAAGTTAAAAAAGACTTAATCATTAAACTACCTAAATATAGTCTTTTTG<br>AGTTAGAAAACGGTCGTAAACGGATGCTGGCTAGTGCCGGAGAATTACAAAAAGGA<br>AATGAGCTGGCTCTGCCAAGCAAATATGTGAATTTTTTATATTTAGCTAGTCATTA<br>TGAAAAGTTGAAGGGTAGTCCAGAAGATAACGAACAAAAACAATTGTTTGTGGAGC<br>AGCATAAGCATTATTTAGATGAGATTATTGAGCAAATCAGTGAATTTTCTAAGCGT<br>GTTATTTTA0GCAGATGCCAATTTAGATAAAGTTCTTAGTGCATATAACAAACATA<br>GAGACAAACCAATACGTGAACAAGCAGAAAATATTATTCATTTATTTACGTTGACG<br>AATCTTGGAGCTCCCGCTGCTTTTAAATATTTTGATACAACAATTGATCGTAAACG<br>ATATACGTCTACAAAAGAAGTTTTAGATGCCACTCTTATCCATCAATCCATCACTG<br>GTCTTTATGAAACACGCATTGATTTGAGTCAGCTAGGAGGTGACAGGCCTGCAGCA<br>AACGACGAAAACTACGCTGCAGCAGTTTAACTCGAGTAAGGATCTCCAGGCATCAA<br>ATAAAACGAAAGGCTCAGTCGAAAGACTGGGCCTTTCGTTTTATCTGTTGTTTGTC<br>GGTGAACGCTCTCTACTAGAATCA
Target Gene (pLlacO-1 – RBS – GFP-ssrA (ASV) – T7term – SC101 origin – KanR – LacI)	ATAAATGTGAGCGGATAACATTGACATTGTGAGCGGATAACAAGATACTGAGCACG<br>AAAGTGAAAGTAAGAATAAACAGAGGAGAATAAAGATGAAAGAGACGATGCGTAAA<br>GGAGAAGAACTTTTCACTGGAGTTGTCCCAATTCTTGTTGAATTAGATGGTGATGT<br>TAATGGGCACAAATTTTCTGTCAGTGGAGAGGGTGAAGGTGATGCAACATACGGAA<br>AACTTACCCTTAAATTTATTTGCACTACTGGAAAACTACCTGTTCCGTGGCCAACA<br>CTTGTCACTACTTTCGGTTATGGTGTTCAATGCTTTGCGAGATACCCAGATCACAT<br>GAAACAGCATGACTTTTTCAAGAGTGCCATGCCCGAAGGTTACGTACAGGAAAGAA<br>CTATATTTTTCAAAGATGACGGGAACTACAAGACACGTGCTGAAGTCAAGTTTGAA<br>GGTGATACCCTTGTTAATAGAATCGAGTTAAAAGGTATTGATTTTAAAGAAGATGG<br>AAACATTCTTGGACACAAATTGGAATACAACTATAACTCACACAATGTATACATCA<br>TGGCAGACAAACAAAAGAATGGAATCAAAGTTAACTTCAAAATTAGACACAACATT<br>GAAGATGGAAGCGTTCAACTAGCAGACCATTATCAACAAAATACTCCGATTGGCGA<br>TGGCCCTGTCCTTTTACCAGACAACCATTACCTGTCCACACAATCTGCCCTTTCGA<br>AAGATCCCAACGAAAAGAGAGACCACATGGTCCTTCTTGAGTTTGTAACCGCTGCT<br>GGGATTACACATGGCATGGATGAACTATACAAAAGGCCTGCAGCAAACGACGAAAA<br>CTACGCTGCATCAGTTTAATAAGATAAACCAGAGCGGCACGGCAAGCAGAGTATAC<br>GAGATTCGGTAGCCACCGCTGAGCAATAACGCGGCCGCAACTAGATAGCATAACCC<br>CTTGGGGCCTCTAAACGGGTCTTGAGGGGTTTTTTGCTGAAACCTCAGGCATTTGA<br>GAAGCACACGGTCACACTGCTTCCGGGGGAAATTAATCTCTTAATCCTTTTATCAT

	TCTACATTTAGGCGCTGCCATCTTGGCCGGCCATAACTTCGTATATTCGAACTTAT<br>ACGAACGGTAGCGGCCGCAACTAGAGGCATCAAATAAAACGAAAGGCTCAGTCGAA<br>AGACTGGGCCTTTCGTTTTATCTGTTGTTTGTCGGTGAACGCTCTCCTGAGTAGGA<br>CAAATCCGCCGCCCTAGACCTAGGGTACGGGTTTTGCTGCCCGCAAACGGGCTGTT<br>CTGGTGTTGCTAGTTTGTTATCAGAATCGCAGATCCGGCTTCAGCCGGTTTGCCGG<br>CTGAAAGCGCTATTTCTTCCAGAATTGCCATGATTTTTTCCCCACGGGAGGCGTCA<br>CTGGCTCCCGTGTTGTCGGCAGCTTTGATTCGATAAGCAGCATCGCCTGTTTCAGG<br>CTGTCTATGTGTGACTGTTGAGCTGTAACAAGTTGTCTCAGGTGTTCAATTTCATG<br>TTCTAGTTGCTTTGTTTTACTGGTTTCACCTGTTCTATTAGGTGTTACATGCTGTT<br>CATCTGTTACATTGTCGATCTGTTCATGGTGAACAGCTTTAAATGCACCAAAAACT<br>CGTAAAAGCTCTGATGTATCTATCTTTTTTACACCGTTTTCATCTGTGCATATGGA<br>CAGTTTTCCCTTTGATATCTAACGGTGAACAGTTGTTCTACTTTTGTTTGTTAGTC<br>TTGATGCTTCACTGATAGATACAAGAGCCATAAGAACCTCAGATCCTTCCGTATTT<br>AGCCAGTATGTTCTCTAGTGTGGTTCGTTGTTTTTGCGTGAGCCATGAGAACGAAC<br>CATTGAGATCATGCTTACTTTGCATGTCACTCAAAAATTTTGCCTCAAAACTGGTG<br>AGCTGAATTTTTGCAGTTAAAGCATCGTGTAGTGTTTTTCTTAGTCCGTTACGTAG<br>GTAGGAATCTGATGTAATGGTTGTTGGTATTTTGTCACCATTCATTTTTATCTGGT<br>TGTTCTCAAGTTCGGTTACGAGATCCATTTGTCTATCTAGTTCAACTTGGAAAATC<br>AACGTATCAGTCGGGCGGCCTCGCTTATCAACCACCAATTTCATATTGCTGTAAGT<br>GTTTAAATCTTTACTTATTGGTTTCAAAACCCATTGGTTAAGCCTTTTAAACTCAT<br>GGTAGTTATTTTCAAGCATTAACATGAACTTAAATTCATCAAGGCTAATCTCTATA<br>TTTGCCTTGTGAGTTTTCTTTTGTGTTAGTTCTTTTAATAACCACTCATAAATCCT<br>CATAGAGTATTTGTTTTCAAAAGACTTAACATGTTCCAGATTATATTTTATGAATT<br>TTTTTAACTGGAAAAGATAAGGCAATATCTCTTCACTAAAAACTAATTCTAATTTT<br>TCGCTTGAGAACTTGGCATAGTTTGTCCACTGGAAAATCTCAAAGCCTTTAACCAA<br>AGGATTCCTGATTTCCACAGTTCTCGTCATCAGCTCTCTGGTTGCTTTAGCTAATA<br>CACCATAAGCATTTTCCCTACTGATGTTCATCATCTGAGCGTATTGGTTATAAGTG<br>AACGATACCGTCCGTTCTTTCCTTGTAGGGTTTTCAATCGTGGGGTTGAGTAGTGC<br>CACACAGCATAAAATTAGCTTGGTTTCATGCTCCGTTAAGTCATAGCGACTAATCG<br>CTAGTTCATTTGCTTTGAAAACAACTAATTCAGACATACATCTCAATTGGTCTAGG<br>TGATTTTAATCACTATACCAATTGAGATGGGCTAGTCAATGATAATTACTAGTCCT<br>TTTCCTTTGAGTTGTGGGTATCTGTAAATTCTGCTAGACCTTTGCTGGAAAACTTG<br>TAAATTCTGCTAGACCCTCTGTAAATTCCGCTAGACCTTTGTGTGTTTTTTTTGTT<br>TATATTCAAGTGGTTATAATTTATAGAATAAAGAAAGAATAAAAAAAGATAAAAAG<br>AATAGATCCCAGCCCTGTGTATAACTCACTACTTTAGTCAGTTCCGCAGTATTACA<br>AAAGGATGTCGCAAACGCTGTTTGCTCCTCTACAAAACAGACCTTAAAACCCTAAA<br>GGCTTAAGTAGCACCCTCGCAAGCTCGGGCAAATCGCTGAATATTCCTTTTGTCTC<br>CGACCATCAGGCACCTGAGTCGCTGTCTTTTTCGTGACATTCAGTTCGCTGCGCTC<br>ACGGCTCTGGCAGTGAATGGGGGTAAATGGCACTACAGGCGCCTTTTATGGATTCA<br>TGCAAGGAAACTACCCATAATACAAGAAAAGCCCGTCACGGGCTTCTCAGGGCGTT<br>TTATGGCGGGTCTGCTATGTGGTGCTATCTGACTTTTTGCTGTTCAGCAGTTCCTG<br>CCCTCTGATTTTCCAGTCTGACCACTTCGGATTATCCCGTGACAGGTCATTCAGAC<br>TGGCTAATGCACCCAGTAAGGCAGCGGTATCATCAACAGGCTTACCCGTCTTACTG<br>TCCCTAGTGCTTGGATTCTCACCAATAAAAAACGCCCGGCGGCAACCGAGCGTTCT<br>GAACAAATCCAGATGGAGTTCTGAGGTCATTACTGGATCTATCAACAGGAGTCCAA<br>GCGAGCTCTCGAACCCCAGAGTCCCGCTCAGAAGAACTCGTCAAGAAGGCGATAGA<br>AGGCGATGCGCTGCGAATCGGGAGCGGCGATACCGTAAAGCACGAGGAAGCGGTCA<br>GCCCATTCGCCGCCAAGCTCTTCAGCAATATCACGGGTAGCCAACGCTATGTCCTG<br>ATAGCGGTCCGCCACACCCAGCCGGCCACAGTCGATGAATCCAGAAAAGCGGCCAT<br>TTTCCACCATGATATTCGGCAAGCAGGCATCGCCATGGGTCACGACGAGATCCTCG<br>CCGTCGGGCATGCGCGCCTTGAGCCTGGCGAACAGTTCGGCTGGCGCGAGCCCCTG<br>ATGCTCTTCGTCCAGATCATCCTGATCGACAAGACCGGCTTCCATCCGAGTACGTG<br>CTCGCTCGATGCGATGTTTCGCTTGGTGGTCGAATGGGCAGGTAGCCGGATCAAGC<br>GTATGCAGCCGCCGCATTGCATCAGCCATGATGGATACTTTCTCGGCAGGAGCAAG

	GTGAGATGACAGGAGATCCTGCCCCGGCACTTCGCCCAATAGCAGCCAGTCCCTTC<br>CCGCTTCAGTGACAACGTCGAGCACAGCTGCGCAAGGAACGCCCGTCGTGGCCAGC<br>CACGATAGCCGCGCTGCCTCGTCCTGCAGTTCATTCAGGGCACCGGACAGGTCGGT<br>CTTGACAAAAAGAACCGGGCGCCCCTGCGCTGACAGCCGGAACACGGCGGCATCAG<br>AGCAGCCGATTGTCTGTTGTGCCCAGTCATAGCCGAATAGCCTCTCCACCCAAGCG<br>GCCGGAGAACCTGCGTGCAATCCATCTTGTTCAATCATGCGAAACGATCCTCATCC<br>TGTCTCTTGATCAGATCTTGATCCCCTGCGCCATCAGATCCTTGGCGGCAAGAAAG<br>CCATCCAGTTTACTTTGCAGGGCTTCCCAACCTTACCAGAGGGCGCCCCAGCTGGC<br>AATTCCGACGTCTAAGAAACCATTATTATCATGACATTAACCTATAAAAATAGGCG<br>TATCACGAGGCCCTACCGTTCGTATATCTGTGGCTATACGAAGTTATCCTGCAGGA<br>GTGAGCTAACTTACATTAATTGCGTTGCGCTCACTGCCCGCTTTCCAGTCGGGAAA<br>CCTGTCGTGCCAGCTGCATTAATGAATCGGCCAACGCGCGGGGAGAGGCGGTTTGC<br>GTATTGGGCGCCAGGGTGGTTTTTCTTTTCACCAGTGAGACGGGCAACAGCTGATT<br>GCCCTTCACCGCCTGGCCCTGAGAGAGTTGCAGCAAGCGGTCCACGCTGGTTTGCC<br>CCAGCAGGCGAAAATCCTGTTTGATGGTGGTTAACGGCGGGATATAACATGAGCTG<br>TCTTCGGTATCGTCGTATCCCACTACCGAGATATCCGCACCAACGCGCAGCCCGGA<br>CTCGGTAATGGCGCGCATTGCGCCCAGCGCCATCTGATCGTTGGCAACCAGCATCG<br>CAGTGGGAACGATGCCCTCATTCAGCATTTGCATGGTTTGTTGAAAACCGGACATG<br>GCACTCCAGTCGCCTTCCCGTTCCGCTATCGGCTGAATTTGATTGCGAGTGAGATA<br>TTTATGCCAGCCAGCCAGACGCAGACGCGCCGAGACAGAACTTAATGGGCCCGCTA<br>ACAGCGCGATTTGCTGGTGACCCAATGCGACCAGATGCTCCACGCCCAGTCGCGTA<br>CCGTCTTCATGGGAGAAAATAATACTGTTGATGGGTGTCTGGTCAGAGACATCAAG<br>AAATAACGCCGGAACATTAGTGCAGGCAGCTTCCACAGCAATGGCATCCTGGTCAT<br>CCAGCGGATAGTTAATGATCAGCCCACTGACGCGTTGCGCGAGAAGATTGTGCACC<br>GCCGCTTTACAGGCTTCGACGCCGCTTCGTTCTACCATCGACACCACCACGCTGGC<br>ACCCAGTTGATCGGCGCGAGATTTAATCGCCGCGACAATTTGCGACGGCGCGTGCA<br>GGGCCAGACTGGAGGTGGCAACGCCAATCAGCAACGACTGTTTGCCCGCCAGTTGT<br>TGTGCCACGCGGTTGGGAATGTAATTCAGCTCCGCCATCGCCGCTTCCACTTTTTC<br>CCGCGTTTTCGCAGAAACGTGGCTGGCCTGGTTCACCACGCGGGAAACGGTCTGAT<br>AAGAGACACCGGCATACTCTGCGACATCGTATAACGTTACTGGTTTCACATTCACC<br>ACCCTGAATTGACTCTCTTCCGGGCGCTATCATGCCATACCGCGAAAGGTTTTGCG<br>CCATTCGATGGTGTCCGGGATCTCGACGCTCTCCCTTATGAGTGATAGCCGTTTGT<br>CTGGTGTCTACGCCGCGC

Table S3. Conditional gRNA sequences used in **Figure S3, S6, S19**. The common sequence element of a guide RNA scaffold was covered by a blue lane. Spacer sequences were shown as green region. Trigger binding region was covered by yellow lane.

Name	Sequence
V1	CUAUCUAAUCUGCUUGUUGAAUUAGGUGCCGCGGAAUACAUCUAAUUCAACAAGAAUUGUUUUAGA<br>GCUAGAAAUAGCAAGUUAAAAUAAGGCUAGUCCGUUAUCAACUUGAAAAAGUGGCACCGAGUCGGU<br>GCUUUUUU
V2	CUAUCUAAUCUGAAUUCUUGUUGAAUUACCUACACAUCUAAUUCAACAAGAAUUGUUUUAGAGCUA<br>GAAAUAGCAAGUUAAAAUAAGGCUAGUCCGUUAUCAACUUGAAAAAGUGGCACCGAGUCGGUGCUU<br>UUUU
0 nt bulge size	CUAUCUAAUCUGAAUUCUUGUUGAAUUAGAUGCCGCGGAAUACAUCUAAUUCAACAAGAAUUGUUU<br>UAGAGCUAGAAAUAGCAAGUUAAAAUAAGGCUAGUCCGUUAUCAACUUGAAAAAGUGGCACCGAGU<br>CGGUGCUUUUUU

1 nt bulge size	CUAUCUAAUCUGAAUUCUUGUUCAAUUAGAUGCCGCGGAAUACAUCUAAUUCAACAAGAAUUGUUUUAGAGCUAGAAAUAGCAAGUUAAAAUAAGGCUAGUCCGUUAUCAACUUGAAAAAGUGGCACCGAGUCGGUGCUUUUUU
2 nt bulge size	CUAUCUAAUCUGAAUUCUUGUCCAAUUAGAUGCCGCGGAAUACAUCUAAUUCAACAAGAAUUGUUUUAGAGCUAGAAAUAGCAAGUUAAAAUAAGGCUAGUCCGUUAUCAACUUGAAAAAGUGGCACCGAGUCGGUGCUUUUUU
3 nt bulge size	CUAUCUAAUCUGAAUUCUUGUCCUAUUAGAUGCCGCGGAAUACAUCUAAUUCAACAAGAAUUGUUUUAGAGCUAGAAAUAGCAAGUUAAAAUAAGGCUAGUCCGUUAUCAACUUGAAAAAGUGGCACCGAGUCGGUGCUUUUUU
4 nt bulge size (=V3)	CUAUCUAAUCUGAAUUCUUGGCCUAUUAGAUGCCGCGGAAUACAUCUAAUUCAACAAGAAUUGUUUUAGAGCUAGAAAUAGCAAGUUAAAAUAAGGCUAGUCCGUUAUCAACUUGAAAAAGUGGCACCGAGUCGGUGCUUUUUU
5 nt bulge size	CUAUCUAAUCUGAAUUCUUGGCCUCUUAGAUGCCGCGGAAUACAUCUAAUUCAACAAGAAUUGUUUUAGAGCUAGAAAUAGCAAGUUAAAAUAAGGCUAGUCCGUUAUCAACUUGAAAAAGUGGCACCGAGUCGGUGCUUUUUU
6 nt bulge size	CUAUCUAAUCUGAAUUCUUUGCCUCUUAGAUGCCGCGGAAUACAUCUAAUUCAACAAGAAUUGUUUUAGAGCUAGAAAUAGCAAGUUAAAAUAAGGCUAGUCCGUUAUCAACUUGAAAAAGUGGCACCGAGUCGGUGCUUUUUU
GFP conditional gRNA (20-nt extended loop)	CUAUCUAAUCUGAAUUCUUUGCCUCUUAGAUGCCGCGGAAUACAUCUAAUUCAACAAGAAUUGUUUUAGAGCUAGAAAUCGAGUCCACGUCAGUUAGCAAGUUAAAAUAAGGCUAGUCCGUUAUCAACUUGAAAAGUGGCACCGAGUCGGUGCUUUUUU

Table S4. Trigger sequences used in **Figure S8**. The stem binding sequences were covered by a blue lane. The toehold binding sequences were shown as green region. The T7 terminator sequence is shown in the purple lane.

Name	Sequence
Original trigger	CATCTAATAGGCCAAGAATTCAGATTAGATAGUAGAAACAGAUAGGCCCUCUUCGGAGGGCCUAUCUGUUUUUUUUU
Toehold -3	CATCTAATAGGCCAAGAATTCAGATTAGAUAGAAACAGAUAGGCCCUCUUCGGAGGGCCUAUCUGUUUUUUUUU
Toehold -6	CATCTAATAGGCCAAGAATTCAGATTUAGAAACAGAUAGGCCCUCUUCGGAGGGCCUAUCUGUUUUUUUUU
Toehold -9	CATCTAATAGGCCAAGAATTCAGUAGAAACAGAUAGGCCCUCUUCGGAGGGCCUAUCUGUUUUUUUUU
Toehold -12	CATCTAATAGGCCAAGAATTUAGAAACAGAUAGGCCCUCUUCGGAGGGCCUAUCUGUUUUUUUUU
Stem –4	TAATAGGCCAAGAATTCAGATTAGATAGUAGAAACAGAUAGGCCCUCUUCGGAGGGCCUAUCUGUUUUUUUUU
Stem -8	AGGCCAAGAATTCAGATTAGATAGUAGAAACAGAUAGGCCCUCUUCGGAGGGCCUAUCUGUUUUUUUUU

Stem -12	CAAGAATTCAGATTAGATAGUAGAAACAGAUAGGCCCUCUUCGGAGGGCCUAUCUGUUUUUUUUU
Stem -16	AATTCAGATTAGATAGUAGAAACAGAUAGGCCCUCUUCGGAGGGCCUAUCUGUUUUUUUUU
Stem -20	CAGATTAGATAGUAGAAACAGAUAGGCCCUCUUCGGAGGGCCUAUCUGUUUUUUUUU

Table S5. T7 promoter variants used in **Figure 3, S23**. The conserved sequence downstream of the transcription start site (TSS) of the T7 promoter, where transcription occurs, is highlighted in purple.

Name	Sequence	Transcription strength (RNA read / DNA read, according to the reference paper)
T7 promoter (OG)	TAATACGACTCACTATAGGG	188.15
T7 promoter (weak variant)	TGTTACGACTCACTATAGGG	37.41

Table S6. 2-input trigger sequences used in **Figure 2, 3, S13, S15, S17, S20, S23.** The T7 terminator sequence is shown in the purple lane.

Name	Logic	Sequence
X1.1	2-input AND	CAUCUAAUAGGCCAAGAAUUAAAGCAAGCCGGAGCAAGCUAUGGUAGCAUAACCCCUUGGGGCCUCUAAACGGGUCUUGAGGGGUUUUUU
X2.1	2-input AND	CAUCUAAUAGGCCAAGAAUAACGCAAGCCGGAGCAAGCUAUGGUAGCAUAACCCCUUGGGGCCUCUAAACGGGUCUUGAGGGGUUUUUU
X3.1	2-input AND	CAUCUAAUAGGCCAAGAAAACGCAAGCCGGAGCAAGCUAUGGUAGCAUAACCCCUUGGGGCCUCUAAACGGGUCUUGAGGGGUUUUUU
X4.1	2-input AND	CAUCUAAUAGGCCAAGACACGCAAGCCGGAGCAAGCUAUGGUAGCAUAACCCCUUGGGGCCUCUAAACGGGUCUUGAGGGGUUUUUU
Y1.1	2-input AND	CCAUAGCUUGCUCCGGCUUGCAAACAGAUUAGAUAGUAGCAUAACCCCUUGGGGCCUCUAAACGGGUCUUGAGGGGUUUUUU
TrA (toehold binding)	2-input OR	CAUCUAAUAGGCCAAGAAUUCAGAUUAGAUAGUAGCAUAACCCCUUGGGGCCUCUAAACGGGUCUUGAGGGGUUUUUU
TrB (loop binding)	2-input OR	UAUUCCGCGGCAUCUAAUAGGCCAAGAAUUUAGCAUAACCCCUUGGGGCCUCUAAACGGGUCUUGAGGGGUUUUUU
NOT Trigger A	NOT gate and 2-input A AND (NOT B) type 1	GUAGCUCUAAAACAAUUCUUGUUGAAUUAGAUGUAGCAAACCCCUUGGGGCCUCUAAACGGGUCUUGAGGGGUUUUUU
NOT Trigger Loop20	NOT gate and 2-input A	GACUGACGUGGACUCGAUUUCUAGCUCUAAAACAAUUCUUGUUGAAUUAGAUGUAGCAUAACCCCUUGGGGCCUCUAAACGGGUCUUGAGGGGUUUUUU

	AND (NOT B) type 1	
NOT Trigger Max20	NOT gate and 2-input A AND (NOT B) type 1	GCUUGCUAACUGACGUGGACUCGAUUUCUAGCUCUAAAACAAUUCUUGUUGAAUUAGAUGUAGCAUAACCCCUUGGGGCCUCUAAACGGGUCUUGAGGGGUUUUUU
NOT Trigger	2-input A AND (NOT B) type2	CAUCUAAUAGGCCAAGAAUUCAGAUUAGAUAGACAGUGUACAGGUAGCAUAACCCCUUGGGGCCUCUAAACGGGUCUUGAGGGGUUUUUU
NOT Anti-Trigger	2-input A AND (NOT B) type2	UAUGUAUGUGCUGUGACUAUCUAAUCUGAAUUCUUGGCCUAUUAGAUGUCCUUAUCUUAUCUGUUAGCAUAACCCCUUGGGGCCUCUAAACGGGUCUUGAGGGGUUUUUU
Decoy RNA 1		GACCGGUCUUUGCAGAGACCGGUGUAGAGACGACGAAUAGAAUUGAACAUAGAUAAGUAGCAUAACCCCUUGGGGCCUCUAAACGGGUCUUGAGGGGUUUUUU
Decoy RNA 2		GGCGUGAGAUAAGCACAUCUCACGAGACGAAGUAAAGCGUGUAAUCAAUAGUAGUAAGAAACAGAUAGGCCCUCUUCGGAGGGCCUAUCUGUUUUUUUUU

Table S7. 3-input trigger sequences used in **Figure 3**. The T7 terminator sequence was covered in the purple lane.

Name	Logic	Sequence
TrB (loop binding)	A OR (B AND C)	GUAUUCCGCGGAGGCCAGAUUAGAUAGUAGCAUAACCCCUUGGGGCCUCUAAACGGGUCUUGAGGGGUUUUUU
X4.1	A OR (B AND C)	GCUUCAGCUGCGUCGCUGAAGCGAUCAUCUAAUAGGCCAAGACACGCAAGCCGGAGCAAGCUAUGGUAGCAUAACCCCUUGGGGCCUCUAAACGGGUCUUGAGGGGUUUUUU
Y1.1	A OR (B AND C)	CGAGUGUUGUAUUACACUCGCUAGCCAUAGCUUGCUCCGGCUUGCAAACAGAUUAGAUAGAAACAGAUAGGCCCUCUUCGGAGGGCCUAUCUGUUUUUUUU

Table S8. Conditional gRNAs and sgRNAs used for metabolic regulation and morphology engineering by endogenous gene regulation. The common sequence element from a guide RNA scaffold was covered in the blue lane. Spacer sequences were indicated as green region. Trigger binding region was covered by yellow lane.

Name	Sequence
ftsZ conditional gRNA	CAAAUCCCAUAAAUGACGCGUUUUUUAAAGUCCCAUUCUCCUGACUUUAAUCACCGCGUCAUGUUUUAGAGCUAGAAAUAGCAAGUUAAAAUAAGGCUAGUCCGUUAUCAACUUGAAAAAGUGGCACCGAGUCGGUGCUUUUUU
ftsZ conditional gRNA (20-nt extended loop)	CAAAUCCCAUAAAUGACGCGUUUUUUAAAGUCCCAUUCUCCUGACUUUAAUCACCGCGUCAUGUUUUAGAGCUAGAAAUCGAAUCGUACCAGUAUAGCAAGUUAAAAUAAGGCUAGUCCGUUAUCAACUUGAAAAAGUGGCACCGAGUCGGUGCUUUUUUUUUU

mreB conditional gRNA	GGUCCAUCGUGUUCAUUUAUUCCUAAGGACAAUUACCGACAUUUGUCCUUUUACAUAAAUGAGUUUUAGAGCUAGAAAUAGCAAGUUAAAAUAAGGCUAGUCCGUUAUCAACUUGAAAAAGUGGCACCGAGUCGGUGCUUUUUU
lacZ conditional gRNA	CUACACGACACGGCACCGAUAAAACUUCCCAAUUCGUGCACCUUGGGAAGGGCGAUCGGUGCGUUUUAGAGCUAGAAAUAGCAAGUUAAAAUAAGGCUAGUCCGUUAUCAACUUGAAAAAGUGGCACCGAGUCGGUGCUUUUUU
malT conditional gRNA	CUUAUUCAUCAAAGCUUUUCCGUCAGCUGGCGAAUAUGCCUACGCCAGCUCAAUGAAAAGCUGUUUUAGAGCUAGAAAUAGCAAGUUAAAAUAAGGCUAGUCCGUUAUCAACUUGAAAAAGUGGCACCGAGUCGGUGCUUUUUU
poxB conditional gRNA	UAUGUGCCAUACUUAUCACACGGUGGAGAUGGAAUUACUCGCCCAUCUCCUGAAUGUGAUAAGUUUUAGAGCUAGAAAUAGCAAGUUAAAAUAAGGCUAGUCCGUUAUCAACUUGAAAAAGUGGCACCGAGUCGGUGCUUUUUU
aceE sgRNA (-1.7)	AGAGGUCGCCGCUGCCAGCCGUUUUAGAGCUAGAAAUAGCAAGUUAAAAUAAGGCUAGUCCGUUAUCAACUUGAAAAAGUGGCACCGAGUCGGUGCUUUUUU

Table S9. Trigger and decoy RNAs used for metabolic regulation and morphology engineering by endogenous gene regulation. The T7 terminator sequence is shown in the purple lane.

Name	Logic	Sequence
ftsZ Trigger	Single input	GACUUUAAAAAACGCGUCAUUUAUGGGAUUUGUAGCAUAACCCCUUGGGGCCUCUAAACGGGUCUUGAGGGGUUUUUU
ftsZ AND-A	Two-input AND	GGAGCGGCUGUCUCCGCUCAUAGACUUUAAAAAACGCGUCAUCACCAUUUCCAUUUUCCAUCCAUCUAGCAUAACCCCUUGGGGCCUCUAAACGGGUCUUGAGGGGUUUUUU
ftsZ AND-B	Two-input AND	GGACCUCACUGACGAGGUCAAGGAUGGAUGGAAAAUGGAAAUGAACUUAUGGGAUUUGUAGCAUAACCCCUUGGGGCCUCUAAACGGGUCUUGAGGGGUUUUUU
ftsZ OR-A	Two-input OR and Two-input A AND (NOT B)	GGACUUUAAAAAACGCGUCAUUUAUGGGAUUUGUAGCAUAACCCCUUGGGGCCUCUAAACGGGUCUUGAGGGGUUUUUU
ftsZ OR-B	Two-input OR	GAGGAGAAUGGGACUUUAAAAAACGCGUCAUUAGCAUAACCCCUUGGGGCCUCUAAACGGGUCUUGAGGGGUUUUUU
ftsZ NOT-B	Two-input A AND (NOT B)	GCUUGCUAUACUGGUACGAUUCGAUUUCUAGCUCUAAAACAUGACGCGGUGAUUAAAGUCUAGCAUAACCCCUUGGGGCCUCUAAACGGGUCUUGAGGGGUUUUUU
mreB Trigger	Single input	GUUGUCCUUAGGAAUAAAUGAACACGAUGGACCUAGCAUAACCCCUUGGGGCCUCUAAACGGGUCUUGAGGGGUUUUUU
lacZ Trigger	Single input	GUUGGGAAGUUUUAUCGGUGCCGUGUCGUGUAGUAGCAUAACCCCUUGGGGCCUCUAAACGGGUCUUGAGGGGUUUUUU
malT Trigger	Single input	GCGCCAGCUGACGGAAAAGCUUUGAUGAAUAAGUAGCAUAACCCCUUGGGGCCUCUAAACGGGUCUUGAGGGGUUUUUU
poxB Trigger	Single input	GCCAUCUCCACCGUGUGAUAAGUAUGGCACAUAUAGCAUAACCCCUUGGGGCCUCUAAACGGGUCUUGAGGGGUUUUUU
ftsZ Decoy1	Decoy	GCGUGAGAUAAGCACAUCUCACGAGACGAAGUAAAGCGUGUAAUCAAUAGUAGUAAGAAACAGAUAGGCCCUCUUCGGAGGGCCUAUCUGUUUUUUUUU

ftsZ Decoy2 and *mreB* decoy	Decoy	GACCGGUCUUUGCAGAGACCGGUGUAGAGACGACGAAUAGAAUUGAACAUAGAUAAGUAGCAUAACCCCUUGGGGCCUCUAAACGGGUCUUGAGGGGUUUUUU
lacZ Decoy (*ftsZ* ANDB)	Decoy	GGACCUCACUGACGAGGUCAAGGAUGGAUGGAAAAUGGAAAUGAACUUAUGGGAUUUGUAGCAUAACCCCUUGGGGCCUCUAAACGGGUCUUGAGGGGUUUUUU
malT Decoy	Decoy	GAUCCACGUUCUGCCACGUGGAUAACAGAUAUACGAGCGUAAGCGUGACCGGAGAUUUAGCAUAACCCCUUGGGGCCUCUAAACGGGUCUUGAGGGGUUUUUUG
poxB Decoy	Decoy	GCCUCGACGUUCGUGAUAACGUCGAGGCAAUCUAACCAUACCCAGCCUCAGUCUUAUUCGCAAUAGCAUAACCCCUUGGGGCCUCUAAACGGGUCUUGAGGGGUUUUU U

Table S10. Quantitative PCR primer sequences used in **Figure S26**.

Primer Name	Sequence (5′ to 3′)
PoxB_Fwd	GCCGGGAAAATTAAAGCGCCTA
PoxB_Rev	TCGGCGTTCATCATGGTATGGA

Table S11. Targeted next-generation sequencing primer sequences used in **Figure S27**.

Primer Name	Sequence (5′ to 3′)
dCas9_gRNA_PCR_fwd	CTAAGGCGTCGAGCAAAGCCCG
dCas9_gRNA_PCR_rev	TCCAGGAAATCTCCGCCCCGTT

Table S12. GS linker, AsiA activator, sgRNAs, cgRNA, Trigger RNA, and Decoy RNA used for compatibility test. The common sequence element from a guide RNA scaffold was covered in the blue lane. Spacer sequences were indicated as green region. The T7 terminator sequence was covered in the purple lane. Trigger binding region was covered by yellow lane.

Name	Type	Sequence
GS linker	Amino acids	GGGGSGGGGS
AsiA m2.1	Amino acids	MNKNIDTVREIITVASILIKFSREDIVENRANFIAFLNEIGVTHEGRKLNRNSFRKIISKLTQEDKKTLIDEFNEGFEGVYRYLEMYTNK
J23117 pro-moter	DNA	TTGACAGCTAGCTCAGTCCTAGGGATTGTGCTAGC
Gap se-quence	DNA	AGGAAAAGTAAGGAAAACGATTCCTTCTAACAGAAATGTCCTGAGCAATCACCTATGAACTGTCGACTCGAGCCTCTATGGATTATCACCTTGGCTGCAGGCCGGATCTTCCACAACACGCACGGTGTTACATTAGGCATACCGGTC

Guide RNA binding site	DNA	CTACGGAACTCTTGTGCGTA
H4 sgRNA	RNA	CUACGGAACUCUUGUGCGUAGUUUUAGAGCUAGAAAUAGCAAGUUAAAAUAAGGCUAGU CCGUUAUCAACUUGAAAAAGUGGCACCGAGUCGGUGCUUUUUU
Decoy sgRNA 1	RNA	CGAAACAAAGCGGUCUUACGGUUUUAGAGCUAGAAAUAGCAAGUUAAAAUAAGGCUAGU CCGUUAUCAACUUGAAAAAGUGGCACCGAGUCGGUGCUUUUUU
Decoy sgRNA 2	RNA	GUCAGUCGUAUUUCCUCUCGGUUUUAGAGCUAGAAAUAGCAAGUUAAAAUAAGGCUAGU CCGUUAUCAACUUGAAAAAGUGGCACCGAGUCGGUGCUUUUUU
Decoy sgRNA 3	RNA	GAAAGUCUUAGUACCGGACGGUUUUAGAGCUAGAAAUAGCAAGUUAAAAUAAGGCUAGU CCGUUAUCAACUUGAAAAAGUGGCACCGAGUCGGUGCUUUUUU
H4 cgRNA	RNA	CAUCUUGUCCCAUACGCACAUUCUUUCCGUAGAAACGAUCACCUACGGAACUCUUGUGC GUAGUUUUAGAGCUAGAAAUAGCAAGUUAAAAUAAGGCUAGUCCGUUAUCAACUUGAAA AAGUGGCACCGAGUCGGUGCUUUUUU
Decoy RNA	RNA	GACCGGUCUUUGCAGAGACCGGUGUAGAGACGACGAAUAGAAUUGAACAUAGAUAAGUAG CAUAACCCCUUGGGGCCUCUAAACGGGUCUUGAGGGGUUUUUU
Trigger RNA	RNA	CUACGGAAAGAAUGUGCGUAUGGGACAAGAUGUAGCAUAACCCCUUGGGGCCUCUAAACG GGUCUUGAGGGGUUUUUU

Table S13. Promoters, gRNA designs, trigger designs and induction conditions used for each experiment of main figures.

Logic and target gene	Figure	Promoter for cgRNA	cgRNA design	Trigger design	Induction condition
Single input, *gfp* target	1c-d	pLetO-1	4-nt bulge cgRNA	hairpinless	IPTG: 0.1 mM
					aTc: 0.5 ng/mL
					L-arabinose : 0.2% (w/w) (= 2 mg/mL)
					Induction time: 3h 30 min
Single input, *gfp* target	1e	pLtetO-1	4-nt bulge cgRNA	hairpinless	IPTG: 0.1 mM
					aTc: 0 ng/mL, 0.5 ng/mL
					L-arabinose: 0% ~ 0.2% (w/w)
					Induction time: 3h 30 min
2 input A OR B logic, A AND B logic, *gfp* target	2a-c	pLetO-1	4-nt bulge cgRNA	OR logic: hairpinless	IPTG: 0.1mM
					aTc: 0.5 ng/mL
	2d-f			AND logic: with hairpin, 3-nt gap	L-arabinose : 0.2% (w/w)
					Induction time: 3h 30 min
NOT A logic, *gfp* target	2g-i	pLetO-1	sgRNA with extended loop	hairpinless	IPTG: 0.1mM
					aTc: 0.1 ng/mL
					L-arabinose : 0.2% (w/w)
					Induction time: 3h 30 min
A AND NOT B-type 1, *gfp* target	3a-c	pLetO-1	4-nt bulge cgRNA with extended loop	hairpinless	IPTG: 0.1mM
					aTc: 0.5 ng/mL
					L-arabinose : 0.2%(w/w)
					Induction time: 3h 30 min
A AND NOT B-type 2, *gfp* target	3d-f	pLetO-1	4-nt bulge cgRNA	hairpinless	IPTG: 0.1mM
					aTc: 0.5 ng/mL
					L-arabinose : 0.2%(w/w)
					Induction time: 3h 30 min
3-input A OR (B AND C) logic, *gfp* target	3g-i	pLetO-1	4-nt bulge cgRNA	OR logic module: hairpinless	IPTG: 0.1mM
					aTc: 0.5 ng/mL
				AND logic module: with hairpin, 3-nt gap	L-arabinose : 0.2%(w/w)
					Induction time: 3h 30m
lacZ	4a-c	pLetO-1	4-nt bulge cgRNA	Hairpinless	aTc: 0.5 ng/mL
					L-arabinose : 0.2%(w/w)
					Induction time: 16h
malT	4d-f	pLetO-1	4-nt bulge cgRNA	Hairpinless	aTc: 0.5 ng/mL

					L-arabinose : 0.2%(w/w)
					Induction time: 16h
poxB	4g-i	pLetO-1	4-nt bulge cgRNA	Hairpinless	IPTG: 0.125mM
					aTc: 1 ng/mL
					L-arabinose : 0.2%(w/w)
					Induction time: 16h
ftsZ	5a-b	pLetO-1	4-nt bulge cgRNA	Hairpinless	IPTG: 0.1mM
					aTc: 0.5 ng/mL
					L-arabinose : 0.2%(w/w)
					Induction time: 3h 30 min
mreB	5c-d	pLetO-1	4-nt bulge cgRNA	Hairpinless	IPTG: 0.1mM
					aTc: 0.5 ng/mL
					L-arabinose : 0.2%(w/w)
					Induction time: 2h 30 min
lacZ	5e-f	pLetO-1	4-nt bulge cgRNA	Hairpinless	IPTG: 0.125mM
					aTc: 0.5 ng/mL
					L-arabinose : 0.2%(w/w)
					X-gal (200 µg/mL)
					Induction time: 4h 30 min
A OR B logic, *ftsZ*	6a-c	pLetO-1	4-nt bulge cgRNA	Hairpinless	IPTG: 0.1mM
					aTc: 0.5 ng/mL
					L-arabinose : 0.2%(w/w)
					Induction time: 3h 30 min
A AND B logic, *ftsZ*	6d-f	pLetO-1	4-nt bulge cgRNA	Hairpinless, no gap (0-nt)	IPTG: 0.1mM
					aTc: 0.5 ng/mL
					L-arabinose : 0.2% (w/w)
					Induction time: 7h
A and not B logic, *ftsZ*	6g-i	pLetO-1	4-nt bulge cgRNA with extended loop	Hairpinless	IPTG: 0.1mM
					aTc: 0.4 ng/mL
					L-arabinose : 0.2% (w/w)
					Induction time: 3h 30 min

Supplementary Figures

V1 design

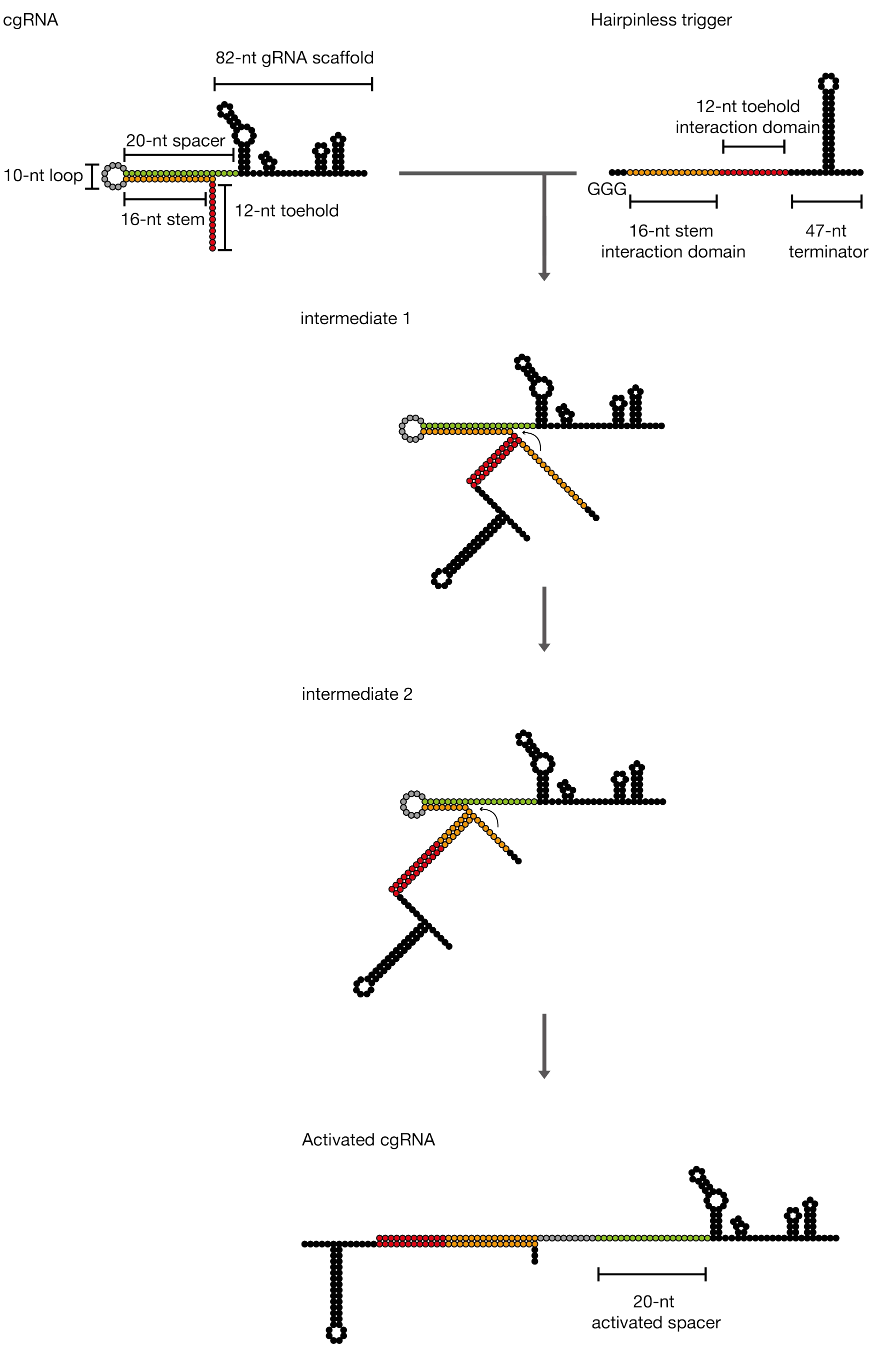

Figure S1. **Nucleotide-level schematics of V1 cgRNA.** V1 cgRNA employs a gRNA with a 12-nt toehold and a 16-nt stem to interact with a trigger RNA with a 28-nt single-stranded region. Binding of the trigger results in the formation of an active sgRNA. Black bases indicate biologically conserved sequences (e.g. terminators). Gray color indicates randomized sequences. Green bases indicate the spacer sequence. Orange and red bases indicate sequences determined by NUPACK based on the specified secondary structure.

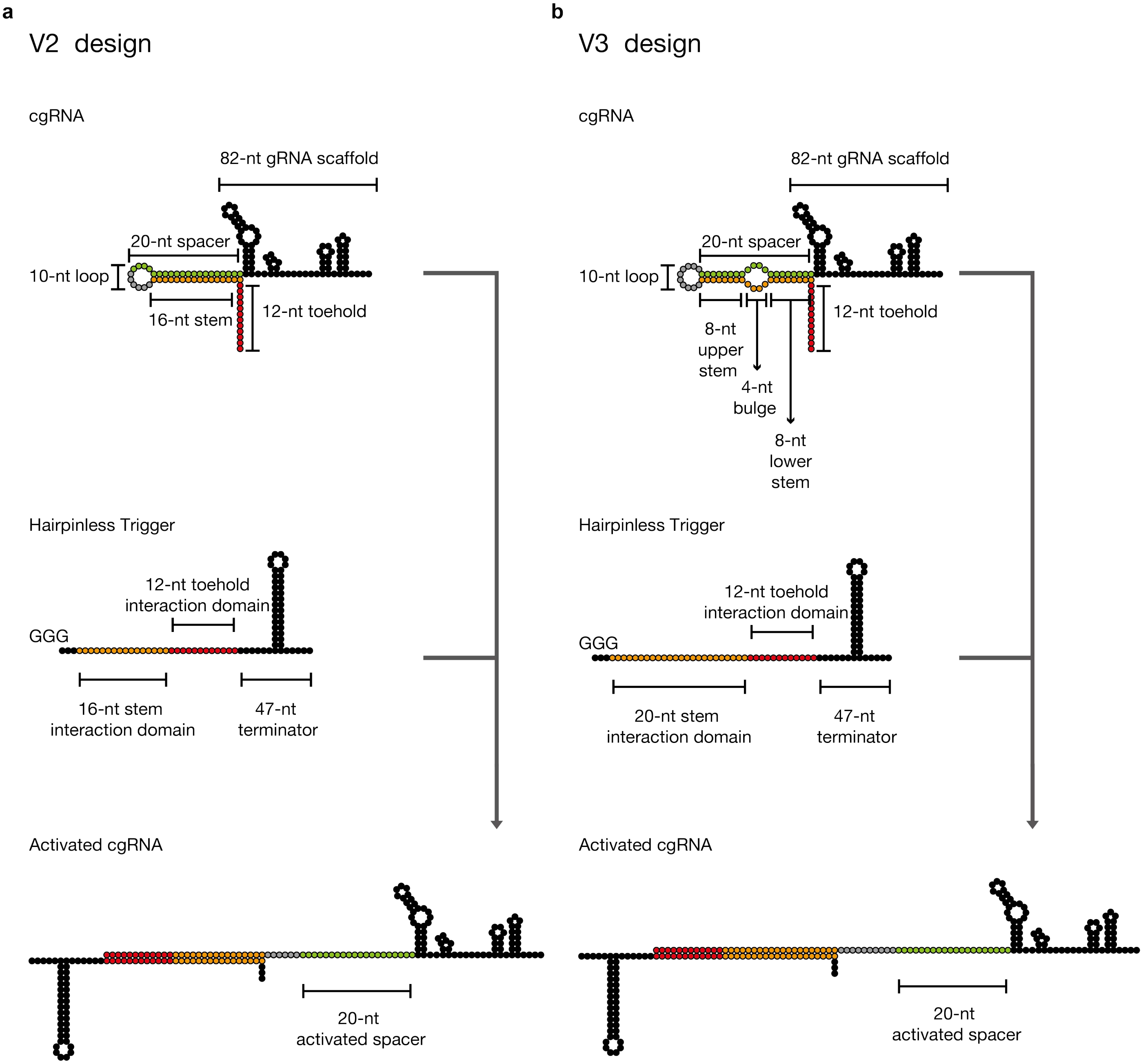

Figure S2. **Nucleotide-level schematics and mechanism-of-actions of V2 and V3 cgRNA**. V2 employ a gRNA with a 12-nt toehold and a 16-nt stem to interact with a trigger RNA with a 28-nt single-stranded region. V3 employ a gRNA with a 12-nt toehold and a 20-nt stem to interact with a trigger RNA with a 32-nt single-stranded region. Binding of the trigger results in the formation of an active sgRNA. Black bases indicate sequences that are biologically conserved (e.g. terminators). Gray color indicates randomized sequences. Green bases indicate the spacer sequence. Orange and red bases indicate sequences determined by NUPACK based on the specified secondary structure.

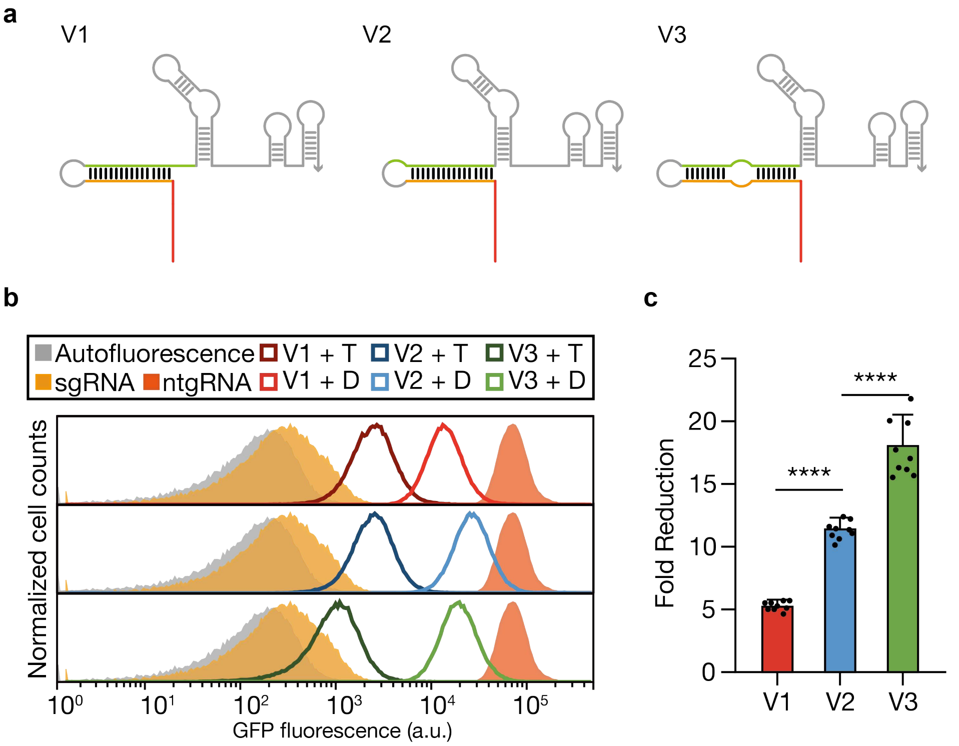

Figure S3. Flow cytometry data for V1, V2 and V3 with and without cognate trigger RNAs. Designs and flow cytometry data for V1, V2 and V3 with and without cognate trigger RNAs. (a) The design schematics of cgRNA V1, V2 and V3 are shown. They differ in the position of spacer-complementary binding domain of each cgRNA. (b) The cell population distributions of all samples were unimodal. In comparison to V1, both V2 and V3 exhibited high levels of GFP expression with inactive cgRNA, suggesting tight control over the inactivated cgRNAs. Compared to V2, V3 more effectively repressed the target gene in its active state. (c) Fold reduction of green fluorescent protein (GFP) fluorescence levels obtained 3h 30m after induction (****$P<0.0001$, Welch's unequal variances t-test). The number of biological replicates is three, and the figure shows a representative case.

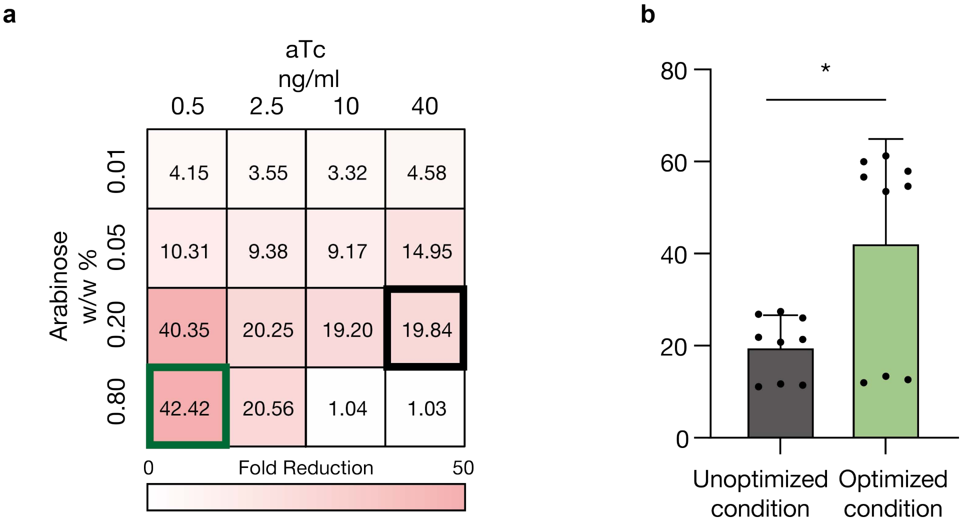

Figure S4. Fold reduction of GFP fluorescence regulated by V3 cgRNA. (a) CgRNA and input RNA expression were induced by arabinose, and dCas9 expression was induced by aTc. GFP expression was obtained when each cgRNA was expressed with cognate trigger RNA or with decoy RNA. (b) Fold reduction of two representative cases. Representative of unoptimized condition (at which we initially tested

cgRNA variants) is highlighted in black (a) and colored in gray (b). Representative of optimized condition is highlighted (a) and colored in green (b) (**P*<0.05, Welch's unequal variances t-test).

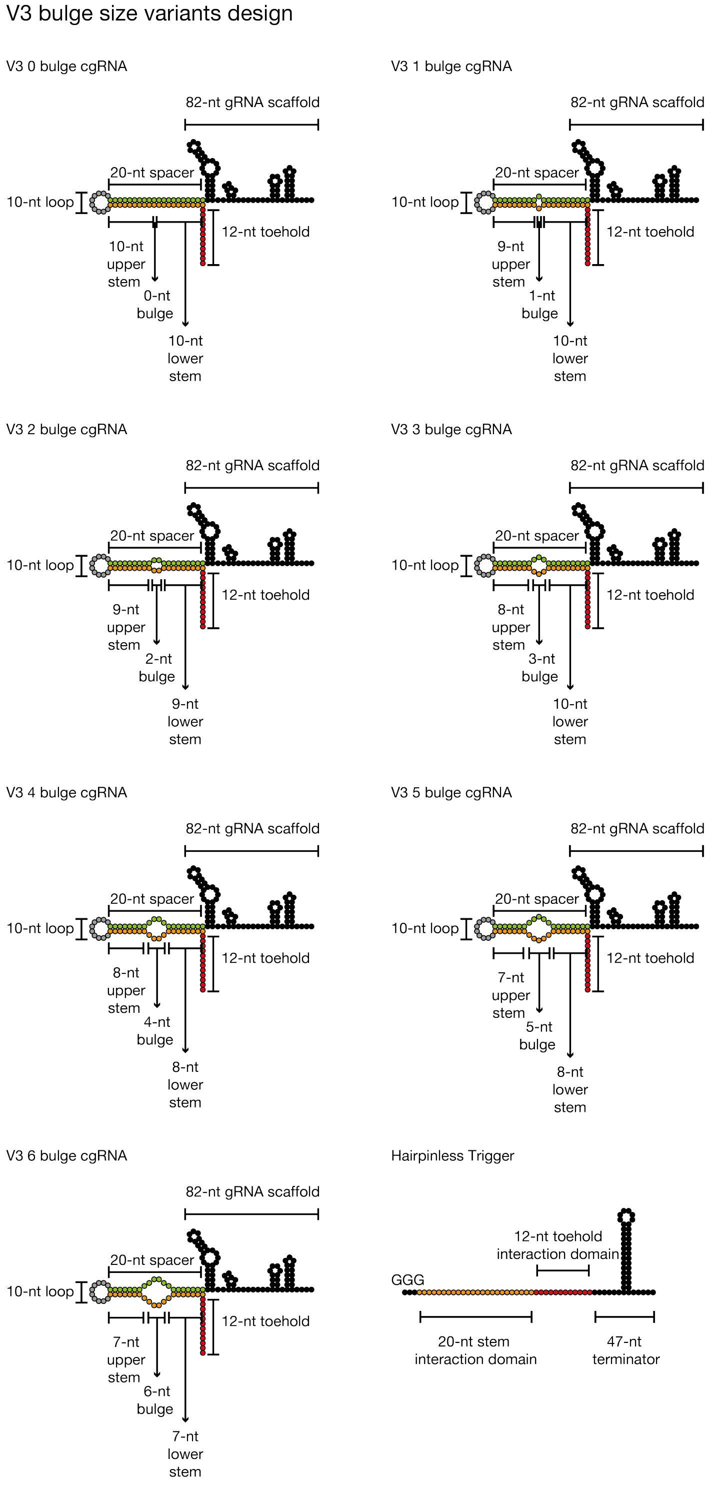

Figure S5. **Nucleotide-level schematics of V3 bulge size variants.** Each variant employs a gRNA with a 12-nt toehold and a 20-nt stem to interact with a trigger RNA. Each variant has unpaired nucleotides from 0 to 6 in the middle of the stem, which avoids unexpected effects of RNase III and weakens the stability of the RNA secondary structure. Since each cognate trigger RNA is complementary to each V3 variant, gRNA activation by a cognate trigger RNA is more favorable in 6 bulge cgRNA than in 2 bulge

cgRNA. Black bases indicate sequences that are biologically conserved (e.g. terminators). Gray bases indicate sequences that are randomizable. Green bases indicate the spacer sequence. Orange and red bases indicate sequences determined by NUPACK based on the specified secondary structure.

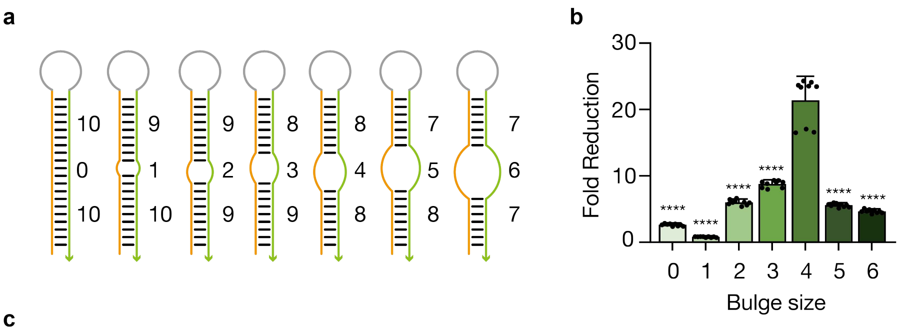

c

Welch's T test				Welch's T test			
Bulge size		p-value		Bulge size		p-value	
0	4	****	<0.0001	3	4	****	<0.0001
1	4	****	<0.0001	5	4	****	<0.0001
2	4	****	<0.0001	6	4	****	<0.0001

Figure S6. Bulge size variants of cgRNA V3 and its functionality shown as Fold reduction. (a) Bulge sizes varied from 0 to 6. Orange color indicates spacer-complementary sequence, and green color indicates spacer sequence. (b) Fold reduction of *gfp*-targeting cgRNA V3 bulge variants. (c) The statistical analysis tables show the combinatorial significance of 4-nt bulge cgRNA variant with other bulge variants (bulge size ranging from 0 to 6). Based on Welch's t-tests, ****$P<0.0001$ indicate conditions where the dynamic range of 4-nt bulge cgRNA variant is significantly improved, compared to other design variants.

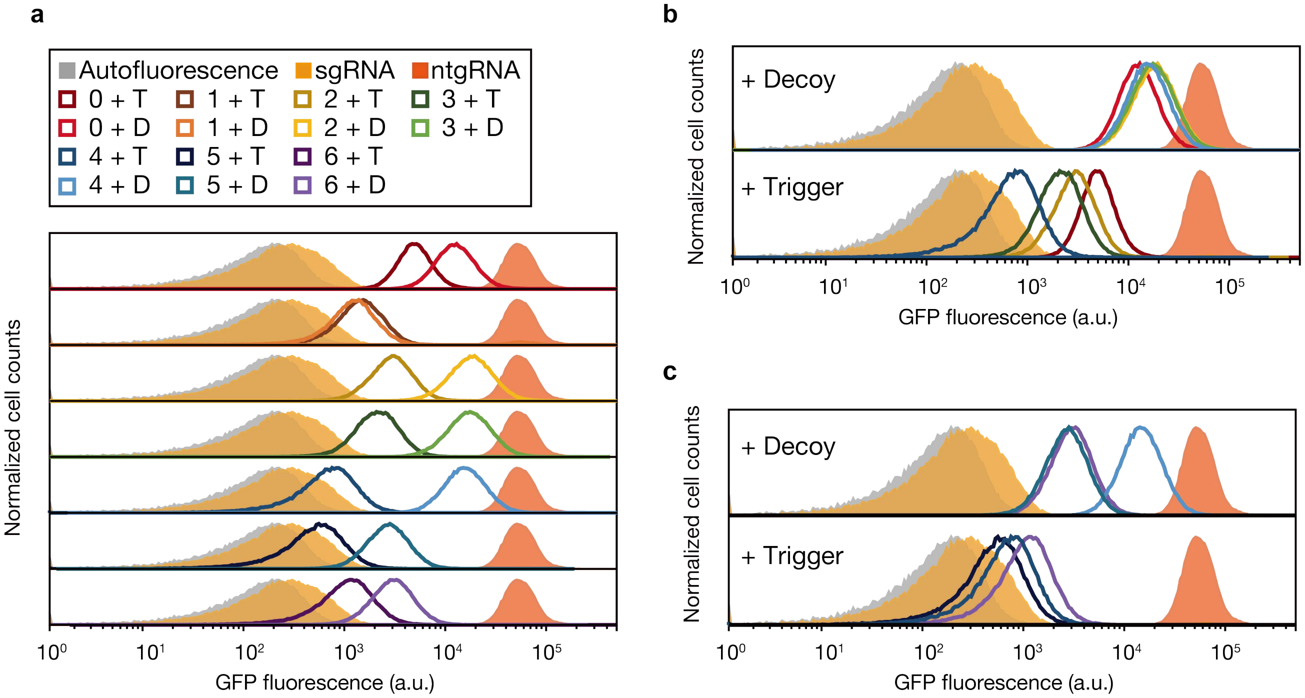

Figure S7. Flow cytometry GFP fluorescence histograms of V3 bulge variants. (a) Flow cytometry fluorescence histograms for V3 in its inactive state (with decoy RNA) are shown in brighter colors, and its active state (with trigger RNA) is shown in darker colors. (b) Flow cytometry GFP fluorescence histograms of V3 bulge variants from 0 to 4. (c) Flow cytometry GFP fluorescence histograms of V3 bulge variants from 4 to 6.

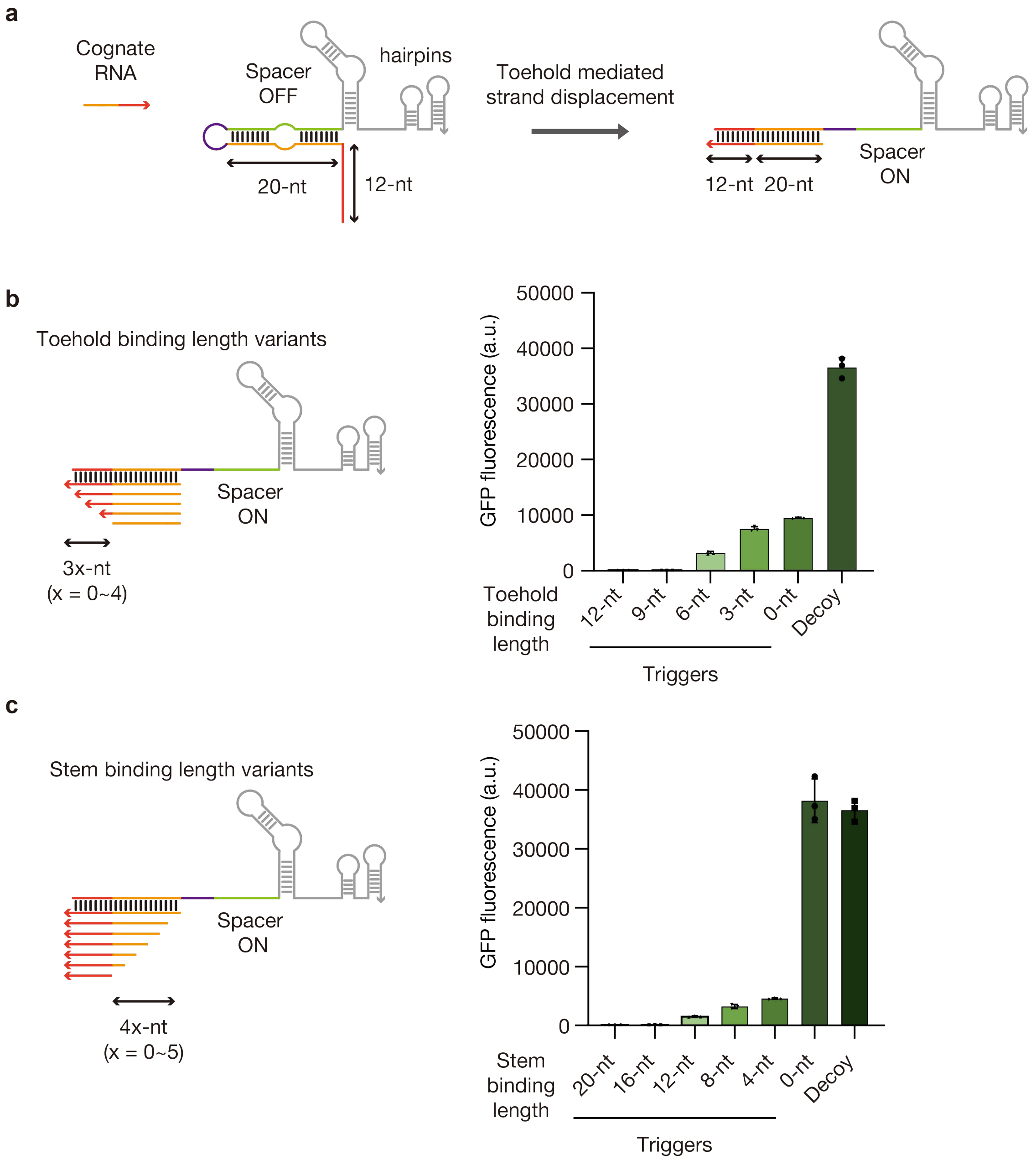

Figure S8. Determining a cgRNA design motif using trigger design variants of cgRNA V3 and its functionality shown as GFP fluorescence. (a) Schematics of the cognate RNA-mediated cgRNA activation and the lengths of two interacting domains (the toehold domain colored as red, and the stem-binding domain colored as orange). (b) The length of the toehold-binding domain of a trigger RNA varied from 12-nt to 0-nt. The domain length was truncated from the 3′ end of toehold-binding domain while the cgRNA was kept constant, so that the toehold-mediated strand displacement would be mitigated. (c) The length of the cgRNA stem-binding domain of a trigger RNA varied from 20-nt to 0-nt. The domain length was truncated from the 5′ end of stem-binding domain, so that the effect of strand-displacement would be mitigated, while the toehold-binding kinetic efficiency was kept constant.

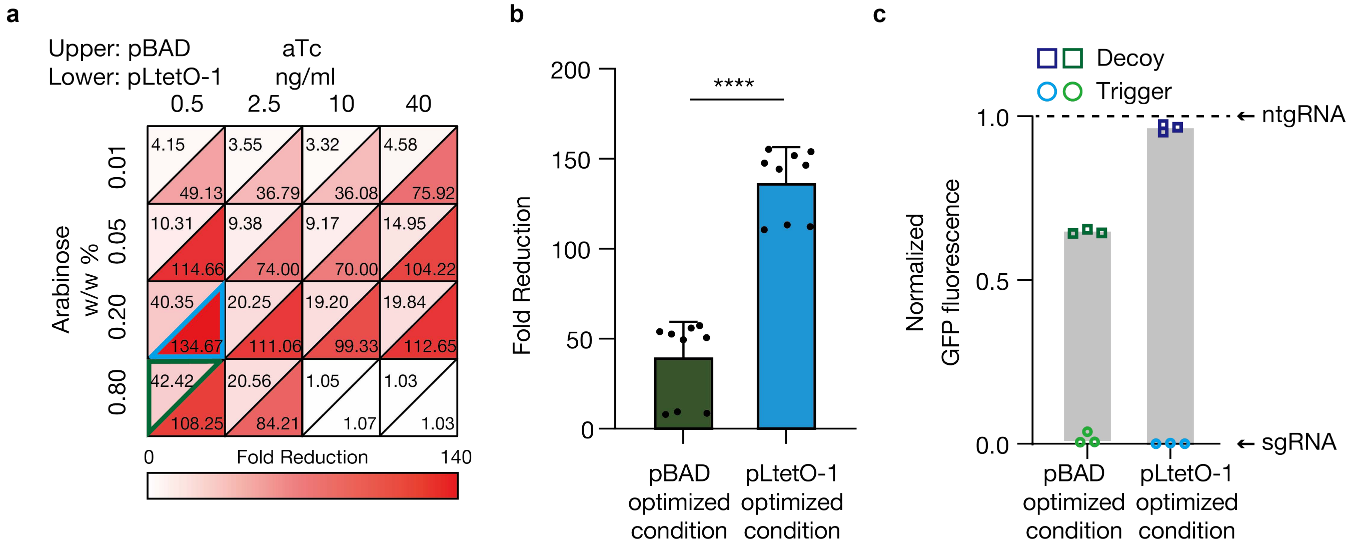

Figure S9. Fold reduction of GFP fluorescence. (a) GFP expression was regulated by cgRNA transcribed by either pBAD (upper) or pLtetO-1 (lower). GFP expression was obtained when each cgRNA was expressed with cognate trigger RNA or with decoy RNA. (b) Representative cases of optimized conditions. CgRNA is expressed with pBAD (left) or pLtetO-1 (right) promoter. (c) The normalized fluorescence was determined by subtracting the fluorescence of the activated cgRNA from that of the sgRNA control, and then dividing this difference by the discrepancy in fluorescence between a non-targeting gRNA and the sgRNA control. This computation can also be expressed using the equation: Normalized Fluorescence = $(\text{Fluorescence}_{\text{inactive/active cgRNA}} - \text{Fluorescence}_{\text{sgRNA}}) / (\text{Fluorescence}_{\text{ntgRNA}} - \text{Fluorescncence}_{\text{sgRNA}})$. The number of biological replicates is three. Based on Welch's t-test, ****$P<0.0001$, indicated condition that the fold reduction for the optimized condition using pLtetO-1 promoter is statistically significantly different from that of the optimized condition using pBAD promoter. The representative of the optimized condition is highlighted (a) and colored in green for pBAD and blue for pLtetO-1.

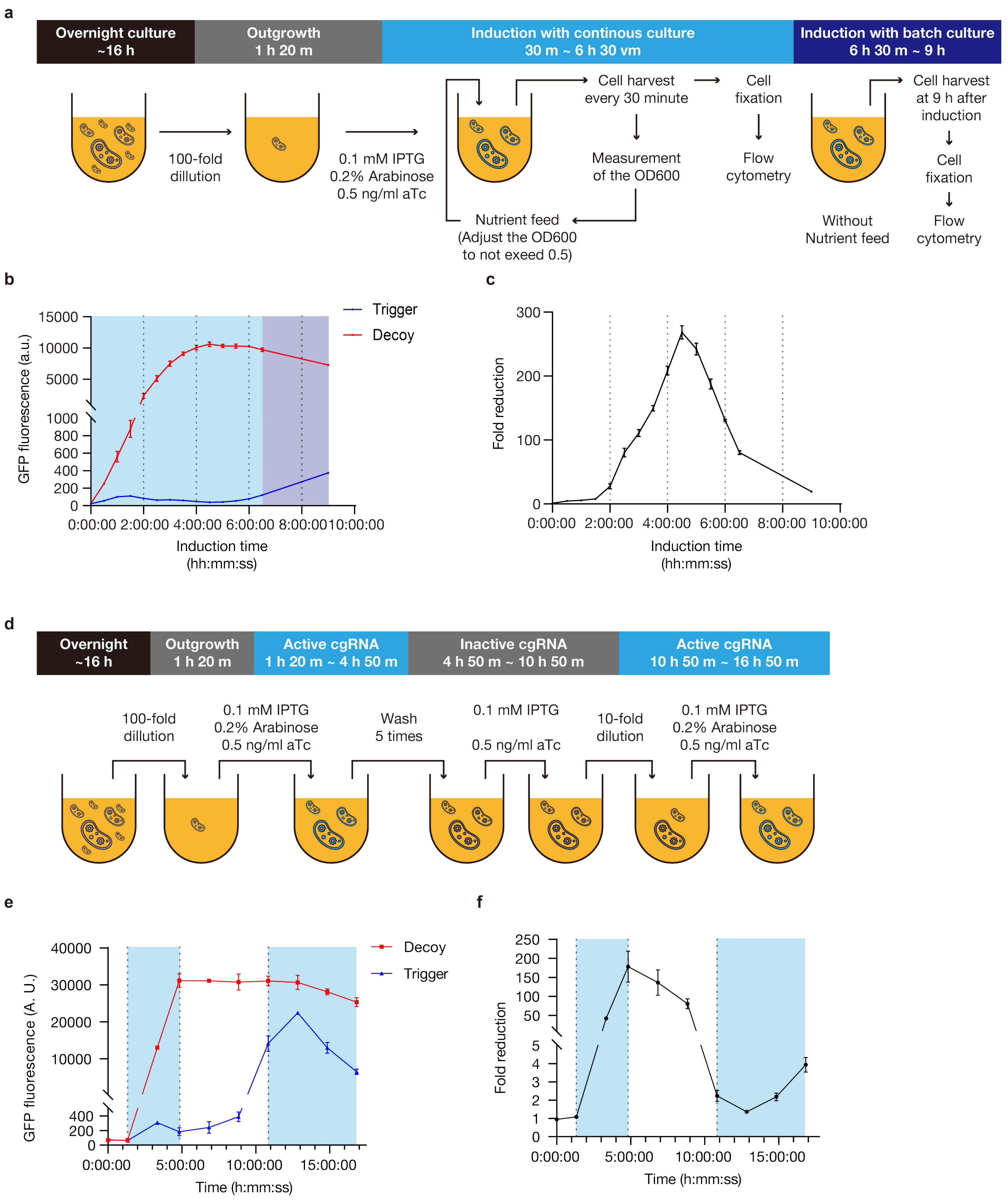

Figure S10. Time-course measurement of dynamic GFP expression and a cgRNA's functionality. (a) The graphical abstract of dynamically controlling a GFP-regulating cgRNA circuit by cgRNA activation or inactivation. (b) 200 µl of cells expressing activated GFP-targeting cgRNA (blue line) and inactive GFP-targeting cgRNA (red line) were sampled every 30 m from t=0 to t= 6h 30 m and last point at 9h. 200 µl of fresh LB media with inducers were replenished in each sampling point. GFP was measured by flow

cytometry, and the number of biological replicates is three. (c) The fold-reduction of cellular GFP level across each timepoint were shown. (d) The graphical abstract of reversible controlling of *gfp*-regulating cgRNA circuit by removing out the trigger inducer (L-arabinose) and re-inducing it. (e) Tracing reversibility of *gfp* regulation using cgRNA by inducing and removing triggers. 200 μl of cells were sampled at t=1h 20m, 3h 20m, 4h 50m. After washing out inducers for 5 times, 1 ml of fresh LB media with IPTG and aTc was replenished. Then, 200 μl of cells were sampled every 2 h for three times. Remaining 400 μl of cells were washed again and replenished with 1 ml of fresh LB media with IPTG, aTc and L-arabinose for re-induction of cgRNA-mediated CRISPRi circuit. 200 μl of cells were then sampled every 2 h for three times. (f) The fold-reduction of cellular GFP level across each timepoint were shown. The number of replicate is three, and error bars represent s.d. of n=3 biological replicates.

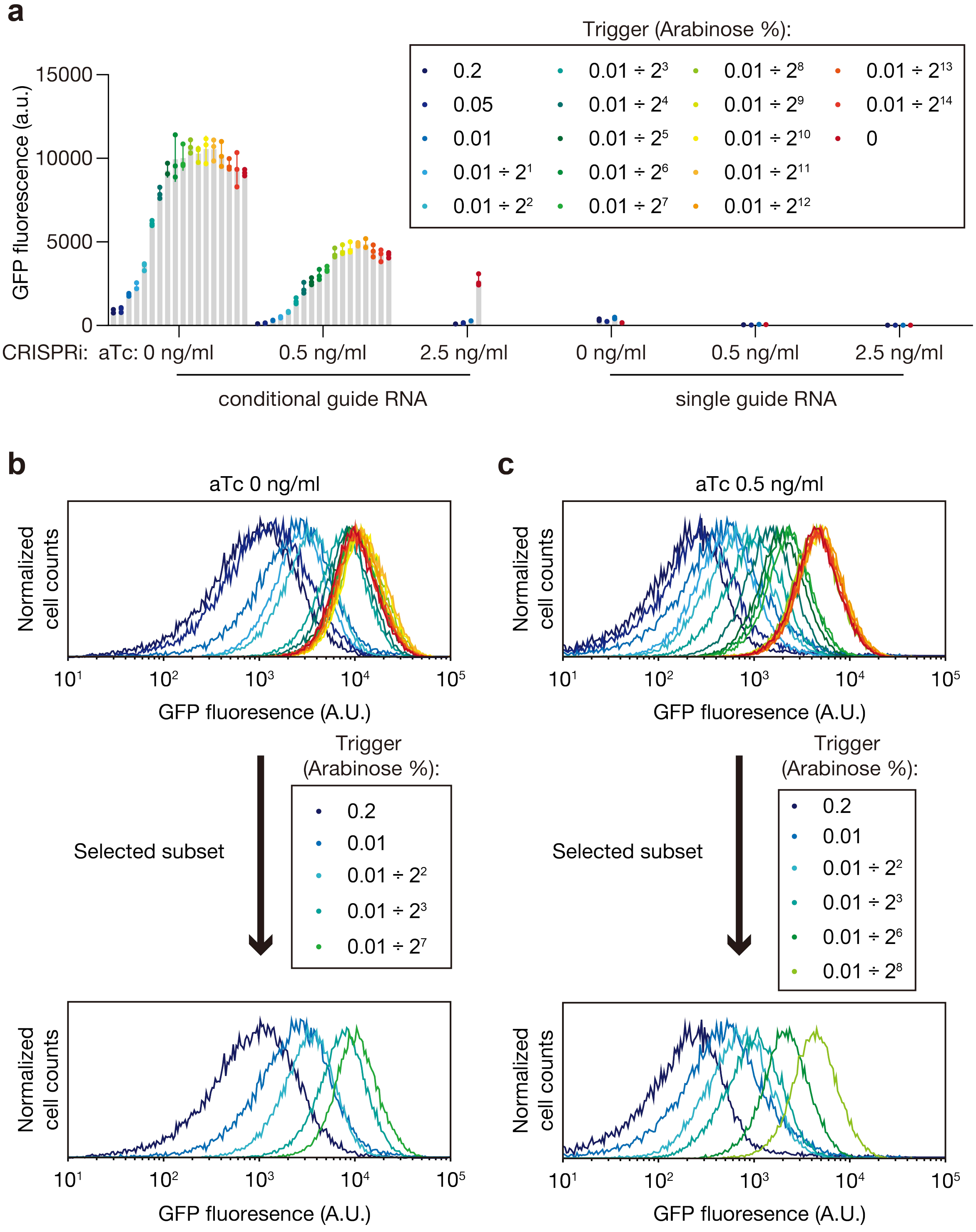

Figure S11. Regulation of GFP by gradient induction of L-arabinose and aTc. (a) By combining 18 different L-arabinose concentrations (ranging from 0.2% to 0%) and three aTc concentrations (0 ng/mL, 0.5 ng/mL and 2.5 ng/mL), we measured GFP fluorescence of a GFP-targeting cgRNA circuit-containing cells in various induction conditions. Cellular GFP levels were measured by flow cytometry, and the number of biological replicates is three. (b and c) Flow cytom-etry analysis was performed on 30,000 cells, and the GFP fluorescence and relative cell counts were visualized as histograms. The histograms represent the GFP fluorescence distribution for cells in-duced with 18 different L-arabinose concentrations, with a subset of conditions selectively dis-played for clarity. Five and six conditions were chosen to be displayed in the upper figures for better visualization.

V3 2-input OR gate design

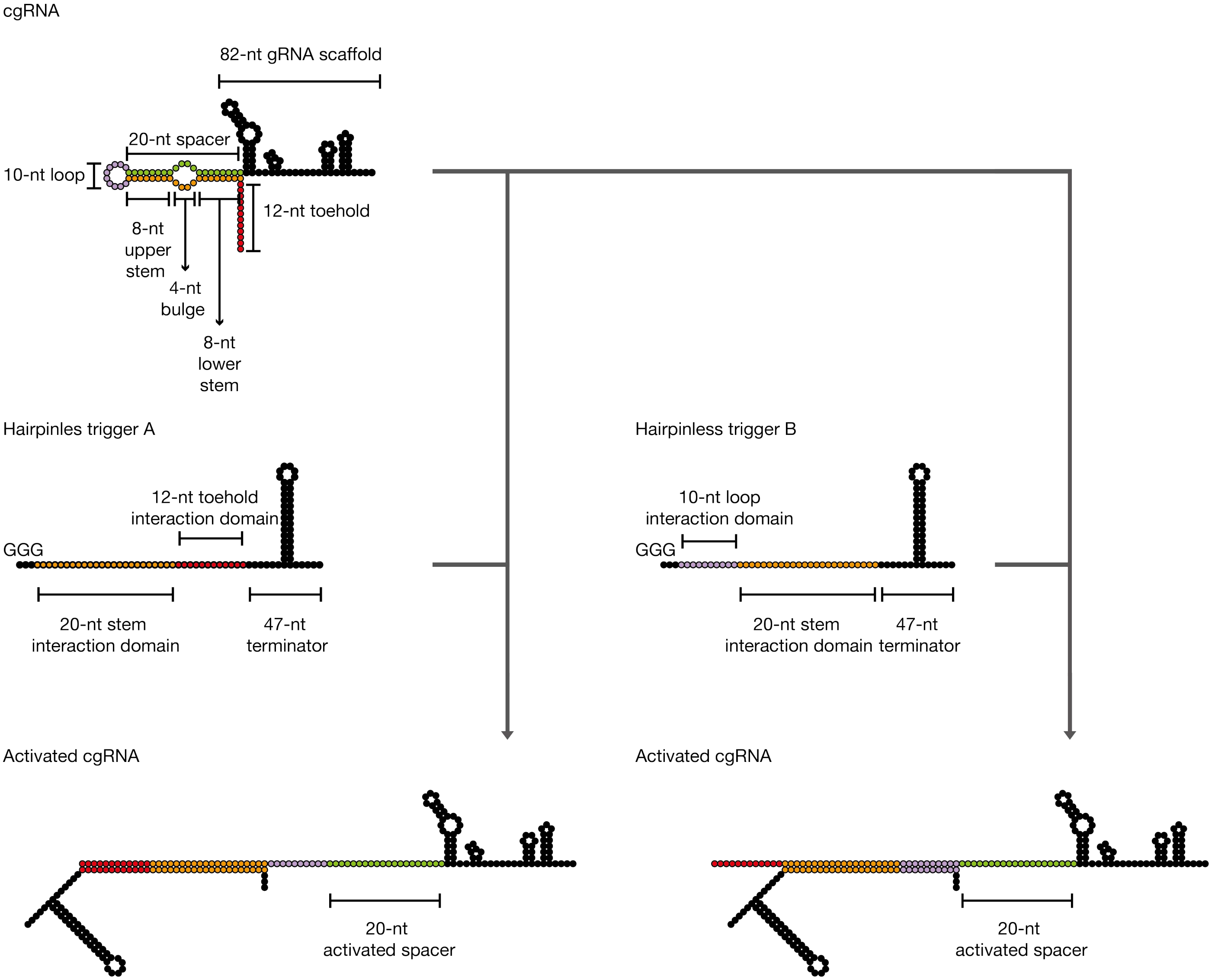

Figure S12. **Nucleotide-level schematics and mechanism-of-action of V3 2-input OR gate design**. V3 cgRNA has 10-nt loop, 20-nt stem and 12-nt toehold. Toehold and spacer-complementary region interacts with trigger A with a 12-nt toehold interaction domain and 20-nt stem interaction domain. Loop and spacer-complementary region interacts with trigger B with a 10-nt loop interaction domain and 20-nt stem interaction domain. Black bases indicate sequences that are biologically conserved (e.g. terminators). Green bases indicate the spacer sequence. Orange and red and pink bases indicate sequences determined by NUPACK based on the specified secondary structure.

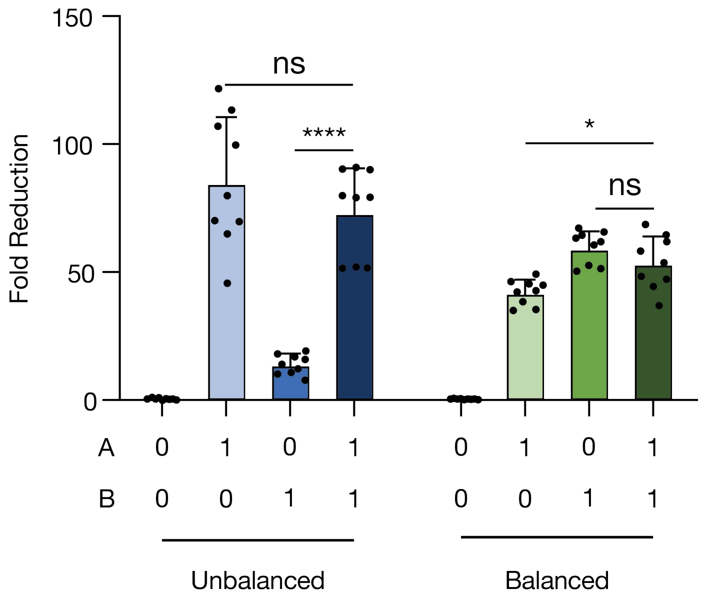

Figure S13. **Trigger plasmid copy number comparison used for 2-input OR logic circuits**. Fold reductions were calculated from GFP fluorescence obtained from a 2-input OR logic circuit. Histograms of left side (blue) were obtained by expression of toehold-binding trigger A from pBR322 origin plasmid and loop-binding trigger B from CloDF13 origin plasmid. Histograms on the right side (green) were obtained from expression of toehold-binding trigger A from CloDF13 origin plasmid and loop-binding trigger B from CloDF13 origin plasmid. The number of biological replicates is three. Based on Welch's t-tests, *$P<0.05$ and ****$P<0.0001$, indicate conditions where the fold reduction for the logical TRUE conditions are statistically significantly different from each other.

V3 2-input AND gate design - Hairpinless trigger

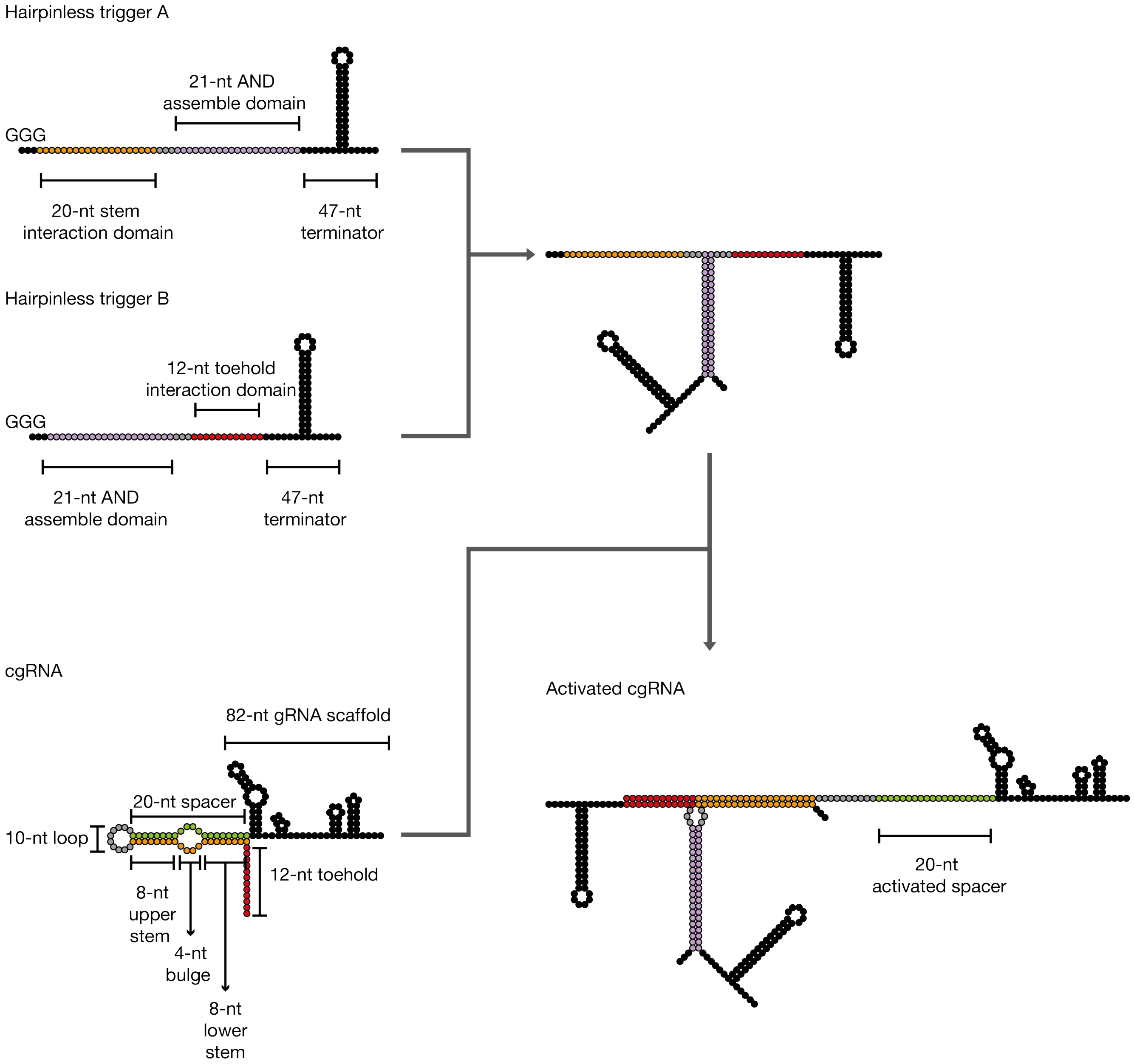

Figure S14. **Nucleotide-level schematics of V3 2-input AND gate "hairpinless" trigger design which triggers do not have 5′ hairpin**. Trigger A and trigger B assemble through a 21-nt AND assemble domain, resulting in a trigger which has 20-nt stem interaction domain and 12-nt toehold binding domain. Black bases indicate sequences that are biologically conserved (e.g. terminators). Green bases indicate the spacer sequence. Orange and red and pink bases indicate sequences determined by NUPACK based on the specified secondary structure.

a

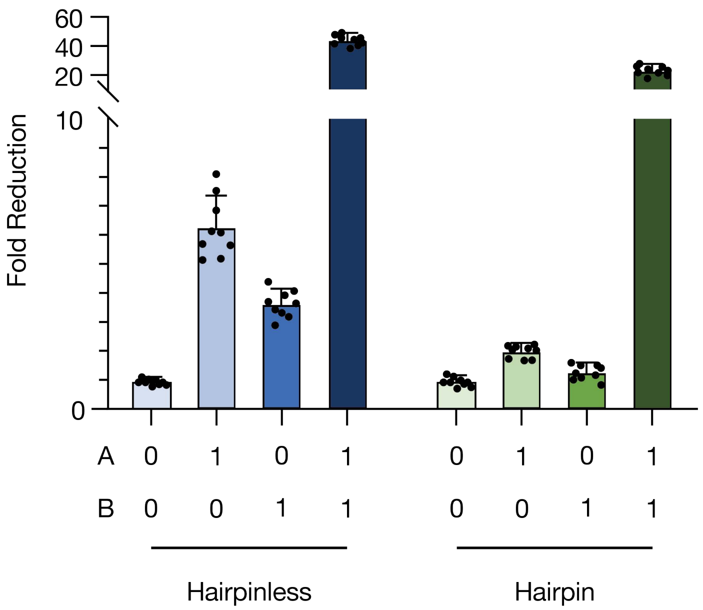

b

Welch's T test					
Hairpinless		Hairpin		p-value	
A	B	A	B		
0	0	0	0	n.s.	0.9999
1	0	1	0	****	<0.0001
0	1	0	1	****	<0.0001
1	1	1	1	****	<0.0001

Figure S15. **Comparison of two different trigger designs and their effects in 2-input AND logic circuit performances**. (a) GFP fluorescence histograms obtained from 2-input AND logic circuit. Left (blue) were obtained from expression of trigger A and B which do not have 5′ hairpin. Right (green) were obtained from expression of trigger A and trigger B which have 5′ hairpin. The number of biological replicates is three. (b) The statistical analysis result table shows the combinatorial significance of hairpinless versus hairpin for four different cases. Based on Welch's t-tests, *n.s.* means not significant, and ****$P<0.0001$ indicates conditions where the fold reductions for the triggers with hairpin cases are statistically significantly different from that of the highest of the triggers without hairpin cases.

V3 2-input AND gate design - Hairpin trigger

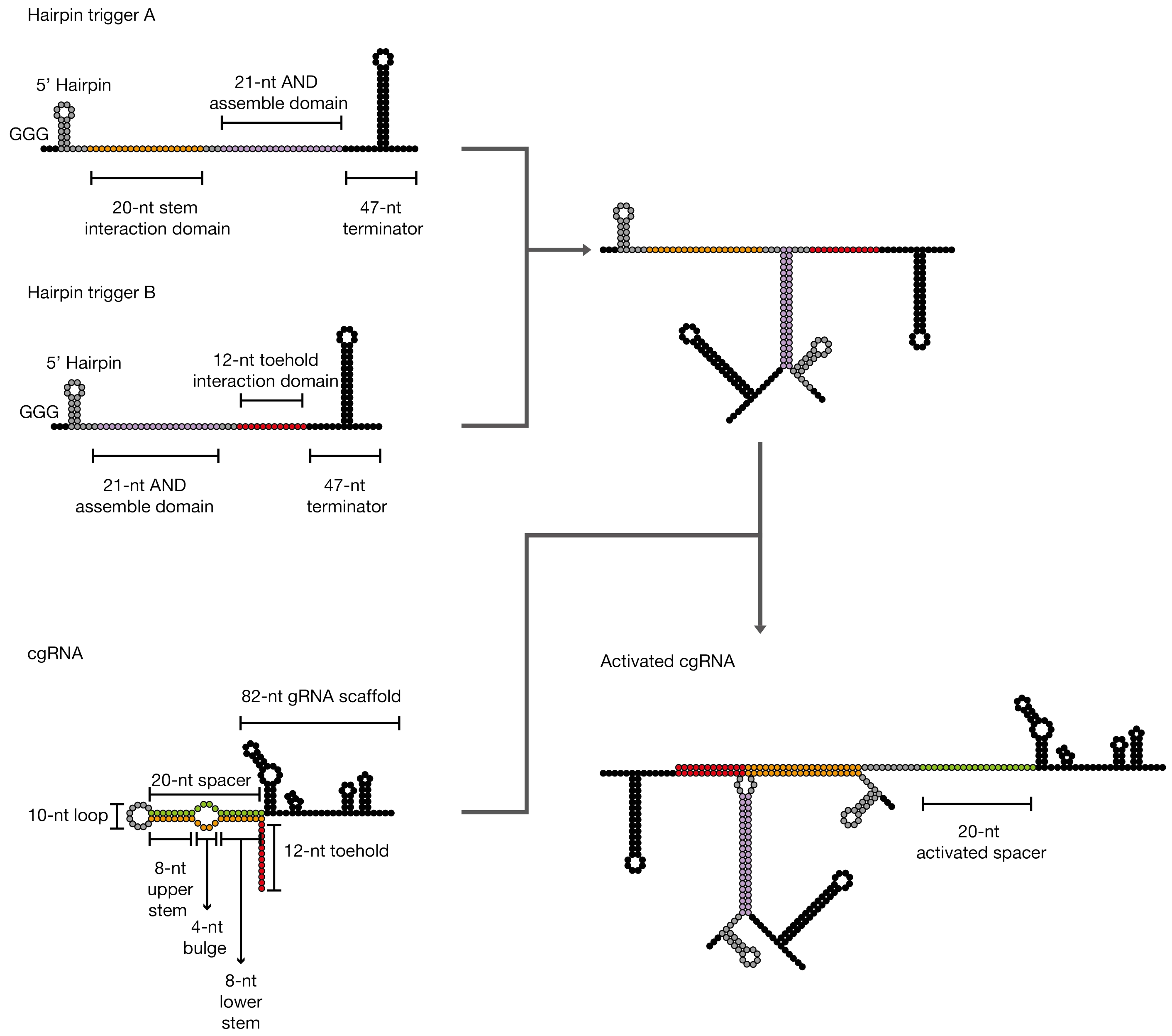

Figure S16. **Nucleotide-level schematics of V3 2-input AND gate "hairpin" trigger design which has 5′ hairpin**. Trigger A and trigger B assemble through 21-nt AND assemble domain, making a trigger which has 20-nt stem interaction domain and 12-nt toehold binding domain. Here both trigger A and trigger B have 5′ hairpin, which is expected to decrease the interaction efficiency between trigger and cgRNA. Black bases indicate sequences that are biologically conserved (e.g. terminators). Gray color indicates randomized sequences. Green bases indicate the spacer sequence. Orange and red and pink bases indicate sequences determined by NUPACK based on the specified secondary structure.

a

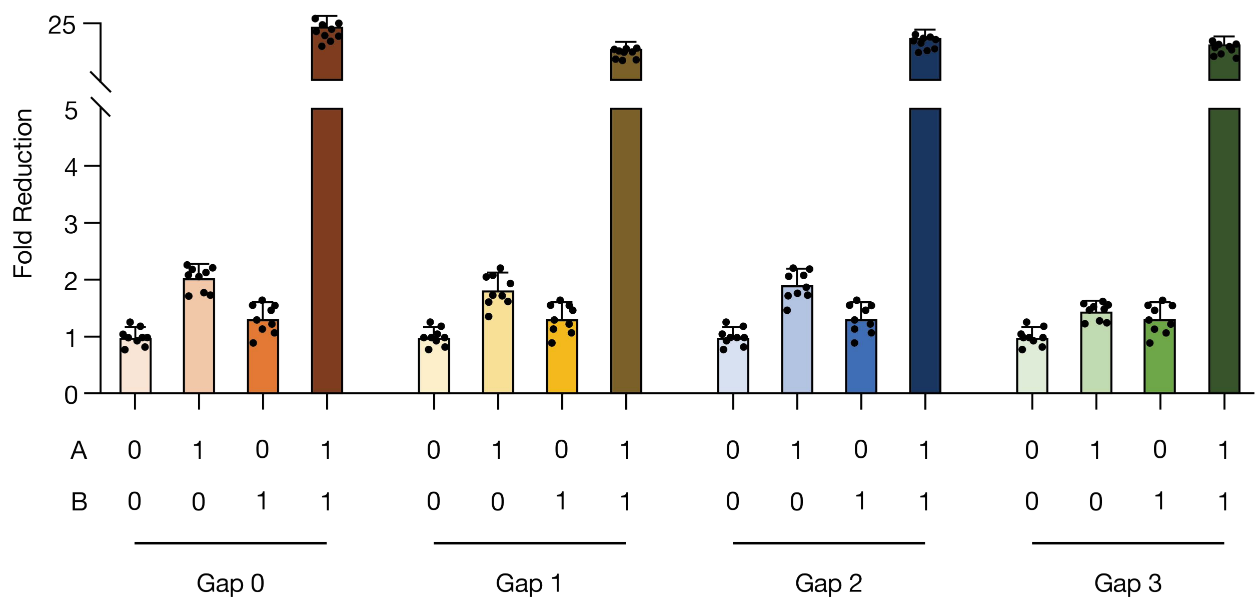

b

Welch's T test					
	A	B		p-value	
	1	0			
Gap 0			Gap 1	n.s.	0.0980
Gap 0			Gap 2	n.s.	0.3035
Gap 0			Gap 3	****	<0.0001

Welch's T test					
	A	B		p-value	
	1	1			
Gap 0			Gap 1	****	<0.0001
Gap 0			Gap 2	**	0.0089
Gap 0			Gap 3	***	0.0003

Welch's T test					
A	B	A	B	p-value	
1	0	0	1		
Gap 0		Gap 0		****	<0.0001
Gap 1		Gap 1		**	0.0011
Gap 2		Gap 2		***	0.0002
Gap 3		Gap 3		n.s.	0.2124

Figure S17. **Trigger RNA engineering for balanced FALSE value in two-input logic gate**. (a) In the first model (gap 0), we found that the output of A AND (NOT B) is significantly different from that of B AND (NOT A). This could interfere with the output of logic gates in a complex genetic network. To solve this problem, we created variants of Trigger A. These variants differ in the length of interaction domain of trigger RNAs, which reduces leaky activation of a cgRNA by a trigger A alone (Supplementary Figure S15). Gap 0 means trigger A, gap 1 means trigger A2, gap 2 means trigger A3 and gap 3 means trigger A4. (b) The statistical analysis tables show the combinatorial significance of leakage-exposed logical FALSE (1,0) case of Gap 0 with that of other Gap variants, logical TRUE (1,1) case of Gap 0 with that of other Gap variants, and leakage-exposed logical FALSE (1,0) cases of each Gap model with the other logical FALSE (0,1) cases of the models. We found that the output of A AND (NOT B) is not significantly

different from that of B AND (NOT A). The detailed description and nucleotide-level secondary RNA structure of the 2-input trigger variants are described in Fig. S18. Based on Welch's t-tests, **$P<0.01$, ***$P<0.001$ and ****$P<0.0001$ indicate conditions where the fold reductions of logical FALSE conditions are statistically significantly different from each other, which is not desired in a 2-input AND logic gate system.

V3 2-input AND gate design

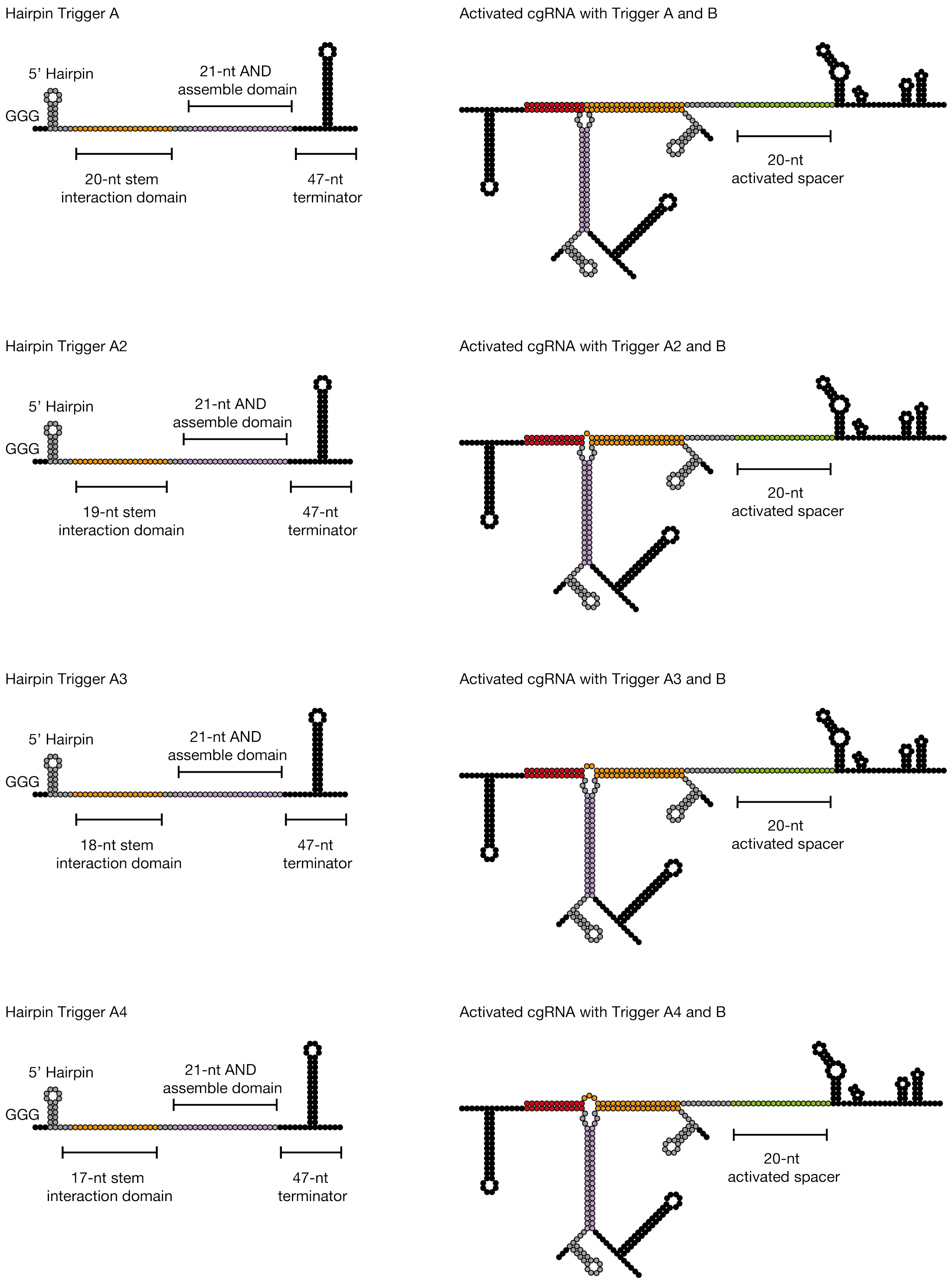

Figure S18. **Nucleotide-level schematics for cgRNA-based AND ribocomputing devices**. The two-input V3 cgRNA AND gate uses modified input RNAs that hybridize through complementary cgRNA domains (red and orange) and complementary trigger domains (purple). The trigger RNA sequence is divided into separate segments with a 21-nt AND assembly domain and variable stem interaction domains of 20-nt (Trigger A), 19-nt (Trigger A2), 18-nt (Trigger A3), and 17-nt (Trigger A4). The experimental results of these trigger designs are described in Figure S17.

Loop-20 sgRNA NOT gate design

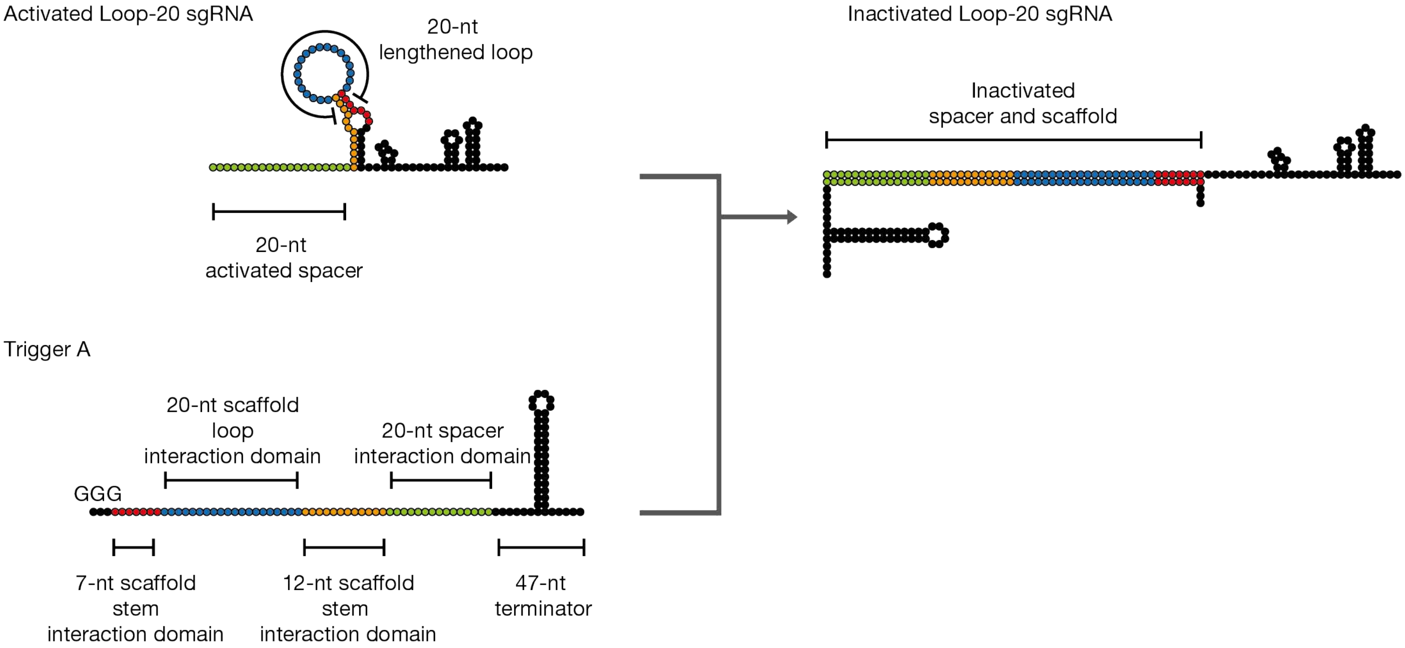

Figure S19. **Nucleotide-level schematics for NOT gate design**. The sgRNA NOT gate uses a modified sgRNA with an extended hairpin loop to enhance hybridization of NOT trigger A. Blue color indicates hairpin loop. Light green color indicates spacer. Orange and red bases indicate sequences that consists of gRNA scaffold and interact with trigger. The trigger sequence was designed to be complementary to gRNA scaffold.

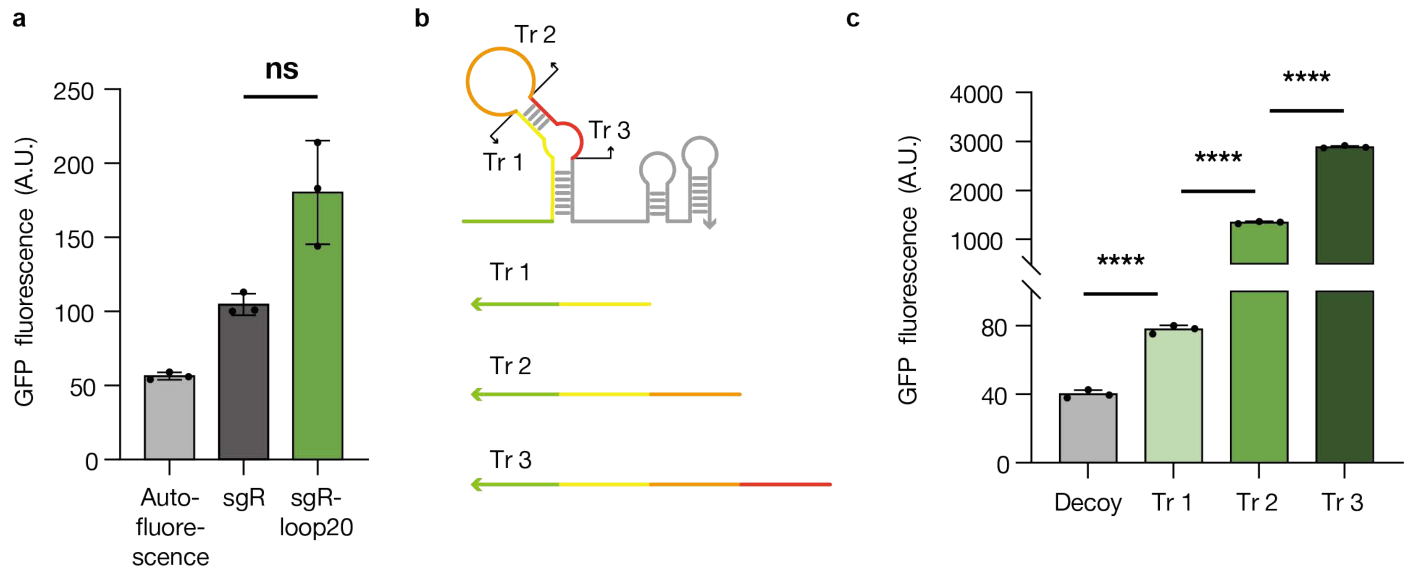

Figure S20. NOT gate trigger design optimization for proper gate building. Three types of gRNA scaffold-disrupting RNAs (NOT triggers) were tested with a GFP-repressing sgRNA that extended the tetraloop to 20 nt. (a) The knockdown efficiency of loop-extended sgRNA is compared to original sgRNA. (b) To modulate the scaffold disrupting effect of NOT triggers, we increased the scaffold complementary sequence to the NOT trigger. (c) GFP fluorescence of NOT logic-processing cells were analyzed. NOT logic gate was induced with proper inducer concentration {aTc 0.1 ng/mL, IPTG 0.1mM and L-arabinose 0.2%(w/w)}.

V3 2-input NOT gate 1 design

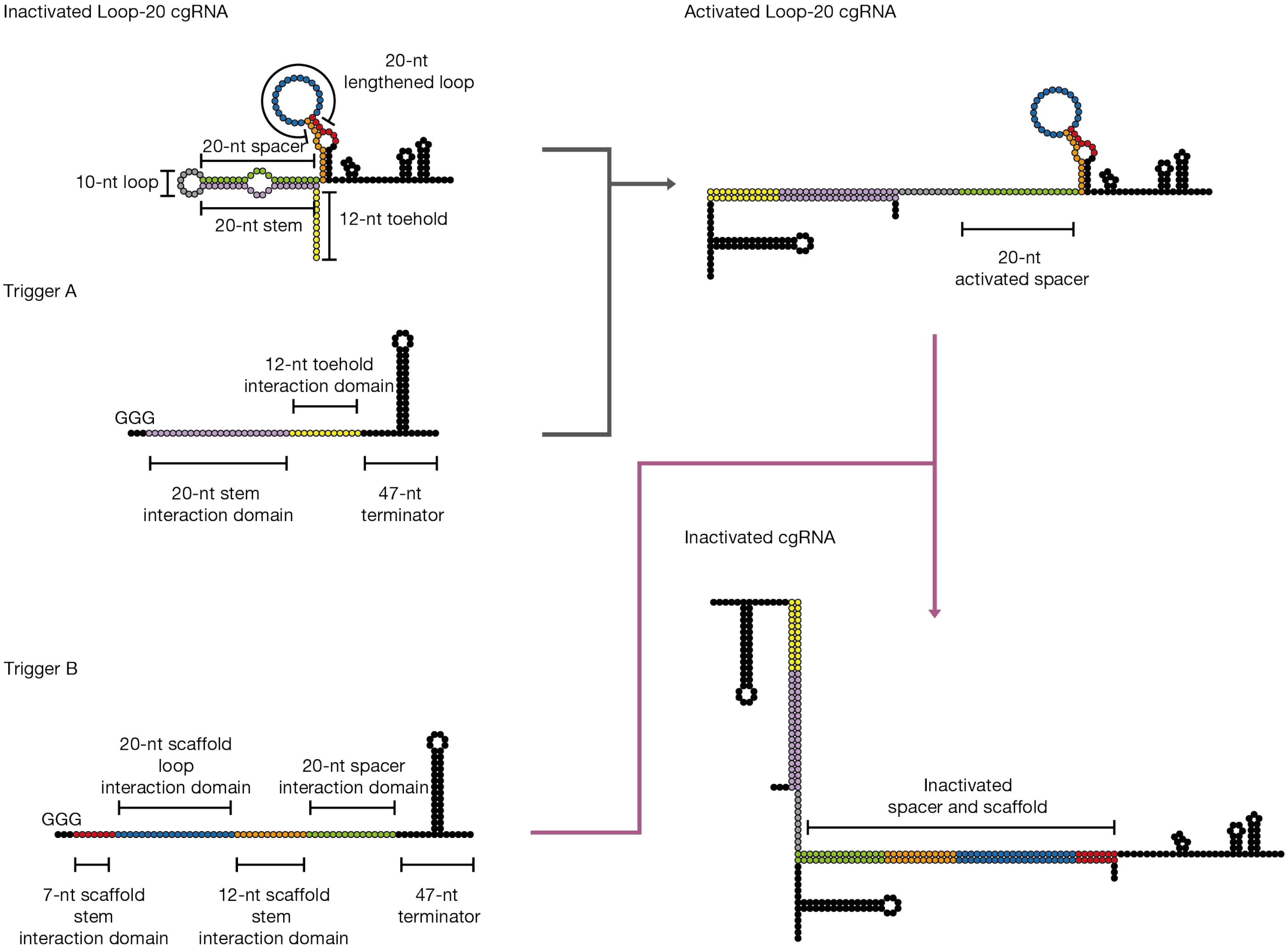

Figure S21. **Nucleotide-level schematics for 2-input A AND (NOT B) gate type 1 design**. The two-input A AND (NOT B) logic gate type 1 uses a modified gRNA scaffold with a 20-nt extended loop structure. Trigger A activates the cgRNA by exposing the spacer sequence, while trigger B inactivates the cgRNA by disrupting the scaffold so that it cannot interact with the Cas9 protein.

V3 2-input NOT gate design

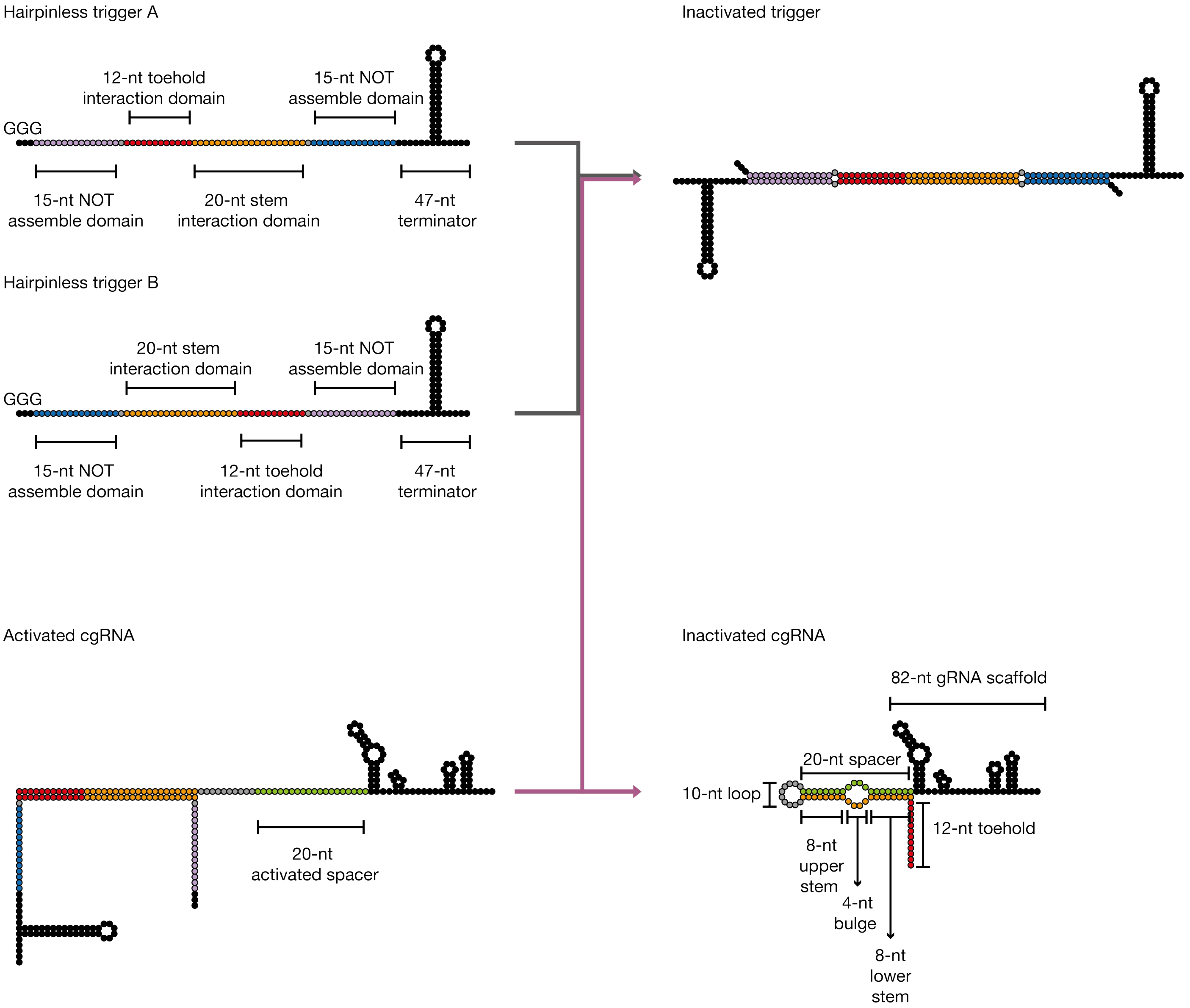

Figure S22. **Nucleotide-level schematics for 2-input A AND (NOT B) gate type 2 design**. The 2-input A AND (NOT B) gate type 2 uses modified input RNA A that hybridizes to cgRNA through cgRNA complementary domains (red and orange). Trigger B deactivates trigger A by hybridization of two 15-nt NOT-assemble domains (purple) and trigger A-complementary domains (red, orange). Since the interaction between trigger A and trigger B is more favorable than that between trigger A and cgRNA, trigger A is released from the cgRNA and remains inactivated.

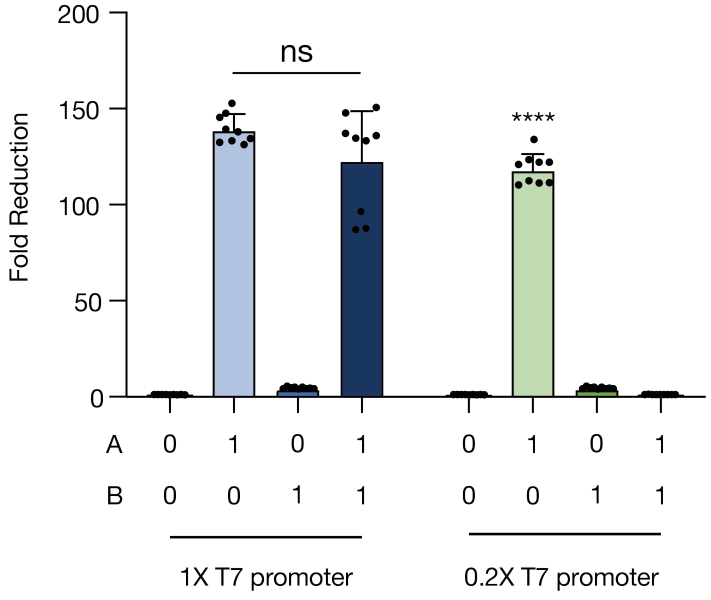

Figure S23. **2-input A AND (NOT B) gate trigger transcribing T7 promoter optimization for proper gate building.** For construction of 2-input NOT logic gate, two sets of T7 promoters were used for transcription of trigger RNAs and anti-trigger RNAs. 1X T7 promoter means that T7 promoter (OG) was used for transcription of both trigger and anti-trigger so that they are transcribed at the same rate per second. 0.2X T7 promoter means that T7 promoter (variant) was used for trigger transcription and T7 promoter (OG) was used for anti-trigger, so that anti-trigger is transcribed at the faster rate per second than the trigger (8).

V3 3-input A OR (B AND C) gate design

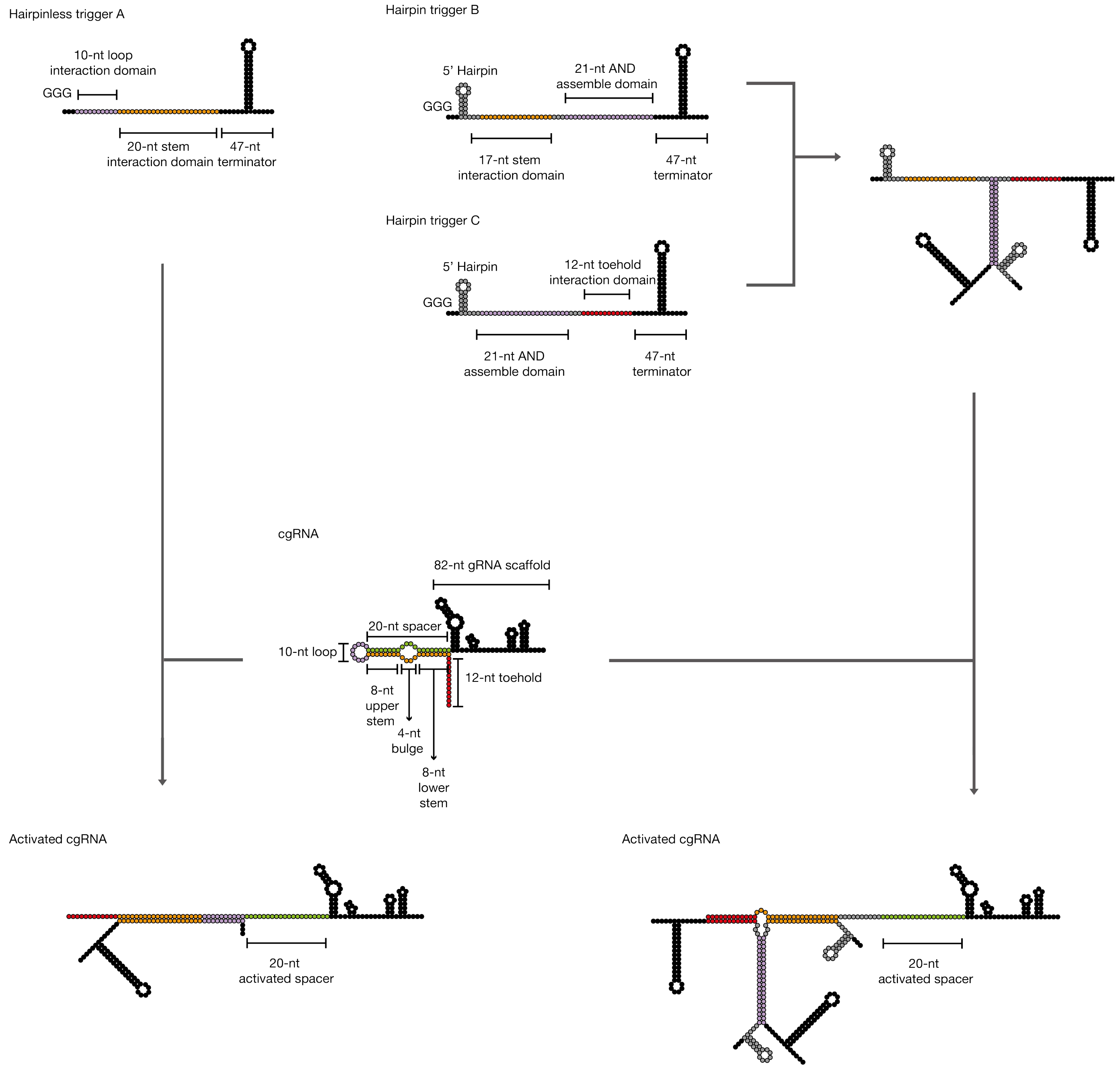

Figure S24. Nucleotide-level schematics for cgRNA-based A OR (B AND C) gate ribocomputing. The three-input cgRNA employs loop-binding input A that hybridizes and activates the cgRNA. The trigger B and C assemble through 21-nt AND assemble domain to form a complete trigger RNA that has 20-nt stem interaction domain and 12-nt toehold binding domain.

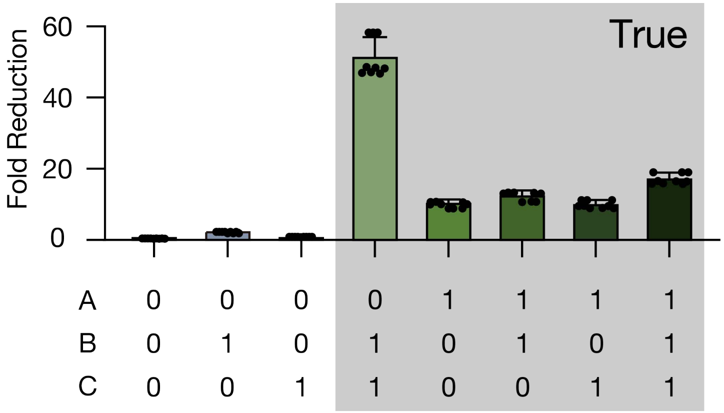

Welch's T test			
FALSE	TRUE		p-value
D D D	D B C	****	<0.0001
D D D	A D D	****	<0.0001
D D D	A B D	****	<0.0001
D D D	A D C	****	<0.0001
D D D	A B C	****	<0.0001

Welch's T test			
FALSE	TRUE		p-value
D B D	D B C	****	<0.0001
D B D	A D D	****	<0.0001
D B D	A B D	****	<0.0001
D B D	A D C	****	<0.0001
D B D	A B C	****	<0.0001

Welch's T test			
FALSE	TRUE		p-value
D D C	D B C	****	<0.0001
D D C	A D D	****	<0.0001
D D C	A B D	****	<0.0001
D D C	A D C	****	<0.0001
D D C	A B C	****	<0.0001

Figure S25. The statistical analysis of combinatorial significance of three logical FALSE cases with five logical TRUE cases for the 3-input A OR (B AND C) logic operation. The Three logical FALSE cases of three-input A OR (B AND C) logic operation (0,0,0), (0,1,0) and (0,0,1) were compared to the five logical TRUE cases (0,1,1), (1,0,0), (1,1,0), (1,0,1), and (1,1,1). The bar graph panel was added for convenience. Based on Welch's t-test, ****$P<0.0001$ indicate conditions where the fold reductions of logical FALSE cases are statistically significantly different from the logical TRUE cases.

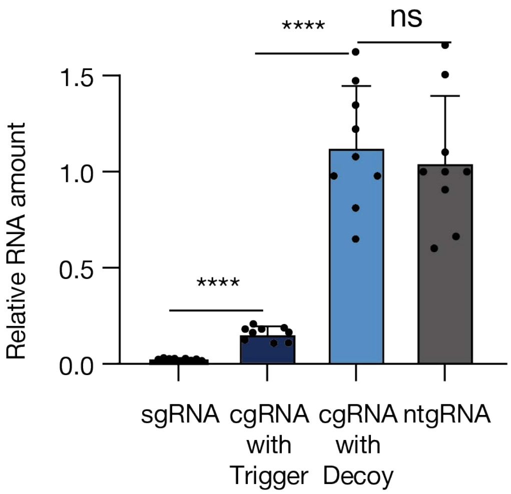

Figure S26. Comparison of relative abundance of *poxB* mRNA analyzed by reverse transcription quantitative PCR (RT-qPCR). Relative abundance of *poxB* mRNA for two controls (sgRNA for active gRNA control and ntgRNA for inactive gRNA control) and two states of a cgRNA (cgRNA with trigger for active state and cgRNA with decoy for inactive state). Data were normalized to 1 for the non-target gRNA state and error propagated. RT-qPCR data represent mean values and error bars represent s.d. of 9 possible reductions for three biological replications of inactive and active cgRNA states. Based on Welch's t-tests, ****$P<0.0001$, indicates conditions where the relative RNA amount for the logical TRUE case (cgRNA with trigger) is significantly different from that for the logical FALSE case (cgRNA with decoy).

a

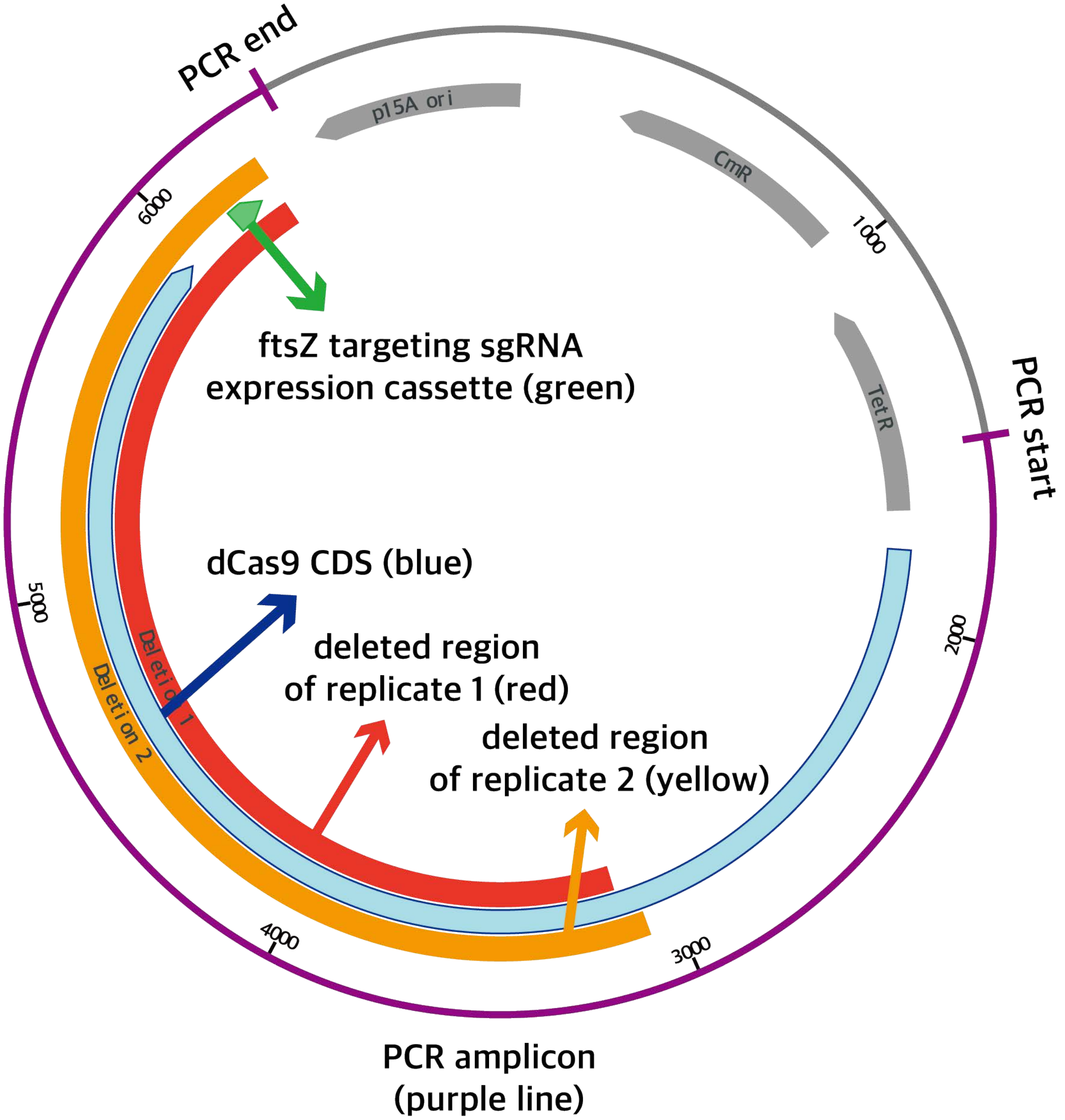
PCR end
p15A ori
CmR
1000
PCR start
TetR
6000
ftsZ targeting sgRNA
expression cassette (green)
dCas9 CDS (blue)
5000
2000
Deletion 1
Deletion 2
deleted region
of replicate 1 (red)
deleted region
of replicate 2 (yellow)
4000
3000
PCR amplicon
(purple line)

b

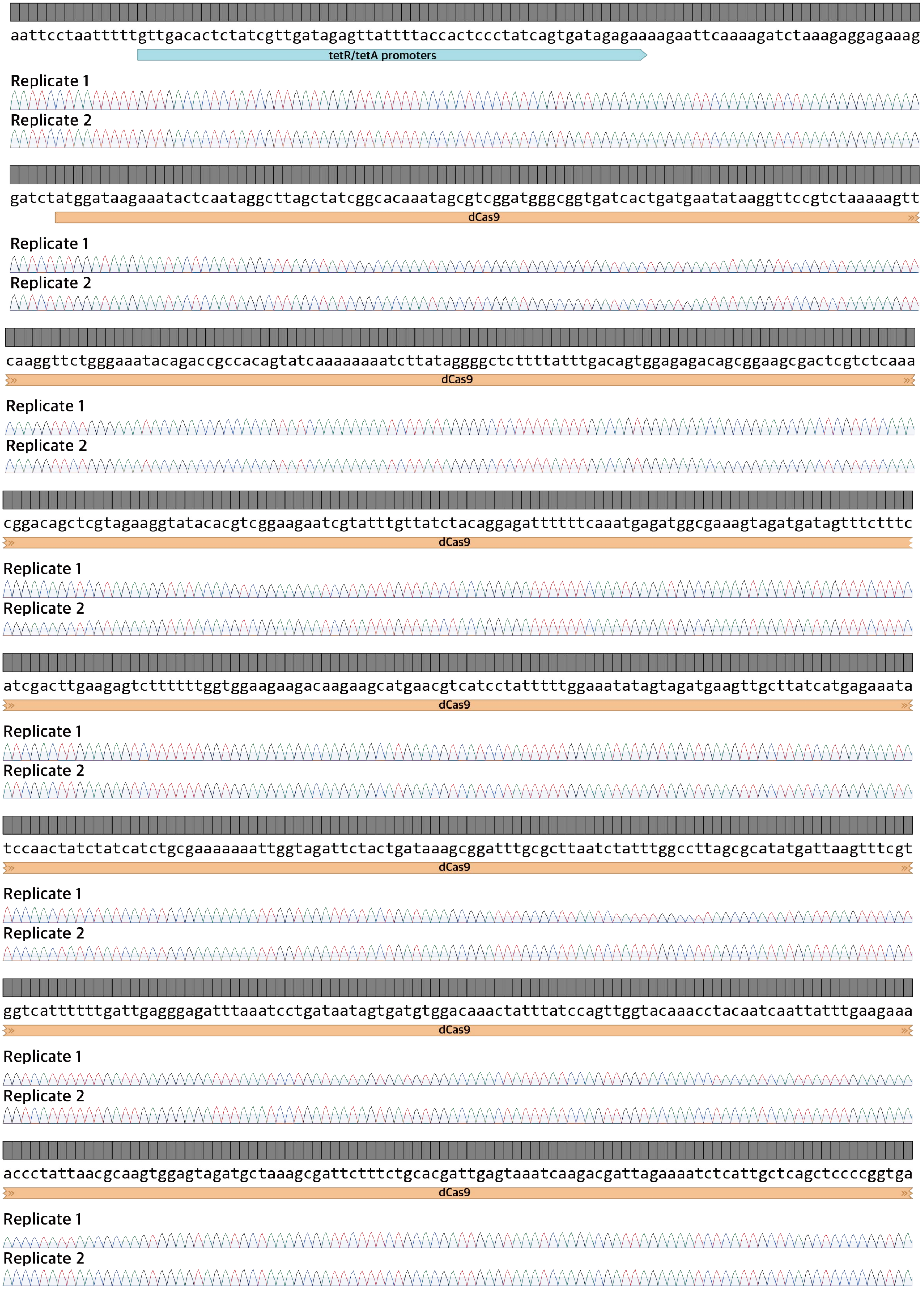

aattcctaatttttgttgacactctatcgttgatagagttattttaccactccctatcagtgatagagaaaagaattcaaaagatctaaagaggagaaag
tetR/tetA promoters
Replicate 1
Replicate 2
gatctatggataagaaatactcaataggcttagctatcggcacaaatagcgtcggatgggcggtgatcactgatgaatataaggttccgtctaaaaagtt
dCas9
Replicate 1
Replicate 2
caaggttctgggaaatacagaccgccacagtatcaaaaaaaatcttataggggctcttttatttgacagtggagagacagcggaagcgactcgtctcaaa
dCas9
Replicate 1
Replicate 2
cggacagctcgtagaaggtatacacgtcggaagaatcgtatttgttatctacaggagatttttcaaatgagatggcgaaagtagatgatagtttctttc
dCas9
Replicate 1
Replicate 2
atcgacttgaagagtctttttggtggaagaagacaagaagcatgaacgtcatcctatttttggaaatatagtagatgaagttgcttatcatgagaaata
dCas9
Replicate 1
Replicate 2
tccaactatctatcatctgcgaaaaaaattggtagattctactgataaagcggatttgcgcttaatctatttggccttagcgcatatgattaagtttcgt
dCas9
Replicate 1
Replicate 2
ggtcatttttgattgagggagatttaaatcctgataatagtgatgtggacaaactatttatccagttggtacaaacctacaatcaattatttgaagaaa
dCas9
Replicate 1
Replicate 2
accctattaacgcaagtggagtagatgctaaagcgattctttctgcacgattgagtaaatcaagacgattagaaaatctcattgctcagctccccggtga
dCas9
Replicate 1
Replicate 2

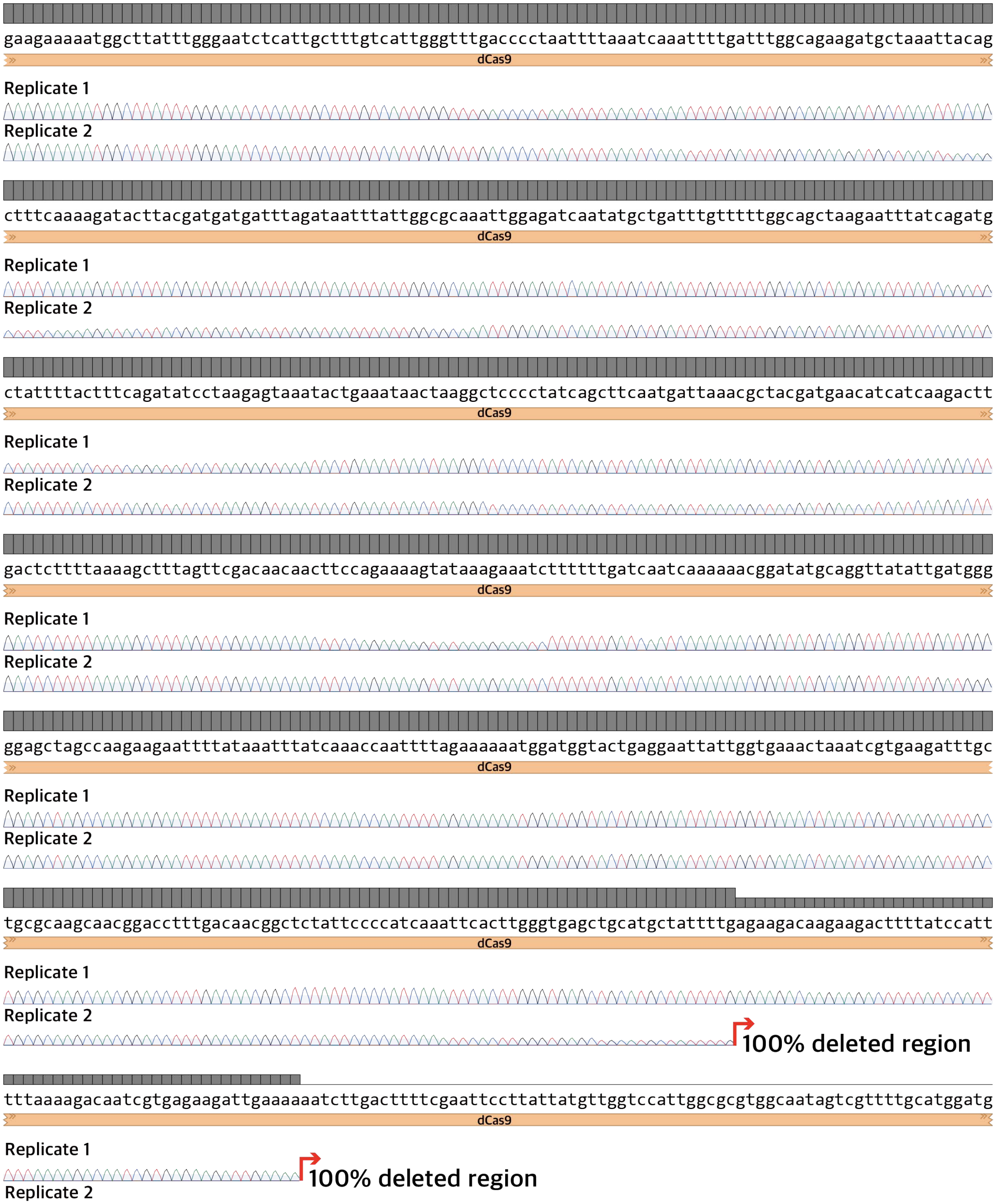

Figure S27. Plasmid map of *ftsZ* targeting CRIPSR-Cas expression vector and the next generation sequencing results of the plasmids sampled from *ftsZ* sgRNA-expressing *E. coli*. (a) A schematic representation of the plasmid construction and analysis regions. The blue region represents the coding sequence (CDS) of dCas9, the green region represents the expression cassette for *ftsZ* targeting single guide RNA (sgRNA), the red region indicates the region with 100% deletion in replicate 1, and the yellow region represents the region with 100% deletion in replicate 2. (b) Alignment of the merged sequences obtained through Next-Generation Sequencing (NGS) revealed that only a partial alignment was observed between the dCas9 expression promoter and the dCas9 CDS in the plasmid.

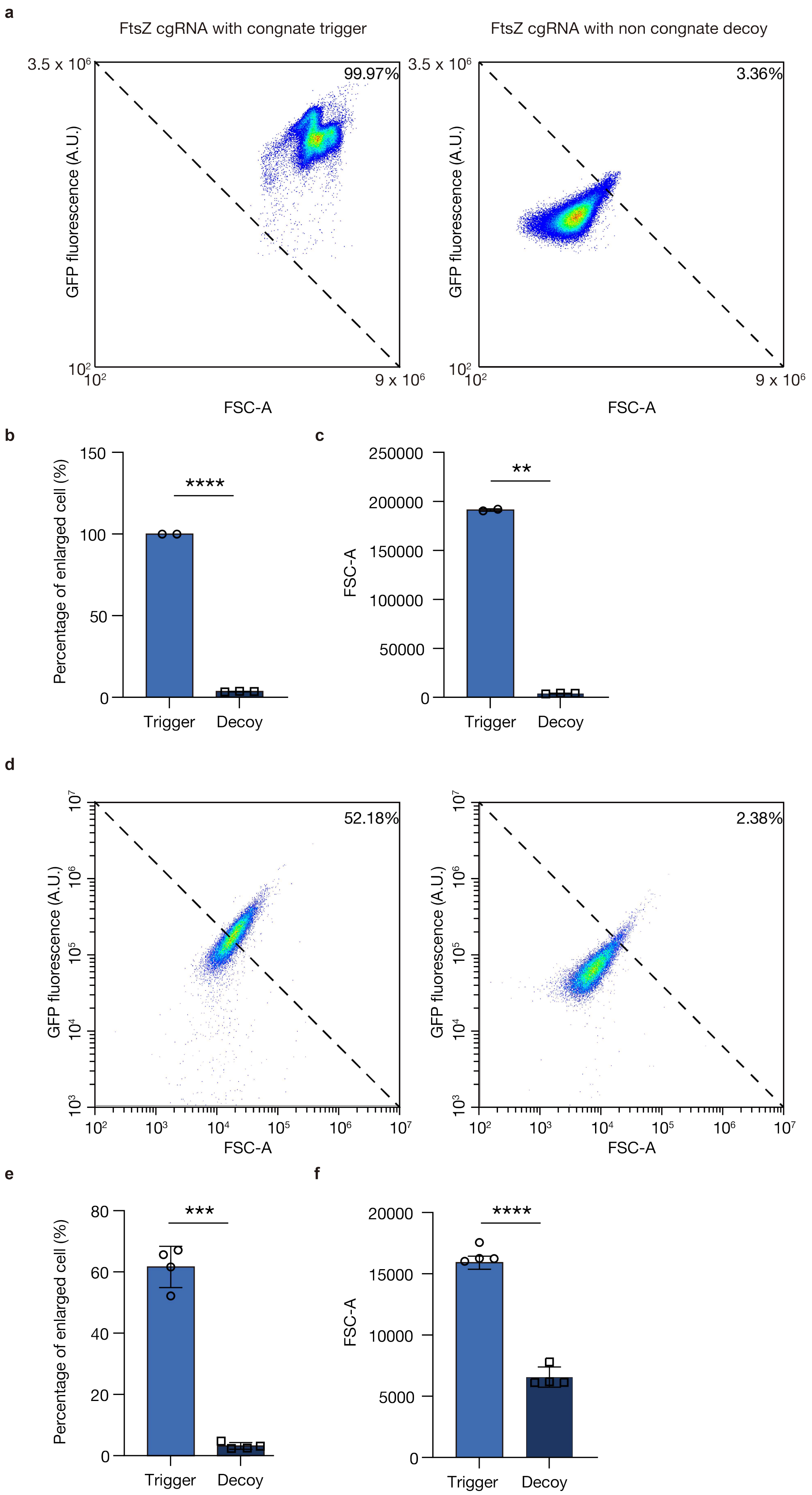
a
FtsZ cgRNA with congnate trigger
FtsZ cgRNA with non congnate decoy
3.5 x 10^6
99.97%
3.36%
GFP fluorescence (A.U.)
10^2
9 x 10^6
FSC-A
b
Percentage of enlarged cell (%)
****
Trigger
Decoy
c
FSC-A
**
d
52.18%
2.38%
e
***
f
****

Figure S28. Flow cytometry analysis of cell morphology regulation by *ftsZ*-targeting cgRNA or *mreB*-targeting cgRNA. (a) Flow cytometry analysis of cells with *ftsZ*-targeting cgRNA with cognate trigger(left) or decoy RNAs (right). The percentages of elongated cells (those with high FSC-A value and GFP fluorescence) are indicated at the top right side of each plot. The samples are gated with GFP before plotting, to filter out debris and dead cells. (b) The percentages of elongated cells in the logical TRUE condition (trigger induced, blue) and FALSE condition (decoy induced, dark blue). (c) The geometric mean FSC-A values of cells in the logical TRUE and FALSE conditions, respectively. (d) Flow cytometry analysis of cells with *mreB*-targeting cgRNA with cognate trigger (left) or decoy RNAs (right). The proportions of enlarged cells (those with high FSC-A value and GFP fluorescence) are indicated at the top right side of each plot. (e) The percentages of enlarged cells in the logical TRUE condition (trigger induced, blue) and FALSE condition (decoy induced, dark blue). (f) The geometric mean of FSC-A of cells in logical TRUE and FALSE conditions, respectively. Based on Welch's t-tests, **$P<0.01$, ***$P<0.001$ and ****$P<0.0001$, indicate conditions where the FSC-A of the logical TRUE cases is statistically significantly different from that of the highest of the logical FALSE cases.

a

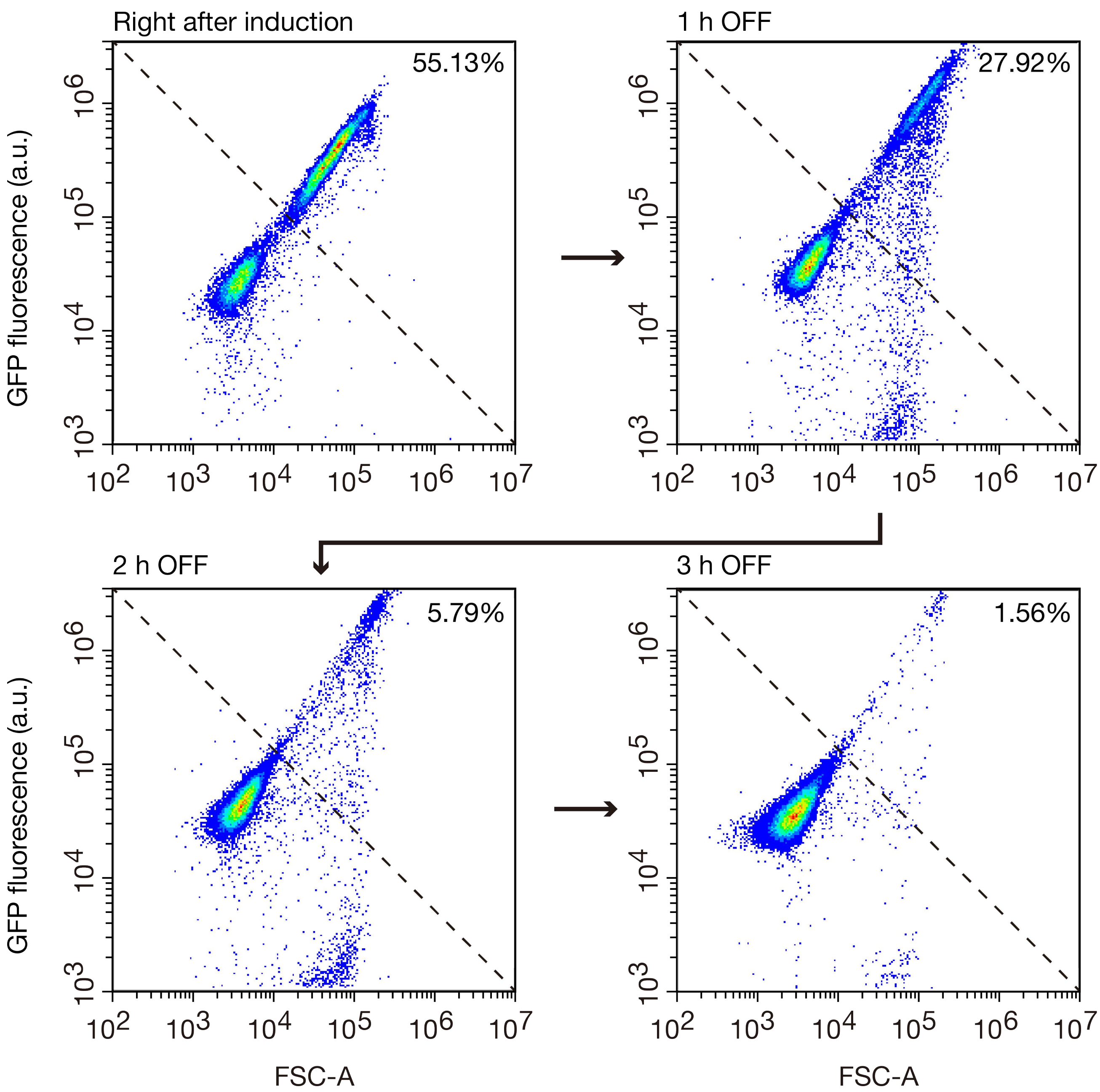

Right after induction
55.13%
1 h OFF
27.92%
2 h OFF
5.79%
3 h OFF
1.56%
GFP fluorescence (a.u.)
FSC-A
10^2
10^3
10^4
10^5
10^6
10^7

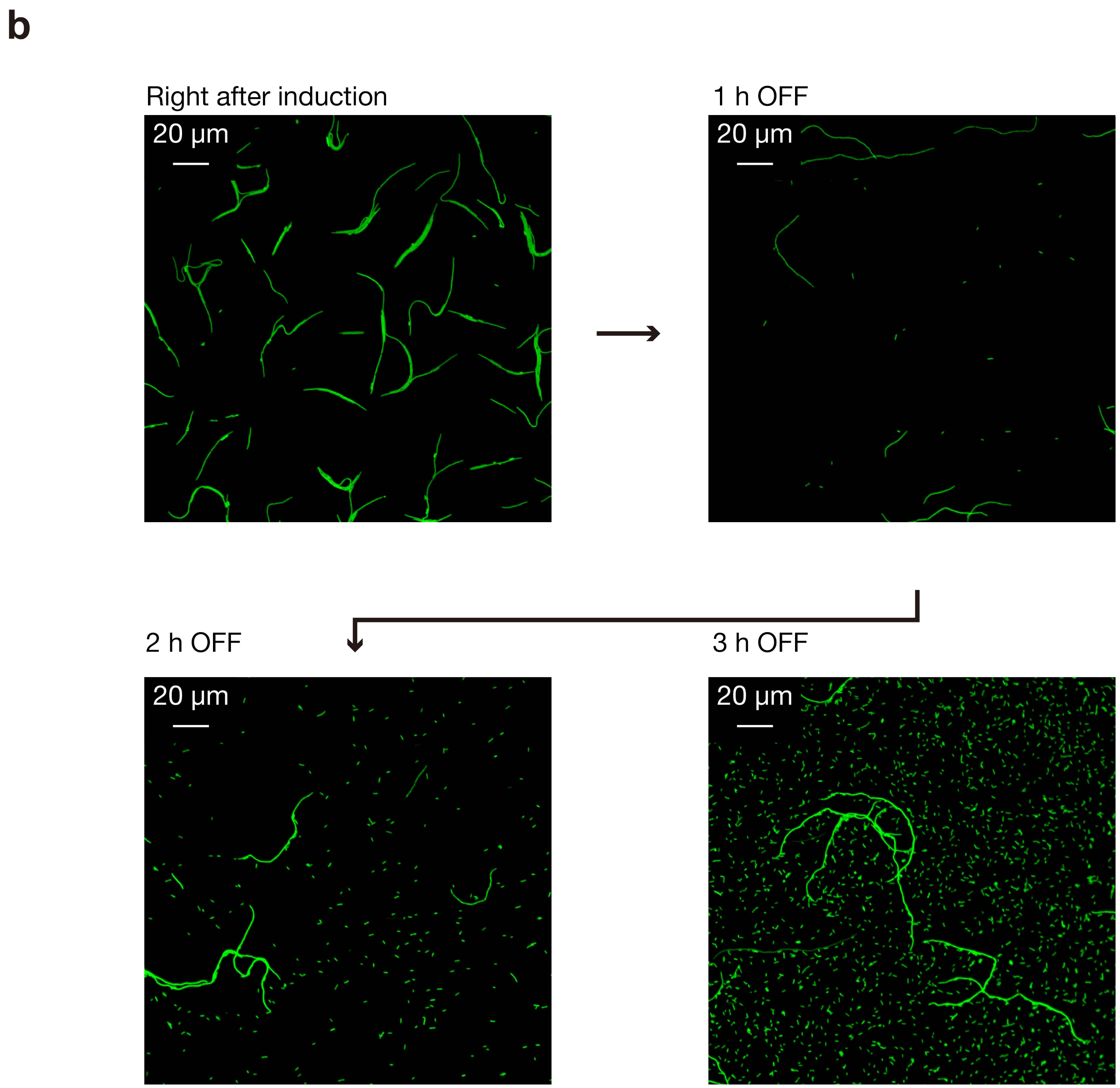

Figure S29. Flow cytometry analysis of reversible cell morphology regulation by *ftsZ*-targeting cgRNA. (a) Flow cytometry analysis of cells with *ftsZ*-targeting cgRNA with cognate trigger or decoy RNAs. The percentages of elongated cells (those with high FSC-A value and GFP fluorescence) are indicated at the top of right side of each plot. The samples are gated with GFP before plotting, to filter out debris and dead cells. (b) Representative images of filamentation-induced cells (3h, upper-left), and inducer-washed cells incubated 1h after induction (upper-right), 2h after induction (lower-left), and 3h after induction (lower-right).

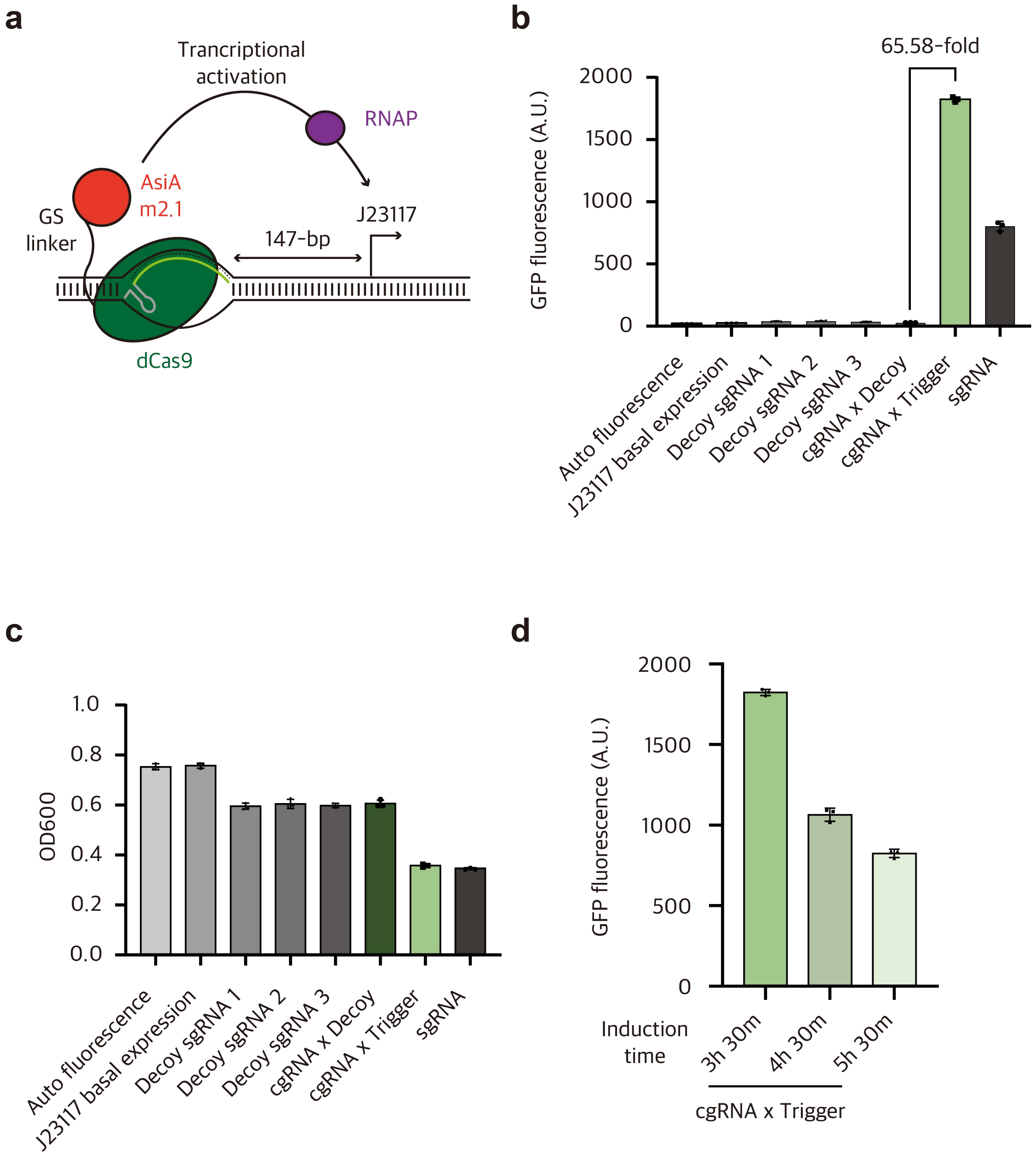

Figure S30. Regulation of GFP by combining a transcription activating Cas9 effector protein with cgRNAs. (a) The schematic of a Cas9-activator mediated transcription activation using an activated sgRNA, which guides activator to the promoter-adjacent region. (b) GFP fluorescence measured by flow cytometry at 3h 30m after induction of cgRNA activation with transcription activating Cas9 effector protein, instead of dCas9. Various sgRNAs expressed (decoy sgRNA1, 2, 3 and H4 sgRNA) or H4 cgRNA expressed with decoy or trigger RNAs. Only H4 cgRNA, along with its cognate trigger and H4 sgRNA, exhibited an increase in GFP expression. This suggests that our cgRNA-mediated Cas9 activator regulation could be applied to upregulate gene expression mediated by weak promoters. (c) Relative growths of various genetic circuit-expressing *E. coli* were analyzed using OD600 absorbance measured by microplate reader. (d) GFP fluorescence measured by flow cytometry at different time points (3h 30m, 4h 30m and 5h 30m) were shown.

References

1. Lu, Z.J., Turner, D.H. and Mathews, D.H. (2006) A set of nearest neighbor parameters for predicting the enthalpy change of RNA secondary structure formation. *Nucleic Acids Research*, **34**, 4912-4924.
2. Mathews, D.H., Sabina, J., Zuker, M. and Turner, D.H. (1999) Expanded sequence dependence of thermodynamic parameters improves prediction of RNA secondary structure11Edited by I. Tinoco. *Journal of Molecular Biology*, **288**, 911-940.
3. Turner, D.H. and Mathews, D.H. (2009) NNDB: the nearest neighbor parameter database for predicting stability of nucleic acid secondary structure. *Nucleic Acids Research*, **38**, D280-D282.
4. Serra, M.J. and Turner, D.H. (1995), *Methods in Enzymology*. Academic Press, Vol. 259, pp. 242-261.
5. Zuker, M. (2003) Mfold web server for nucleic acid folding and hybridization prediction. *Nucleic Acids Research*, **31**, 3406-3415.
6. Qi, Lei S., Larson, Matthew H., Gilbert, Luke A., Doudna, Jennifer A., Weissman, Jonathan S., Arkin, Adam P. and Lim, Wendell A. (2013) Repurposing CRISPR as an RNA-Guided Platform for Sequence-Specific Control of Gene Expression. *Cell*, **152**, 1173-1183.
7. Wolfe, B.R. and Pierce, N.A. (2015) Sequence Design for a Test Tube of Interacting Nucleic Acid Strands. *ACS Synthetic Biology*, **4**, 1086-1100.
8. Komura, R., Aoki, W., Motone, K., Satomura, A. and Ueda, M. (2018) High-throughput evaluation of T7 promoter variants using biased randomization and DNA barcoding. *PLOS ONE*, **13**, e0196905.
